# Supplementary material for: Genome assembly of the Pink Ipê (Handroanthus impetiginosus, Bignoniaceae), a highly valued, ecologically keystone Neotropical timber forest tree
Source: Gigascience. 2017 Dec 13;7(1):gix125. doi: 10.1093/gigascience/gix125 (PMC5905499; doi:10.1093/gigascience/gix125)
Supplement: GIGA-D-17-00159_Original_Submission.pdf [file gix125_giga-d-17-00159_original_submission.pdf]

GigaScience

Genome assembly of the pink Ipê (Handroanthus impetiginosus, Bignoniaceae), a highly-valued ecologically keystone Neotropical timber forest tree

--Manuscript Draft--

**Manuscript Number:**	GIGA-D-17-00159
**Full Title:**	Genome assembly of the pink Ipê (Handroanthus impetiginosus, Bignoniaceae), a highly-valued ecologically keystone Neotropical timber forest tree
**Article Type:**	Data Note
**Funding Information:**	CNPq (471366/2007-2) — Professor Rosane Garcia Collevatti<br>CNPq (457406/2012-7) — Professor Rosane Garcia Collevatti<br>CNPq (476709/2012-1) — Dr Evandro Novaes<br>FAP-DF (193.000.570/2009) — Dr Dario Grattapaglia
**Abstract:**	Background: Handroanthus impetiginosus (Mart. ex DC.) Mattos is a keystone Neotropical hardwood tree widely distributed in seasonally dry tropical forests of South and Mesoamerica. Regarded as the "new mahogany", it is the second most expensive timber and the most logged species in Brazil, currently under significant illegal trading pressure. The plant produces large amounts of quinoids, specialized metabolites with documented antitumorous and antibiotic effects. The development of genomic resources is needed to better understand and conserve the diversity of the species, to empower forensic identification of the origin of timber trade and to identify genes for important metabolic compounds.<br><br>Findings: The genome assembly covered 503.7Mb (N50=81,316 bp), 90.4% of the 557 Mbp genome, with 13,206 scaffolds. A repeat database with 1,508 sequences was developed allowing masking ~31% of the assembly. Depth of coverage analysis indicated redundancy in the consensus determination due to the extensive heterozygosity of the diploid sequence. Automatic gene prediction has provided 31,688 structures and 35,479 mRNA transcripts. To evaluate the biosynthetic potential of the plant, we used the genomic sequence and the comprehensive gene content annotation to identify genes related to the production of specialized metabolites.<br><br>Conclusions: This genome assembly is the first well-curated resource for a Neotropical forest tree and the first one for a member of the Bignoniaceae family, opening exceptional opportunities to empower evolutionary, molecular, phytochemical and breeding studies. This work should inspire the development of similar genomic resources for the largely neglected forest trees of the mega-diverse tropical biomes.
**Corresponding Author:**	Rosane Garcia Collevatti, PhD<br>Universidade Federal de Goias<br>Goiania, GO BRAZIL
**Corresponding Author Secondary Information:**	
**Corresponding Author's Institution:**	Universidade Federal de Goias
**Corresponding Author's Secondary Institution:**	
**First Author:**	Rosane Garcia Collevatti, PhD
**First Author Secondary Information:**	
**Order of Authors:**	Rosane Garcia Collevatti, PhD<br>orzenil.silva@embrapa.br B Silva-Junior, PhD<br>Evandro Novaes, PhD

Powered by Editorial Manager® and ProduXion Manager® from Aries Systems Corporation

	Dario Grattapaglia, PhD
**Order of Authors Secondary Information:**	
**Opposed Reviewers:**	
**Additional Information:**	
**Question**	**Response**
Are you submitting this manuscript to a special series or article collection?	No
**Experimental design and statistics**<br><br>Full details of the experimental design and statistical methods used should be given in the Methods section, as detailed in our Minimum Standards Reporting Checklist. Information essential to interpreting the data presented should be made available in the figure legends.<br><br>Have you included all the information requested in your manuscript?	Yes
**Resources**<br><br>A description of all resources used, including antibodies, cell lines, animals and software tools, with enough information to allow them to be uniquely identified, should be included in the Methods section. Authors are strongly encouraged to cite Research Resource Identifiers (RRIDs) for antibodies, model organisms and tools, where possible.<br><br>Have you included the information requested as detailed in our Minimum Standards Reporting Checklist?	Yes
**Availability of data and materials**<br><br>All datasets and code on which the conclusions of the paper rely must be either included in your submission or deposited in publicly available repositories (where available and ethically appropriate), referencing such data using a unique identifier in the references and in the "Availability of Data and Materials" section of your manuscript.<br><br>Have you have met the above requirement as detailed in our Minimum Standards Reporting Checklist?	Yes

Powered by Editorial Manager® and ProduXion Manager® from Aries Systems Corporation

Manuscript

Click here to download Manuscript Data_Note_Main_Text_H.impetiginosus_genome.docx

Genome assembly of the pink Ipê *(Handroanthus impetiginosus*, *Bignoniaceae*), a highly-valued ecologically keystone Neotropical timber forest tree

Orzenil Bonfim da Silva-Junior[1,2], Dario Grattapaglia[1,2], Evandro Novaes[3], Rosane G. Collevatti[4*]

[1]EMBRAPA Recursos Genéticos e Biotecnologia, EPqB, Brasília, DF. 70770-910. Brazil.

[2]Programa de Ciências Genômicas e Biotecnologia – Universidade Católica de Brasília, SGAN 916 Modulo B, Brasilia, DF 70790-160. Brazil

[3]Escola de Agronomia, Universidade Federal de Goiás. Goiânia, GO. 74001-970. Brazil.

[4]Laboratório de Genética & Biodiversidade, Instituto de Ciências Biológicas, Universidade Federal de Goiás. Goiânia, GO. 74001-970. Brazil.

***Corresponding author:** Rosane Garcia Collevatti, Instituto de Ciências Biológicas, Universidade Federal de Goiás, 74001-970, Goiânia, GO, Brasil.

E-mail: rosanegc68@hotmail.com. Phone: +55 62 3521-1729.

33

Abstract

Background: *Handroanthus impetiginosus* (Mart. ex DC.) Mattos is a keystone Neotropical hardwood tree widely distributed in seasonally dry tropical forests of South and Mesoamerica. Regarded as the "new mahogany", it is the second most expensive timber and the most logged species in Brazil, currently under significant illegal trading pressure. The plant produces large amounts of quinoids, specialized metabolites with documented antitumorous and antibiotic effects. The development of genomic resources is needed to better understand and conserve the diversity of the species, to empower forensic identification of the origin of timber trade and to identify genes for important metabolic compounds.

Findings: The genome assembly covered 503.7Mb (N50=81,316 bp), 90.4% of the 557 Mbp genome, with 13,206 scaffolds. A repeat database with 1,508 sequences was developed allowing masking ~31% of the assembly. Depth of coverage analysis indicated redundancy in the consensus determination due to the extensive heterozygosity of the diploid sequence. Automatic gene prediction has provided 31,688 structures and 35,479 mRNA transcripts. To evaluate the biosynthetic potential of the plant, we used the genomic sequence and the comprehensive gene content annotation to identify genes related to the production of specialized metabolites.

Conclusions: This genome assembly is the first well-curated resource for a Neotropical forest tree and the first one for a member of the *Bignoniaceae* family, opening exceptional opportunities to empower evolutionary, molecular, phytochemical and breeding studies. This work should inspire the development of similar genomic resources for the largely neglected forest trees of the mega-diverse tropical biomes.

Keywords: heterozygous genome, RNA-seq, transposable elements, quinoids, *Bignoniaceae*

DATA DESCRIPTION

Context. The generation of plant genome assemblies has been a key driver for the development of powerful genomic resources, which in turn allowed gaining detailed insights into the evolutionary history of the species while empowering breeding and conservation efforts [1, 2]. Such advances took place first in model plant species [3] followed by the mainstream [4] and minor crops [5], and some major forest trees [6-9]. This approach has provided enormous insights into essential plant metabolic processes for survival across distinct lineages. However, more recently, the research about functional roles for specialized metabolites has acknowledged the importance of these compounds, many of them being phylogenetically restricted [10]. These findings have motivated the community to address the gap in the species-specific knowledge of specialized plant metabolism by the determination of the DNA sequences in the nuclear genome of, for instance, key medicinal plants [11, 12]. Innovation in this field has relied on the combination of high-throughput genomics, including massive parallel sequencing and arrays with animal and clinical studies to elucidate the mechanisms of target compounds as adjuvant therapies, to demonstrate the necessary formulations for its biological effects and to determine which substances are beneficial or toxic. Apart from recent reports of shallow transcriptome characterization using 454 pyrosequencing [13] and a low-coverage (11X) fragmented genome assembly [14], essentially no well-curated genome assembly and gene content annotation exist for Neotropical forest trees, despite their recognized value by indigenous communities for their healing properties, increasingly exploited by large pharmaceutical corporations [15, 16]. An example of such tree is the species *Handroanthus impetiginosus* (Mart. ex DC.) Mattos (syn. *Tabebuia impetiginosa*, Bignoniaceae), popularly known as Pink Ipê, Lapacho or Pau d'arco, a source of both high value timber and traditional medicine. The species has virtually no genomic tools and resources, beyond a relatively small numbers of microsatellites with their caveats for more sophisticated genetic analyses. Whole-genome sequencing has now become accessible to a point that efforts to develop improved genomic resources for such species are possible and warranted. We built a preliminary assembly of the nuclear genome of a single individual of *Handroanthus impetiginosus* based on short-reads and longer mate-pair DNA sequence data to provide the necessary framework for the development of genomic resources to support multiple genomic and genetic analyses of this

keystone Neotropical hardwood tree regarded as the "new mahogany". It is the second most expensive timber and the most logged species in Brazil [17], exported largely to North America for residential decking and currently under significant illegal trading pressure. Additionally, the tree produces large amounts of natural products such as those of quinoid systems (1,4-anthraquinones, 1,4-naphthoquinones, and 1,2-furanonaphthoquinones), specialized metabolites with promising antitumorous, anti-inflammatory and antibiotic effects [18, 19]. The high pressure of logging and illegal trading on this species with a notable ecological keystone status urges conservation efforts of existing populations.

METHODS

Sample collection and sequencing. DNA of a single adult tree of *H. impetiginosus* (UFG-1) (Fig. S1) was extracted using Qiagen DNeasy Plant Mini kit (Qiagen, DK). Flow cytometry was used to check the genome size of tree UFG-1 indicating a genome size of (557 ±39) Mb /1C (Fig. S2) consistent with published estimates [20]. Total genomic DNA samples from other six unrelated trees were also extracted and pooled in equimolar amounts to be sequenced. Total RNA from shoots of five seedlings and from the differentiating xylem of the adult tree (UFG-1) was extracted using Qiagen RNeasy Plant Min kit (Qiagen, DK) and pooled for RNA sequencing. DNA and RNA sequencing was performed at the High-Throughput Sequencing and Genotyping Center of the University of Illinois Urbana-Champaign, USA. The following libraries were generated for sequencing: (1) two shotgun genomic libraries of short fragments (300bp and 600bp) from tree UFG-1 (2) one shotgun library from combined pools of five RNA samples tagged with a single index sequence; (3) one shotgun library from a equimolar pool of DNA samples from six unrelated trees tagged with a single index sequence. Paired-end sequencing, 2x150 nt, was performed in two lanes of an Illumina HiSeq 2500 instrument (Illumina, CA, USA). Three additional mate-pair libraries (jumps of 4kb to 5.5kb, 8kb to 10kb and 15kb to 20kb) for UFG-1 were also sequenced in two lanes of an Illumina HiSeq 2000 instrument (2x101 bp). This long-range sequence resource was used to generate the final genome assembly for annotation. A complete overview of the genome assembly and annotation pipeline is provided (Fig. S3). All individual plants used in this work were not collected in conservation units or private areas and thus did not require any license.

Genome assembly using short paired-end and mate pair sequencing data. Short reads and mate-pair reads were stripped of sequencing adapters using *Fastq-mcf* [21]. Reads that mapped to a database containing mitochondrial and chloroplast genomes of plants with *Bowtie1* [22] (option –v 3 –a –m 1) were discarded. Mate-pair reads were inspected using a *Perl* script, and sequences that did not contain the circularization adaptor were discarded. By using the filtered short reads, Jellyfish2 [23] and GenomeScope [24] were applied to obtain estimates of the *H. impetiginosus* genome size, repeat fraction and heterozygosity prior to the assembly. *ALLPATHS-LG* [25] was used for *de novo* assembly of the sequence data from both paired-end and mate-pair data, with default options, in a stepwise strategy for error correction of reads, handling of repetitive sequences and use of jumping libraries.

Transposable elements and repetitive DNA. Repetitive elements were detected and annotated on the genome assembly with the RepeatModeler *de novo* repeat family identification and modeling package [26]. Using RECON, RepeatScout and Tandem Repeat Finder, repetitive sequences were detected in the scaffolds longer than 10 kb using a combination of similarity-based and *de novo* approaches. The TE sequences were evaluated using modeling capabilities of the RepeatModeler program, with default settings, to compare the TE library against the entire assembled sequences and to refine and classify consensus models of putative interspersed repeats. A complementary analysis intended to augment the number of TE sequences classified according to current criteria [27] was performed using the PASTEC program [28]. RepeatMasker Open-4.0 [29] was used with the sequences from the *de novo* repetitive element library to annotate the interspersed repeats and to detect simple sequence repeats (SSRs) on the genome assembly.

Protein-coding genes annotation. Protein-coding genes annotation was performed with a pipeline that combines RNA-seq assembled transcript and protein alignments to the reference with *de novo* predictions methods (Fig. S1). RNA-Seq reads were screened for the presence of adapters, which were removed using *Fastq-mcf* [21]. *Trimmomatic* [30] was used to (1) remove low quality, no base called segments (N's) from sequencing reads; (2) scan the read with a 4-base sliding window, cutting when the average quality per base dropped below 15; and (3) remove reads shorter than 32 bp after trimming. Trimmed reads mapped to mitochondrial, chloroplast and ribosomal sequences from plants with *Bowtie1* [22] (options –v 3 –a –m 1) were

also removed. Transcript *de novo* assemblies were performed using *SOAP-Transdenovo* [31] and *Trinity de-novo* [32] from the processed reads. The assemblies were concatenated and used as input to *EvidentialGene* [33], a comprehensive transcriptome pipeline to identify likely complete coding regions and their proteins in the final, combined, transcriptome assembly. Gene modeling was carried out using standard procedures and tools described, for instance, in [34]. In summary, a genome-guided transcriptome assembly of *H. impetiginosus* was performed with the JGI PERTRAN RNA-seq Read Assembler pipeline [35] using both the RNA-Seq trimmed reads and sequences from the *de novo* transcript assembly. Loci were identified by the assembled transcript alignments using BLASTX [36] and EXONERATE [37] alignments of peptide sequences to the repeat-soft-masked genome using RepeatMasker [38], based on a transposon database developed as part of this genome assembly annotation. Known peptide sequences included manually curated data sets for plant species available from UniProtKB/Swiss-Prot [39] and sequences available from Phytozome [1] version 11 for *Arabidopsis thaliana*, *Oryza sativa*, *Erythranthe guttata*, *Solanum lycopersicum*, *Solanum tuberosum*, *Populus trichocarpa* and *Vitis vinifera*. Gene structure were predicted by homology-based predictors, FGENESH++, FGENESH_EST [40, 41] and GenomeScan [42]. Gene predictions were improved by PASA [43], including adding UTRs, correcting splicing and adding alternative transcripts. PASA-improved gene model peptides were subjected to peptide homology analysis with the above-mentioned proteomes to obtain Cscore values and peptide coverage. Cscore is the ratio of the peptide BLASTP score to the mutual best hit BLASTP score, and peptide coverage is the highest percentage of peptide aligned to the best homolog. A transcript was selected if its Cscore value was greater than or equal to 0.5 and its peptide coverage was greater than or equal to 0.5 or if it had transcript coverage but the proportion of its coding sequence overlapping repeats was less than 20%. For gene models where greater than 20% of the coding sequence overlapped with repeats, the Cscore value was required to be at least 0.9 and homology coverage was required to be at least 70% to be selected. Selected gene models were then subjected to classification analysis using *InterProScan 5* [44] for PFAM domains, PANTHER, Enzyme Comissioned Number (EC) and KEGG categories. Gene ontology annotation was obtained, where possible, from Interpro2GO and EC2GO mappings.

DATA VALIDATION AND QUALITY CONTROL

Global properties of the H. impetiginosus tree genome from the unassembled reads. Sequencing of the *H. impetiginosus* tree genome generated c. 599 million reads, comprising 73 Gbp of sequence data. This represents nearly 132× the expected sequence coverage. After removal of adaptors, followed by standard error correction and trimming with ALLPATHS-LG, with default options, c. 38 Gbp in 114,415,795 reads were found useful for the assembly process, yielding sequencing coverage of 91x (63x from the fragments libraries and 19x from the jumping libraries). The estimated physical coverage was 400x based on the observed fragment size distributions (Table S1). ALLPATHS-LG k-mer spectrum frequency analysis (at K=25) on useful reads, error corrected reads, estimated a haploid genome size of 540,968,531 bp, a repeat fraction of 38.0%, and a SNP rate of 1/88 bp (1.14%). An alternative analysis of the k-mer frequencies using GenomeScope [24] produced a haploid estimate of 503,748,072 bp, repetitive content of 36.6% and SNP rate of 1/60 bp (1.65%). Both estimates (Fig. 1A) are consistent with the flow cytometry estimates and in line with the expectations regarding the heterozygous content of the *H. impetiginosus* genome, a predominantly outcrossed tree [45]. Sequencing errors caused an extreme peak at k = 1 in the k-mer frequency distribution. Both k-mer histograms display two distinct peaks comprising the largest area of each histogram at depths 27 and 55. The bimodal distributions characterize the expected behavior for k-mer frequencies of a heterozygous diploid genome as seen, for example, in the recently reported Oak genome [46]. In the right homozygous peak (at K=55), k-mers are shared between the two homologous chromosomes. The left or heterozygous peak, with half the k-mer depth of the homozygous peak, contains k-mers that are unique to each haplotype due to heterozygosity. The difference in height between these peaks (heterozygous/homozygous ratio) is a measure of the heterozygosity within the genome, which is 1.65% according to the GenomeScope modeling equation.

Genome assembly. State-of-the-art haploid genome assembler pipelines from short-reads ALLPATHS-LG [25] and SOAPdenovo2 [47] were considered for an initial evaluation on the dataset of reads. Two relatively new algorithms specifically developed for de novo assembly of heterozygous genomes, MaSuRCA [48] and PLATANUS [49], were also attempted as alternatives to the other two assemblers designed for genomes of low heterozygosity. Reads were first preprocessed and error corrected using the algorithms provided by each assembler. PLATANUS was set to run but after 10 weeks it did not produce any result in an Intel(R) Xeon(R) server with

64 X7560 2.27GHz CPUs, 256 GB RAM, except for the k-mer count table on the input trimmed reads. After 9 week-long runtimes in an Intel(R) Xeon(R) server with 64 X7560 2.27GHz CPUs, 512 GB RAM, MaSuRCA successfully completed the generation of the super-reads from the trimmed reads but the process was aborted on the overlap-correction process in the Celera Assembler due to excessive CPU usage. SOAPdenovo2 ran very fast (3 days) but produced an assembly with total scaffold size of 860 Mbp. Analysis with SOAPdenovo2 was run with different k-mer sizes, from 31 to 71, step of 10, but none of them produced a reasonable assembly size in view of the expected size estimated by flow cytometry and the k-mer frequency. ALLPATHS-LG was therefore used to assemble the genome with default options. The short reads from fragmented libraries were error-corrected using default settings (K-mer size of 24, ploidy of 2), fragment-filled and assembled into initial unipaths (k-mer size of 96, ploidy of 2). Jumping reads from the mate-pair libraries were then aligned to the unipaths and all alignments were processed in a seed-extension strategy with junction point recognition within the read aimed to remove invalid and duplicate fragments to perform error correction and initial scaffolding. This initial process produced an assembly graph that was turned into scaffolds by analyzing branch points in the graph topology. This late process converted single-base mismatches into ambiguous base codes at branch. It also flattened some other structural features of the assembly including short indels. The contig assembly comprised 109,064 sequences with total length of 466,314,780 bp. The final assembly after scaffolding comprised 57,815 scaffolds with total length of 610,091,865 bp and N50 of 57 Kbp. The fraction of bases captured in gaps was 23.9% and the rate of ambiguous bases for all bases captured in the assembly was 0.24%.

Alternative scaffold and gap-filling. Although the ALLPATHS-LG performance was good in recovering the expected genome size in the assembled contigs there was a high fraction of the bases captured in gaps in the scaffolds (~ ¼ of the total genome assembly). *De novo* assembly algorithms applied to moderate-to-high levels of heterozygosity cannot match the performance achieved in assemblies of homozygous genomes, especially at the contig assembly level [50]. We thus used the assembled contigs to perform an alternative scaffolding step with SSPACE [51] using the error-corrected short fragment reads and the jumping reads. In this approach, genome assembly comprised 16,090 scaffolds with total length of 577,446,088 bp and N50 of 95 Kbp, respectively. The fraction of bases captured in gaps dropped from 23.9% to 18.9% in contrast to ALLPATHS-LG scaffolding, totaling 109,533,288 bp. The rate of ambiguous bases for all bases

captured in the assembly dropped from 0.24% to 0.13%. All preprocessed reads were reused in an attempt to close the intra-scaffold gaps using the *GapCloser* [52] algorithm from SOAPdenovo2. Final genome assembly after gap-filling was 586,206,884 bp in 15,671 scaffolds and only 20,583,469 bp (3.51% of the genome assembly) remained in 24,907 gaps. Scaffolds N50, with gaps, was 97,344 Kb (L50 = 1,792). Sequences longer than 20 kb were assembled in only 6,791 scaffolds totaling 538,102,146 bp, ~97% of the genome size estimated from cytometry (557 Mb).

Evaluation of accuracy of the genome assembly. A subset of fragments and jumping read pairs (~15x sequencing coverage each) were used to uncover inaccuracies in the genome assembly. Scaffolds with identified errors were broken or flagged for inspection. REAPR [53] was used to test each base of the genome assembly looking for small local errors (such as a single base substitutions, and short insertions or deletions) and structural errors (such as scaffolding errors) located by means of changes to the expected distribution of inferred sequencing fragments from the mapped reads using SMALT v0.7.6 [54]. REAPR reported that only 343,588,027 (~60%) bases in the assembly should be free of errors, with 5,476 reported (1,658 within contigs, 3,818 over gaps) in the remaining 242,618,857 bp. The most frequent (~92%) type of error reported was *Perfect_cov* and *Link*. *Perfect_cov* means low coverage of perfect uniquely mapping reads in this region while *Link* depicts situations in which significant proportion of the reads in the reported region mapped to elsewhere in the assembly. The recognition of this erroneous base call should thus reflect the repetitive nature of the genome as inferred from the k-mer frequency spectra analysis (~36-38% of repeats). Besides the base error calls due to repeats, other structural problems in the assembly were identified based on sequence-coverage differences from the expected fragment size distribution and the program used this information to break these. Given the high heterozygosity and divergence between haplotypes on this diploid genome sequence, homologous sequences can assemble separately or merge. Structural errors in REAPR were likely called at the boundaries of these regions. The final genome assembly after REAPR breaks had total size of 576,829,188 bp in 19,319 sequences. N50 size of scaffolds ≥ 1,000 nt dropped from 97,344 Kb (L50 = 1,792) to 71,491 bp (L50 = 2,379) and sequences of size ≥ 20,000 bp totaled 506,169,954 bp in 7,812 scaffolds. The number of remaining gaps in the assembly was 21,417 totaling 30,066,113 bp (5.05%). Paired-end reads from the short fragment libraries were aligned back independently to this genome assembly using SMALT (map -r 0 -x -y

0.5; default alignment penalty scores). Per-scaffold depth of coverage was computed, regardless of mapping quality, using GATK DepthofCoverage. The mean read depth across the scaffolds resulted in 66.45x. The mean read length of the mapped reads was 139.8 bp and the corresponding k-mer coverage for size of 25 was 55.04x which matches with the homozygous peak computed from the k-mer frequency distribution from the unassembled reads. The read depth frequencies are showed in Fig. 1B. The heterozygous/homozygous peak height (> 1) in the distribution suggests that the assembly contains redundant copies of unmerged haplotypes due to the structural heterozygosity of the diploid genome of the species.

Additional procedure was taken to assess the structural heterozygosity of the diploid genome probably resulting as separate scaffolds in the assembly. To specifically deal with the structural heterozygosity we introduced a step to, leniently, recognize and remove alternative heterozygous sequences. Scaffold sequences were aligned one versus all using BLAT [55] and results were concatenated in a single file of alignments and sorted. Similar sequences were identified on the base of pairwise similarity using filterPSL utility from AUGUSTUS [56] with default parameters, and retaining all best matches to each single sequence queried against all others that satisfy minimal percentage of identity (minId=92%) and minimal percentage of coverage of the query read (minCover=80%). We considered as heterozygous redundant scaffold sequences which showed pairwise similarity to any other sequence and their depth of coverage fell in a Poisson distribution with parameters given by the heterozygous peak of the read depth distribution over all scaffolds (lambda = 34; Fig. 1B). The final step in this kept only one copy – the largest one –of the heterozygous scaffolds among pairs with high similarity.

At the end of the accuracy evaluation processes, the final genome assembly had a total size of 503,314,177 bp, with gaps, in 13,206 scaffolds, which represent 476,667,120 bp in 24,300 contigs. The N50 of scaffolds was 80,946 bp (L50 = 1,906), the average size of the sequences was 38,112 bp and sequences of size ≥ 20,000 bp summed 461,203,002 bp (91.6%) in a total of 6,647 scaffolds. For the contigs, N50 was 39,657 bp (L50 = 3,581) and the average size was 19,624 bp. The remaining gaps comprised 26,447,057 bp (5.25% of the genome assembly) in 11,094 segments (average size of gaps was 2,384 bp). The total assembly size represents over 90% of the flow cytometry genome estimate (557 Mb) and should provide a good start to build a reference genome assembly of the species using long-range scaffolding techniques such as

whole genome maps using either imaging methods [57] or contact maps of chromosomes based on chromatin interactions [58]. Table 1 summarizes the main statistics of the *Handroanthus impetiginosus* genome assembly with respect to the decisions made in the assembly process.

A reassessment of the assembly accuracy was carried out using REAPR on the final genome assembly. A total of 121 errors within a contig were still recognized, a much smaller number than previously annotated (1,658 errors). Fig. 1C shows the frequency distribution for the read depth computed from the paired-end read alignment to the scaffolds sequences. It indicates the expected effect on the distribution in comparison to the previous redundant assembly. The height of the heterozygous peak was lowered by removing unmerged copies of the same heterozygous loci. Fig. 1D shows the relation between the observed number of scaffolds in the final assembly and their read coverage in comparison to a Poisson approximation with lambda = 63 which was the observed average sequencing coverage. The long tail at higher coverage reflects the repeats in the genome while the exceeding number of sequences at lower coverage indicates that redundant copies of unmerged heterozygous stretches of sequences are still present in the assembly. Our genome assembly metrics are similar to recent reports of genome assemblies of other highly heterozygous forest tree genomes. For oak, *Quercus robur*, the assembly resulted in a total of 17,910 scaffolds corresponding to *c*. 89% of the expected 740 Mbp genome size [59]. For *Quercus lobate*, 40,158 contigs remained after an haplotype-reducing procedure on the assembly, totaling 760 Mb, with an N50 of 95,000 bp [46]. This preliminary haplotype-reduced genome assembly for *Handroanthus impetiginosus* will be useful for SNP (single nucleotide polymorphism) discovery and calling, one of the downstream objectives for generating this genome assembly.

Repetitive DNA. A total of 1,608 consensus sequences (average length = 773 bp and totaling 1,281,536 bp) representing interspersed repeats in the genome assembly were found. Search for domains in these sequences with similarity to know large families of genes that could confound the identification of true repeats indicated 85 false positives in the consensus library of repeats. Further 50 sequences were annotated with predicted protein domains frequently associated with protein coding genes. These 135 sequences were wiped out from the consensus library. Mostly of the remaining 1,473 sequences (71.1%) could not find classification in the hierarchical well-known classes of Transposable Elements [60] but 16.6% could be classified as

Class I (retrotransposons) including three orders: LTR (12.8%), LINE (1.6%) and SINE (2.2%); 8.4% are Class II (DNA transposons). Other categories comprised non-autonomous TEs: TRIM (0.4%) and MITE (3.5%). Unknown non-classified sequences in the consensus library expand a wide range of sequence sizes from 42 bp up to 5,987 bp (average = 345 bp, median = 503 bp). The 1,473 sequences representing interspersed repeats in the consensus repeat library were used to mask the genome with RepeatMasker. The masked fraction of the genome assembly comprised 155,348,349 bp, 30.9% of the total assembled genome of 503 Mbp. More than 50% of the masked bases in the assembly, or 80 Mbp, came from unknown non-classified sequences in the consensus library. In the well-known repeats, retrotransposons are the most abundant class in the assembly comprising 50 Mbp (~1/3 of the masked bases) with prominence of LTR/Gypsy (~23 Mb) and LTR/Copy (18 Mb) families of repeats. DNA transposons and non-autonomous orders of transposons masked 12 Mbp and 11 Mbp (~1/6 of the masked bases), respectively, highlighting the prominence of DNA/hAT families of class II and MITE (Fig. 2). Simple sequence repeats (SSRs) detection using RepeatMasker identified a total of 182,115 microsatellites with a density of 2.76 kb per SSR in the genome assembly. This density corroborates the general finding that the overall frequency of microsatellites is inversely related to genome size in plant genomes [61]. This SSRs density in *H. impetiginosus* (genome size of 557 Mbp/SSR density of 362 per Mbp) is higher than in larger plant genomes such as those of maize (1,115 Mbp/163 SSRs per Mbp), *S. bicolor* (738 Mbp/175 per Mbp), *G. raimondii* (761 Mbp/74.8 per Mbp) [62] but lower than densities in smaller genomes such as those of *A. thaliana* (120 Mbp/ 418 per Mbp), *Medicago truncatula* (307 Mbp/ 495 per Mbp) and *C. sativus* (367 Mbp/ 552 per Mbp) [63]. Different SSR motifs ranging from 1 to 6 bp showed that the di-nucleotide repeats were the most abundant repeats followed by the mono- (Fig. S4A). The frequency of SSR decreased with increase in motif length (Supplementary Fig. S4B), which is a trend usually observed both in monocots and dicots [63].

Transcriptome assembly and gene content annotation and analysis. A single run of Illumina HiSeq 2500 sequencing, from a pool of RNA samples, generated nearly 148 million of paired end reads. After adapter removal, trimming and coverage normalization, 55.2 million high-quality reads (38%) were used to assemble the transcriptome using *de novo* (Trinity and SOAP-Trans-denovo transcripts combined with the EvidentialGene pipeline) and genome guided methods (PERTRAN). The PASA pipeline was used to integrate transcripts alignments to the genome

assembly from these set of sequences, generating 54,320 EST assemblies representing putative protein-coding loci in the genome assembly. Loci were identified by the assembled transcript alignments using BLASTX [36] and EXONERATE [37] alignments of plant peptides to the repeat-soft-masked genome using RepeatMasker. After gene model prediction and refinements, a total of 36,262 gene models were found in the genome assembly and 31,668 of them were retained after quality assessment based on Cscore, protein coverage, and overlap to repeats as described in Methods. The number of predicted mRNA transcripts was 35,479.

Structural features of the gene content are shown in Table 2 and Table 3. The average number of exons per gene was ~5 and its average length was 285 bp. The average number of introns per gene was ~4 and its average length was 445 bp. The GC content is significantly different between exons and introns (t-test p-value < 0.0001). Coding sequences have ~43% of GC, while introns have less with ~33% (Table 2). GC content tends to be higher in coding (exonic) than in non-coding regions [64], which may be related to gene architecture and alternative splicing [65-67]. A comparison of the gene features parameters, such as number and length, was carried out between *H. impetiginosus* and *Erythranthe guttata*, another plant in the order Lamiales (Asterids), the model plant *A. thaliana* and the model tree *P. trichocarpa* (Rosids). As showed in the frequency histograms, the exons parameters are stable among these species (Fig. S5B). For the introns (Supplementary Fig. S5C), frequency histograms have a sharp peak around 90 bp and a larger peak that is much lower in density. There is a small intron-size variability from species to species in the distributions, especially for larger introns, which rarely go beyond than 10,000 bp. The intron length distributions in these four species is similar to those observed in lineages that are late in the evolutionary time scale, such as plants and vertebrates [68]. The sharp peak in the distributions at their "minimal intron" size is supposed to affect function by enhancing the rate at which mRNA is exported from the cell nucleus [69, 70]. In the model plant *A. thaliana,* a minimal intron group was previously defined [69] as anything that lies within three standard deviations of the optimum peak at 89±12 bp (53 bp – 125 bp). According to this definition, Table 3 summarizes the distribution of the minimal intron among genes of *H. impetiginosus* and other selected plant species in the Asterids and Rosids lineages. We have calculated the percentages of minimal introns out of the total introns and the fraction of minimal-intron-containing genes with at least one minimal intron. Computed values were similar between *H. impetiginosus* and those of selected species with higher number of large introns (smaller minimal intron peak) but

were more distinctive with those species such as *A. thaliana* and *E. guttata* in which the number of large introns was lower (larger minimal intron peak). This is thought as a general trend and was also observed in previous work [69]. These comparative analyses about the structural properties of the predicted genes indicate that the genome assembly of *H. impetiginosus* contains highly accurate gene structures.

To further validate the gene content annotation, we used the transcript assemblies and selected plant proteomes to inspect if these sequences could align in its entirety to the genomic sequence. Out of the 31,668 primary mRNA transcripts (considering only the longest one when isoforms were predicted) in the genome, 11,488 have 100% of their CDS covered by EST assemblies. The remaining 20,054 transcripts have either a minimum of 80% of their CDS covered by EST assemblies or a cscore ≥ 0.5. From these latter, the encoded putative peptides have excellent sequence similarity support from BLASTP comparisons with dicot species *Erythranthe guttata* (5,224 genes), *Sesamum indicum* (4,625 genes), potato or tomato (2,777 genes), soybean (1,484 genes) and the poplar tree (1,424 genes) reflecting adequately the taxonomic relationship between *H. impetiginosus* and these other related dicots. Gene models support was also found from more distantly related dicots (1,826 genes) and monocots (1,042 genes). Altogether, 31,048 gene models (98%) show well-supported similarity hits at this e-value threshold to other known plant protein sequences. Additional 517 predicted protein sequences did not produce hits and 103 sequences produced ambiguous hits from non-target species or represent possible contaminants in the assembly such as endophytic fungi (ascomycetes, 42 sequences; basidiomycetes, 17 sequences). Fig. S6A summarizes the main finding regarding the similarity analyses with known proteins.

BUSCO [71] single-copy genes plant profiles were used to estimate completeness of the expected gene space as well as the duplicate fraction of the genome assembly. Out of the 956 profiles searched on the assembly, 59 were reported missing and 30 returned fragmented. From the profiles with complete match to the assembly, 867 (90.7%) were reported as single-copy and 247 (25.8%) were found duplicated (Fig. 3B). The fraction of duplicates in the BUSCO analysis was intended to estimate the level of redundancy in the genome assembly. However the frequency of small- and large-scale duplications, such as (paleo)polyploidy, in plants makes this feature less applicable [72]. To assess the validity of this observation in our analysis we

benchmarked our results by searching the BUSCO profiles on the Poplar 3.0 genome annotation [7]. The analysis reported completeness level of 96% (918 single-copy profiles with complete match) while duplicate level was 24.9% (238 profiles with duplicated complete match).

Databases for gene ontology (GO) annotation are rich resources to describe functional properties of experimentally derived gene sets. To explore relationships between the GO terms in the *H. impetiginosus* and related, well-curated, genomes we used WEGO [73] to perform a genome-wide comparative analyses among Level 2 GO terms with the model tree *P. trichocarpa.* The P-value of Pearson Chi-Square test was considered to indicate significant relationships between the gene number of each GO term in these two datasets and to suggest patterns of enrichment (Fig. S6). This analysis reveals several GO terms in which the number of genes in the two species were remarkably related. For the terms in which the comparison did not indicate a significant relationship of gene numbers between the two datasets, all the compared GO terms suggest enrichments in *P. trichocarpa* compared to *H. impetiginosus*, except for GO terms involved in binding and nutrient reservoir activity.

Genome-guided exploration of specialized metabolism genes of quinoid systems. Aside from its high valued wood, *H. impetiginosus* and other Ipê species are also known for their medicinal effects. Extracts from its bark and wood have many ethnobotanical uses: against cancer, malaria, fevers, trypanosomiasis, fungal and bacterial infections and stomach disorders [74, 75]. The wood extracts have also been demonstrated to have anti-inflammatory effects [76] [77]. The main bioactive components isolated from the Pink Ipê is Lapachol and its products [78], which are naphthoquinones derived from the o-succinylbenzoate (OSB) pathway [79]. Lapachol is also responsible for the well-known high resistance of the Ipê wood against rotting fungi and insects [80]. In addition, naphthoquinones are aromatic substances with ecological importance for the interaction of plants with other plants, insects and microbes [79]. Given their medicinal and biological relevance, we have searched the *H. impetiginosus* annotated genes for the enzymes involved in the biosynthesis of naphthoquinones. We have found all the important known enzymes that lead to the biosynthesis of lapachol (Fig. 4). Unfortunately, however, the last two steps of the lapachol biosynthesis pathway still constitute unidentified enzymes [79]. The number of *H. impetiginosus* genes encoding for the enzymes of each step in the pathway is comparable to the numbers found in other species. However, three exceptions were found. *H.*

impetiginosus has five genes encoding the enzyme that converts chorismate to isochorismate, the first step in the o-succinylbenzoate (OSB) pathway. Two other steps where *H. impetiginosus* were found to have relatively more genes are the ones that lead to the synthesis of 1,4-Dihydroxy-2-naphtoyl-CoA and of 2-Phytyl-1,4-naphthoquinone. The availability of sequences for these genes may open new avenues for biotechnological products and for a better understanding of their ecological roles.

RE-USE POTENTIAL

We have reported a quality genome assembly for *H. impetiginosus*, a highly valued, ecologically keystone tropical timber and a species rich in natural products. This is the first well-curated genome for a Neotropical forest tree and surely, the first one reported for a member of the Bignoniaceae family. Besides expanding comparative genomic studies by including an overlooked taxonomic family so far, the availability of this genome assembly will allow the development and application of robust and far-reaching sets of genome-wide SNP genotyping tools to support multiple population genomics analyses in *H. impetiginosus* and related species of the Tabebuia Alliance. This group includes several of the most ecologically and economically important timber species of the American tropics. Going beyond the species-specific significance of these results, this study paves the way for developing similar genomic resources for other Neotropical forest trees of equivalent relevance. This in turn will open exceptional prospects to empower a higher-level understanding of the evolutionary history, species distribution and population demography of the still largely neglected forest trees of the mega diverse tropical biomes. Furthermore, this genome assembly provides a new resource for advances in the current integration between genomics, transcriptomics and metabolomics approaches for exploration of the enormous structural diversity and biological activities of plant-derived compounds.

AVAILABILITY OF SUPPORTING DATA

Sequences for the genome and assembly along with gene content annotation as well as the raw sequencing reads have been deposited into GenBank, accession number PRJNA324125, BioSample SAMN05195323.

List of abbreviations

BLASTP, Basic Local Alignment Search Tool for Proteins; BLAT, BLAST-like alignment tool; CDS, coding DNA sequence; EC, Enzyme Comissioned Number; EST, Expressed Sequence Tag; GATK, Genome Analysis Toolkit; GO, Gene Ontology; LINE, Long Interspersed Nuclear Elements; LTR, Long Terminal Repeats; MBH, Mutual Best Hit; MITE, Miniature Inverted–Repeat Transposable Elements; mRNA, messenger RNA; PASA, Program to Assemble Spliced Alignment; REAPR, Recognition of Errors in Assemblies using Paired Reads; SINE, Short Interspersed Nuclear Elements; SNP, Single Nucleotide Polymorphism; SSPACE, SSAKE-based Scaffolding of Pre-Assembled Contigs after Extension; TE, transposable element.

Ethics approval

Not applicable

Consent for publication

Not applicable

Competing interests

The authors declare that they have no competing interests.

Funding and acknowledgements

This work was supported by competitive grants from CNPq to RGC (project no. 471366/2007-2 and Rede Cerrado CNPq/PPBio project no. 457406/2012-7), to EN (CNPq Proc. 476709/2012-1) and to DG (PRONEX FAP-DF Project Grant "NEXTREE" 193.000.570/2009). RGC and DG have been supported by productivity grants from CNPq, which we gratefully acknowledge. OBSJr has been supported by an EMBRAPA doctoral fellowship and was an Affiliate Researcher at Lawrence Berkeley National Laboratory (LBNL), Berkeley CA, at the time of this research. OBSJr thanks to DM Goodstein and the members of the Phytozome team at the LBNL/Joint Genome Institute (JGI) for their valuable help and support in working with the JGI pipelines for genomic research. WE also thank Dr. Gabriela Ferreira Nogueira and André Luis X. de Souza for their help with flow cytometry analysis.

Authors' contributions

OBSJr performed sequence data analysis and genome assembly and together with EN carried out transcriptome and protein-coding gene annotation. RC and DG conceived the project, collected samples, extracted genomic DNA and RNA, carried out flow cytometry analysis and supervised the project. All authors were involved in discussions, writing and editing. All authors read and approved the final manuscript.

REFERENCES

1. Goodstein DM, Shu SQ, Howson R, Neupane R, Hayes RD, Fazo J, Mitros T, Dirks W, Hellsten U, Putnam N *et al*: **Phytozome: a comparative platform for green plant genomics**. *Nucleic Acids Research* 2012, **40**(D1):D1178-D1186.
2. Kang YJ, Lee T, Lee J, Shim S, Jeong H, Satyawan D, Kim MY, Lee SH: **Translational genomics for plant breeding with the genome sequence explosion**. *Plant Biotechnology Journal* 2016, **14**(4):1057-1069.
3. Bevan M, Walsh S: **The Arabidopsis genome: A foundation for plant research**. *Genome Research* 2005, **15**(12):1632-1642.
4. Morrell PL, Buckler ES, Ross-Ibarra J: **Crop genomics: advances and applications**. *Nature Reviews Genetics* 2012, **13**(2):85-96.
5. Varshney RK, Glaszmann JC, Leung H, Ribaut JM: **More genomic resources for less-studied crops**. *Trends in Biotechnology* 2010, **28**(9):452-460.
6. Myburg AA, Grattapaglia D, Tuskan GA, Hellsten U, Hayes RD, Grimwood J, Jenkins J, Lindquist E, Tice H, Bauer D *et al*: **The genome of *Eucalyptus grandis***. *Nature* 2014, **510**(7505):356-362.
7. Tuskan GA, DiFazio S, Jansson S, Bohlmann J, Grigoriev I, Hellsten U, Putnam N, Ralph S, Rombauts S, Salamov A *et al*: **The genome of black cottonwood, *Populus trichocarpa* (Torr. & Gray)**. *Science* 2006, **313**(5793):1596-1604.
8. Neale DB, Wegrzyn JL, Stevens KA, Zimin AV, Puiu D, Crepeau MW, Cardeno C, Koriabine M, Holtz-Morris AE, Liechty JD *et al*: **Decoding the massive genome of loblolly pine using haploid DNA and novel assembly strategies**. *Genome Biology* 2014, **15**(3).
9. Nystedt B, Street NR, Wetterbom A, Zuccolo A, Lin YC, Scofield DG, Vezzi F, Delhomme N, Giacomello S, Alexeyenko A *et al*: **The Norway spruce genome sequence and conifer genome evolution**. *Nature* 2013, **497**(7451):579-584.
10. Moghe G, Last R: **Something old, something new: Conserved enzymes and the evolution of novelty in plant specialized metabolism**. *Plant Physiology* 2015:pp.00994.02015.
11. Stone R: **Lifting the Veil on Traditional Chinese Medicine**. *Science* 2008, **319**(5864):709-710.
12. Chappell J, DellaPenna D, O’Connor S: **Specific Aims for Medicinal Plant Genomics Resource**. *Medicinal Plants Genomics Resource* 2017.
13. Brousseau L, Tinaut A, Duret C, Lang T, Garnier-Gere P, Scotti I: **High-throughput transcriptome sequencing and preliminary functional analysis in four Neotropical tree species**. *BMC Genomics* 2014, **15**(1):238.

14. Olsson S, Seoane-Zonjic P, Bautista Ro, Claros G, González-Martínez S, Scotti I, Scotti-Saintagne C, Hardy O, Heuertz M: **Development of genomic tools in a widespread tropical tree, Symphonia globulifera L.f.: a new low-coverage draft genome, SNP and SSR markers**. *Molecular Ecology Resources* 2017, **17**(4):614-630.
15. Cadena-González A, Sorensen M, Theilade I: **Use and valuation of native and introduced medicinal plant species in Campo Hermoso and Zetaquira, Boyacá, Colombia**. *Journal of Ethnobiology and Ethnomedicine* 2013, **9**(1):23.
16. Bodker G, Bhat KKS, Burley J, Vantomme P: **Medicinal plants for forest conservation and health care**. *Food and Agriculture Organization of the United Nations* 1997.
17. Schulze M, Grogan J, Uhl C, Lentini M, Vidal E: **Evaluating ipe (Tabebuia, Bignoniaceae) logging in Amazonia: Sustainable management or catalyst for forest degradation?** *Biological Conservation* 2008, **141**(8):2071-2085.
18. Inagaki R, Ninomiya M, Tanaka K, Watanabe K, Koketsu M: **Synthesis and Cytotoxicity on Human Leukemia Cells of Furonaphthoquinones Isolated from Tabebuia Plants**. *Chemical & Pharmaceutical Bulletin* 2013, **61**(6):670-673.
19. Park BS, Kim JR, Lee SE, Kim KS, Takeoka GR, Ahn YJ, Kim JH: **Selective growth-inhibiting effects of compounds identified in Tabebuia impetiginosa inner bark on human intestinal bacteria**. *Journal of Agricultural and Food Chemistry* 2005, **53**(4):1152-1157.
20. Collevatti RG, Dornelas MC: **Clues to the evolution of genome size and chromosome number in Tabebuia alliance (Bignoniaceae)**. *Plant Systematics and Evolution* 2016, **302**(5):601-607.
21. Aronesty E: **Comparison of sequencing utility programs**. *The Open Bioinformatics Journal* 2013, **7**:1-8.
22. Langmead B, Trapnell C, Pop M, Salzberg SL: **Ultrafast and memory-efficient alignment of short DNA sequences to the human genome**. *Genome Biology* 2009, **10**(3).
23. Marcais G, Kingsford C: **A fast, lock-free approach for efficient parallel counting of occurrences of k-mers**. *Bioinformatics* 2011, **27**(6):764-770.
24. Vurture GW, Sedlazeck FJ, Nattestad M, Underwood CJ, Fang H, Gurtowski J, Schatz MC: **GenomeScope: fast reference-free genome profiling from short reads**. *Bioinformatics btx153* 2017.
25. Gnerre S, MacCallum I, Przybylski D, Ribeiro F, Burton J, Walker B, Sharpe T, Hall G, Shea T, Sykes S *et al*: **High-quality draft assemblies of mammalian genomes from massively parallel sequence data**. *Proceedings of the National Academy of Sciences* 2011, **108**(4):1513-1518.
26. Flutre T, Duprat E, Feuillet C, Quesneville H: **Considering Transposable Element Diversification in De Novo Annotation Approaches**. *Plos One* 2011, **6**(1).
27. Wicker T, Sabot F, Hua-Van A, Bennetzen JL, Capy P, Chalhoub B, Flavell A, Leroy P, Morgante M, Panaud O *et al*: **A unified classification system for eukaryotic transposable elements**. *Nature Reviews Genetics* 2007, **8**(12):973-982.
28. Hoede C, Arnoux S, Moisset M, Chaumier T, Inizan O, Jamilloux V, Quesneville H: **PASTEC: An Automatic Transposable Element Classification Tool**. *Plos One* 2014, **9**(5).
29. Smit AFA, Hubley R, Green P: **RepeatMasker Open-4.0 (2013-2015)**. 2015.
30. Bolger AM, Lohse M, Usadel B: **Trimmomatic: a flexible trimmer for Illumina sequence data**. *Bioinformatics* 2014, **30**(15):2114-2120.
31. Xie Y, Wu G, Tang J, Luo R, Patterson J, Liu S, Huang W, He G, Gu S, Li S *et al*: **SOAPdenovo-Trans: de novo transcriptome assembly with short RNA-Seq reads**. *Bioinformatics* 2014, **30**(12):1660-1666.

32. Grabherr MG, Haas BJ, Yassour M, Levin JZ, Thompson DA, Amit I, Adiconis X, Fan L, Raychowdhury R, Zeng QD *et al*: **Full-length transcriptome assembly from RNA-Seq data without a reference genome**. *Nature Biotechnology* 2011, **29**(7):644-U130.
33. Gilbert D: **EvidentialGene: mRNA Transcript Assembly Software**. *EvidentialGene : Evidence Directed Gene Construction for Eukaryotes* 2013.
34. Schmutz J, McClean P, Mamidi S, Wu A, Cannon S, Grimwood J, Jenkins J, Shu S, Song Q, Chavarro C *et al*: **A reference genome for common bean and genome-wide analysis of dual domestications**. *Nature Genetics* 2014, **46**(7):707-713.
35. Shu S, Goodstein DM, Hayes D, Mitros T, Rokhsar D: **JGI Plant Genomics Gene Annotation Pipeline**. *SciTech Connect* 2017.
36. Gish W, States D: **Identification of protein coding regions by database similarity search**. *Nature Genetics* 1993, **3**(3):266-272.
37. Slater GS, Birney E: **Automated generation of heuristics for biological sequence comparison**. *Bmc Bioinformatics* 2005, **6**.
38. **RepeatMasker Open-4.0. http://www.repeatmasker.org** [http://www.repeatmasker.org]
39. UniProt Consortium: **UniProt: a hub for protein information**. *Nucleic Acids Research* 2014, **43**(D1):D204-D212.
40. Salamov AA, Solovyev VV: **Ab initio gene finding in Drosophila genomic DNA**. *Genome Research* 2000, **10**(4):516-522.
41. Solovyev V, Kosarev P, Seledsov I, Vorobyev D: **Automatic annotation of eukaryotic genes, pseudogenes and promoters**. *Genome Biology* 2006, **7**.
42. Yeh RF, Lim LP, Burge CB: **Computational inference of homologous gene structures in the human genome**. *Genome Research* 2001, **11**(5):803-816.
43. Haas BJ, Delcher AL, Mount SM, Wortman JR, Smith RK, Hannick LI, Maiti R, Ronning CM, Rusch DB, Town CD *et al*: **Improving the Arabidopsis genome annotation using maximal transcript alignment assemblies**. *Nucleic Acids Research* 2003, **31**(19):5654-5666.
44. Jones P, Binns D, Chang HY, Fraser M, Li W, McAnulla C, McWilliam H, Maslen J, Mitchell A, Nuka G *et al*: **InterProScan 5: genome-scale protein function classification**. *Bioinformatics* 2014, **30**(9):1236-1240.
45. Braga AC, Collevatti RG: **Temporal variation in pollen dispersal and breeding structure in a bee-pollinated Neotropical tree**. *Heredity* 2011, **106**(6):911-919.
46. Sork VL, Fitz-Gibbon ST, Puiu D, Crepeau M, Gugger PF, Sherman R, Stevens K, Langley CH, Pellegrini M, Salzberg SL: **First Draft Assembly and Annotation of the Genome of a California Endemic Oak Quercus lobata Nee (Fagaceae)**. *G3-Genes Genomes Genetics* 2016, **6**(11):3485-3495.
47. Luo RB, Liu BH, Xie YL, Li ZY, Huang WH, Yuan JY, He GZ, Chen YX, Pan Q, Liu YJ *et al*: **SOAPdenovo2: an empirically improved memory-efficient short-read de novo assembler**. *Gigascience* 2012, **1**.
48. Zimin AV, Marcais G, Puiu D, Roberts M, Salzberg SL, Yorke JA: **The MaSuRCA genome assembler**. *Bioinformatics* 2013, **29**(21):2669-2677.
49. Kajitani R, Toshimoto K, Noguchi H, Toyoda A, Ogura Y, Okuno M, Yabana M, Harada M, Nagayasu E, Maruyama H *et al*: **Efficient de novo assembly of highly heterozygous genomes from whole-genome shotgun short reads**. *Genome Research* 2014, **24**(8):1384-1395.
50. Malinsky M, Simpson JT, Durbin R: **Trio-sga: facilitating de novo assembly of highly heterozygous genomes with parent-child trios**. *bioRxiv* 2016.

51. Boetzer M, Henkel CV, Jansen HJ, Butler D, Pirovano W: **Scaffolding pre-assembled contigs using SSPACE**. *Bioinformatics* 2011, **27**(4):578-579.

52. Nadalin F, Vezzi F, Policriti A: **GapFiller: a de novo assembly approach to fill the gap within paired reads**. *Bmc Bioinformatics* 2012, **13**.

53. Hunt M, Kikuchi T, Sanders M, Newbold C, Berriman M, Otto TD: **REAPR: a universal tool for genome assembly evaluation**. *Genome Biology* 2013, **14**(5):R47.

54. Ponstingl H, Ning ZM: **SMALT**. *2010 - 2015 Genome Research Ltd* 2016.

55. Kent WJ: **BLAT--the BLAST-like alignment tool**. *Genome research* 2002, **12**(4):656-664.

56. Stanke M, Keller O, Gunduz I, Hayes A, Waack S, Morgenstern B: **AUGUSTUS: ab initio prediction of alternative transcripts**. *Nucleic Acids Research* 2006, **34**:W435-W439.

57. Lam ET, Hastie A, Lin C, Ehrlich D, Das SK, Austin MD, Deshpande P, Cao H, Nagarajan N, Xiao M *et al*: **Genome mapping on nanochannel arrays for structural variation analysis and sequence assembly**. *Nature Biotechnology* 2012, **30**(8):771-776.

58. Ay F, Noble WS: **Analysis methods for studying the 3D architecture of the genome**. *Genome Biology* 2015, **16**.

59. Plomion C, Aury JM, Amselem J, Alaeitabar T, Barbe V, Belser C, Berges H, Bodenes C, Boudet N, Boury C *et al*: **Decoding the oak genome: public release of sequence data, assembly, annotation and publication strategies**. *Molecular ecology resources* 2016, **16**(1):254-265.

60. Wicker T, Sabot F, Hua-Van A, Bennetzen J, Capy P, Chalhoub B, Flavell A, Leroy P, Morgante M, Panaud O *et al*: **A unified classification system for eukaryotic transposable elements**. *Nature Reviews Genetics* 2007, **8**(12):973-982.

61. Morgante M, Hanafey M, Powell W: **Microsatellites are preferentially associated with nonrepetitive DNA in plant genomes**. *Nature Genetics* 2002, **30**(2):194-200.

62. Wang Q, Fang L, Chen J, Hu Y, Si Z, Wang S, Chang L, Guo W, Zhang T: **Genome-Wide Mining, Characterization, and Development of Microsatellite Markers in Gossypium Species**. *Scientific Reports* 2015, **5**(1).

63. Sonah H, Deshmukh R, Sharma A, Singh V, Gupta D, Gacche R, Rana J, Singh N, Sharma T: **Genome-Wide Distribution and Organization of Microsatellites in Plants: An Insight into Marker Development in Brachypodium**. *PLOS ONE* 2011, **6**(6):e21298.

64. Bernardi G: **Isochores and the evolutionary genomics of vertebrates**. *Gene* 2000, **241**(1):3-17.

65. Mizuno M, Kanehisa M: **Distribution profiles of GC content around the translation initiation site in different species**. *FEBS letters* 1994, **352**(1):7-10.

66. Amit M, Donyo M, Hollander D, Goren A, Kim E, Gelfman S, Lev-Maor G, Burstein D, Schwartz S, Postolsky B *et al*: **Differential GC content between exons and introns establishes distinct strategies of splice-site recognition**. *Cell reports* 2012, **1**(5):543-556.

67. Wendel JF, Greilhuber J, Dolezel J, Leitch IJ: **Plant Genome Diversity Volume 1 - Plant Genomes, their Residents, and their Evolutionary Dynamics**, vol. 1. Wien: Springer-Verlag; 2012.

68. JiaYan W, JingFa X, LingPing W, Jun Z, HongYan Y, ShuangXiu W, Zhang Z, Jun Y: **Systematic analysis of intron size and abundance parameters in diverse lineages**. *Science China Life Sciences* 2013, **56**(10):968-974.

69. Yu J, Yang Z, Kibukawa M, Paddock M, Passey D, Wong G: **Minimal Introns Are Not “Junk”**. *Genome Research* 2002, **12**(8):1185-1189.

70. Zhu J, He F, Wang D, Liu K, Huang D, Xiao J, Wu J, Hu S, Yu J: **A Novel Role for Minimal Introns: Routing mRNAs to the Cytosol**. *PLOS ONE* 2010, **5**(4):e10144.

71. Simao FA, Waterhouse RM, Ioannidis P, Kriventseva EV, Zdobnov EM: **BUSCO: assessing genome assembly and annotation completeness with single-copy orthologs**. *Bioinformatics* 2015, **31**(19):3210-3212.

72. Veeckman E, Ruttink T, Vandepoele K: **Are We There Yet? Reliably Estimating the Completeness of Plant Genome Sequences**. *Plant Cell* 2016, **28**(8):1759-1768.

73. Ye J, Fang L, Zheng H, Zhang Y, Chen J, Zhang Z, Wang J, Li S, Li R, Bolund L *et al*: **WEGO: a web tool for plotting GO annotations**. *Nucleic Acids Research* 2006, **34**(Web Server issue):W293-W297.

74. Park B-S, Lee H-K, Lee S-E, Piao X-L, Takeoka G, Wong R, Ahn Y-J, Kim J-H: **Antibacterial activity of Tabebuia impetiginosa Martius ex DC (Taheebo) against Helicobacter pylori**. *Journal of Ethnopharmacology* 2006, **105**(1-2):255-262.

75. Gómez Castellanos R, Prieto J, Heinrich M: **Red Lapacho (Tabebuia impetiginosa)—A global ethnopharmacological commodity?** *Journal of Ethnopharmacology* 2009, **121**(1):1-13.

76. Byeon S, Chung J, Lee Y, Kim B, Kim K, Cho J: **In vitro and in vivo anti-inflammatory effects of taheebo, a water extract from the inner bark of Tabebuia avellanedae**. *Journal of Ethnopharmacology* 2008, **119**(1):145-152.

77. Koyama J, Morita I, Tagahara K, Hirai K-I: **Cyclopentene dialdehydes from Tabebuia impetiginosa**. *Phytochemistry* 2000, **53**(8):869-872.

78. Hussain H, Krohn K, Ahmad VU, Miana GA, Green IR: **Lapachol: An overview**. *Arkivoc* 2007, **2007**(2):145.

79. Widhalm J, Rhodes D: **Biosynthesis and molecular actions of specialized 1,4-naphthoquinone natural products produced by horticultural plants**. *Horticulture Research* 2016, **3**:16046.

80. Romagnoli M, Segoloni E, Luna M, Margaritelli A, Gatti M, Santamaria U, Vinciguerra V: **Wood colour in Lapacho (Tabebuia serratifolia): chemical composition and industrial implications**. *Wood Science and Technology* 2013, **47**(4):701-716.

Table 1. *Handroanthus impetiginosus* genome assembly statistics.

Scaffold sequences	Allpaths-LG	Allpaths-LG/ Sspace/GapClose	Allpaths-LG/Sspace/ GapClose/Reapr
**Number**	57,815	16,090	13,206
**Total size, without gaps (bp)**	469,049,393	565,959,143	476,867,120
**Total size, with gaps (bp)**	614,626,609	586,542,612	503,314,177
**Number > 10 Kbp**	10,029	8,602	8,348
**Number > 20 Kbp**	6,920	6,791	6,647
**Number > 100 Kbp**	1,100	1,709	1,304
**Number > 1 Mbp**	2	0	0
**Longest sequence (bp)**	1,844,569	979,053	558,523
**Average size (bp)**	10,631	36,454	38,112
**N50 length (bp)**	57,726	97,266	80,946
**L50 count**	2,595	1,792	1,906
**GC %**	33.63	33.57	33.62

Table 2. *Handroanthus impetiginosus* gene prediction statistics with respect to the number, length and base composition of genes, transcripts, exons and introns.

	Genes	Transcripts	Exons	Introns
**Number**	31,688	35,479	154,209	122,521
**Average number/gene**	-	1.12	4.87	3.87
**Average length**	3,129	3,342	285	445
**N50 length**	4,421	4,643	477	839
**%GC**	38.38	38.22	42.60	32.83
**%N**	0.43	0.43	0.00	0.29

Table 3. The distribution of the minimal introns (53–125 bp) and the minimal-intron-containing genes – as the number of genes with at least one minimal intron – from selected plant species in comparison to the H. impetiginosus genome assembly.

**Species**	Genome size (Mbp)	Number of intron (bp)	Mean intron length (bp)	Minimal intron (%)	Gene (%)
A. thaliana **(Rosids)**	120	118,037	164	72.29	57.08
E. guttata **(Asterids)**	312	117,507	290	47.75	57.63
P. trichocarpa **(Rosids)**	423	166,809	380	36.96	53.41
E. grandis **(Rosids)**	691	137,329	425	33.49	48.38
S. indicum **(Asterids)**	354	101,313	439	38.14	49.76
H. impetiginosus **(Asterids)**	557	122,521	445	34.36	49.78
S. lycopersicum **(Asterids)**	900	125,750	543	36.09	47.78

Figure Legends

Figure 1. Depth of coverage analysis. (A) Histograms of k-mer frequencies in the filtered read data for k = 25 (red) and GenomeScope modeling equation on *H. impetiginosus* (blue). The x-axis shows the number of times a k-mer occurred (coverage). The vertical dashed dark blue lines correspond to the mean coverage values for unique heterozygous k-mers (left peak) and unique homozygous k-mers (right peak). (B) Density plot of read depth based on mapping all short fragment reads back to the assembled scaffolds (red). Left peak (at depth = 34x) corresponds to regions where the assembler created two distinct scaffolds from divergent putative haplotypes. The right peak (at depth = 67x) contains scaffolds from regions where the genome is less variable, allowing the assembler to construct a single contig combining homologue sequences. Histograms of Poisson modeling for read depth in the assembly (green, lambda = 34; blue, lambda = 67) are shown.

Figure 2. Depth of coverage analysis for the haplotype-reduced assembly. (A) Density plot of read depth based on mapping all short fragment reads back to the haplotype-reduced assembled sequences after identification and removal of redundant sequences due the structural heterozygosity in the genome. (B) Density plot for average sequencing coverage per-scaffold on the final assembly. The observed number of scaffolds in the final haplotype-reduced assembly and the respective read coverage (blue line) is shown in comparison to a Poisson process approximation (red line) with lambda = 63x, the observed average sequencing coverage in the useful read data.

Figure 3. Repeat content of the *H. impetiginosus* genome assembly. (A) The density of interspersed and tandem repeat as percent of the assembly. The size of the circles represents the number of copies in the assembly for each family of repeats; (B) Distribution of sizes of the consensus sequences for repeat families identified using *de novo* and homology methods for repeat characterization.

Figure 4. Transcriptome quality assessment (A) similarity search of *H. impetiginosus* putative peptides against source database of plant protein sequences using BLASTP algorithm. Transcript count means the number of peptides of *H. impetiginosus* with best hit against the source database using bit-score and grouping results by taxon name. Transcript score corresponds to the average bit-score overall hits for each group using the best hit; (B) Completeness of the

expected gene space of the genome assembly, estimated with BUSCO. The estimates were compared with the well-curated *P. trichocarpa* v. 3 annotation.

Figure 5. Genes of the biosynthetic pathway of specialized quinoids. O-succinylbenzoate (OSB) pathway depicting the number of *H. impetiginosus* (Himp) annotated genes for the known enzymes that lead to the biosynthesis of the naphthoquinones, including lapachol. For comparison, it also shows the numbers of genes for the closely related *Mimulus guttatus* (Mgut), *Solanum lycopersicum* (Slyc), for the model *Arabidopsis thaliana* (Ath), and for the tree species *Eucalyptus grandis* (Egr) and *Populus trichocarpa* (Potri). The pathway was modified from [79].

Supplementary material

Fig. S1. The *Handroanthus impetiginosus* (Mart. ex DC.) Mattos (syn. *Tabebuia impetiginosa*, Bignoniaceae), tree UFG-1 whose genome was sequenced.

Fig. S2. Flow cytometry results of the sequenced tree UFG-1 of *H. impetiginosus*. Flow cytometry estimate of the nuclear DNA content was carried out using young leaf tissue on a BD Accuri™ C6 Plus personal flow cytometer. *Pisum sativum* (genome size 9.09 pg/2C or ~4380 Mb/1C) was used as standard for comparison (M2). The estimate of nuclear DNA content for *H. impetiginosus* (M1) averaged over 10 readings was 1.155 pg/2C or 557.3 ± 39 Mb/1C.

Figure S3. Overview of the analytical pipeline with the bioinformatics steps and tools employed for genome (black arrows) and transcriptome assembly (red arrows), and for gene prediction and annotation (blue arrows). Bioinformatics programs are indicated in italic, blue, and the main file formats in red. The input sequences are highlighted in yellow boxes and the main products in green.

Table S1. Summary of the sequence data generated for the genome assembly of *Handroanthus impetiginosus* based on the ALLPATHS-LG algorithm.

Figure S4. Distribution and characterization of simple sequence repeats in Handroanthus impetiginosus genome (A) Histogram of different motifs ranging from 1 to 6 bp (B) Distribution of the simple sequence repeats length detected in the genome assembly.

Figure S5. A comparison of the gene features parameters, such as number and length, between *H. impetiginosus* and the other selected dicot plant across distinct lineages of Rosids (*A. thaliana* and *P. trichocarpa*) and Asterids (*E. guttata* and *S. lycopersicum*). Frequency histograms are shown according to the whole-genome gene content annotation for (A) the complete predicted gene structure (B) exons and (C) introns. Dashed vertical lines are the average lengths for the gene features.

Figure S6. Histograms for Gene Ontology term annotations in the *H. impetiginosus* genome assembly. Terms for the Biological Process ontology were summarized with WEGO by the second tree level setting. The Pearson Chi-Square test was applied to indicate significant relationships between *H. impetiginosus* and the model tree *P. trichocarpa* regarding the number of genes (at $\alpha \geq 5\%$). (A) Terms displaying a remarkable relationship between the two datasets; (B) terms with a significant difference between the two datasets.

Figure 1

Click here to download Figure
Figure_Main_1.pdf

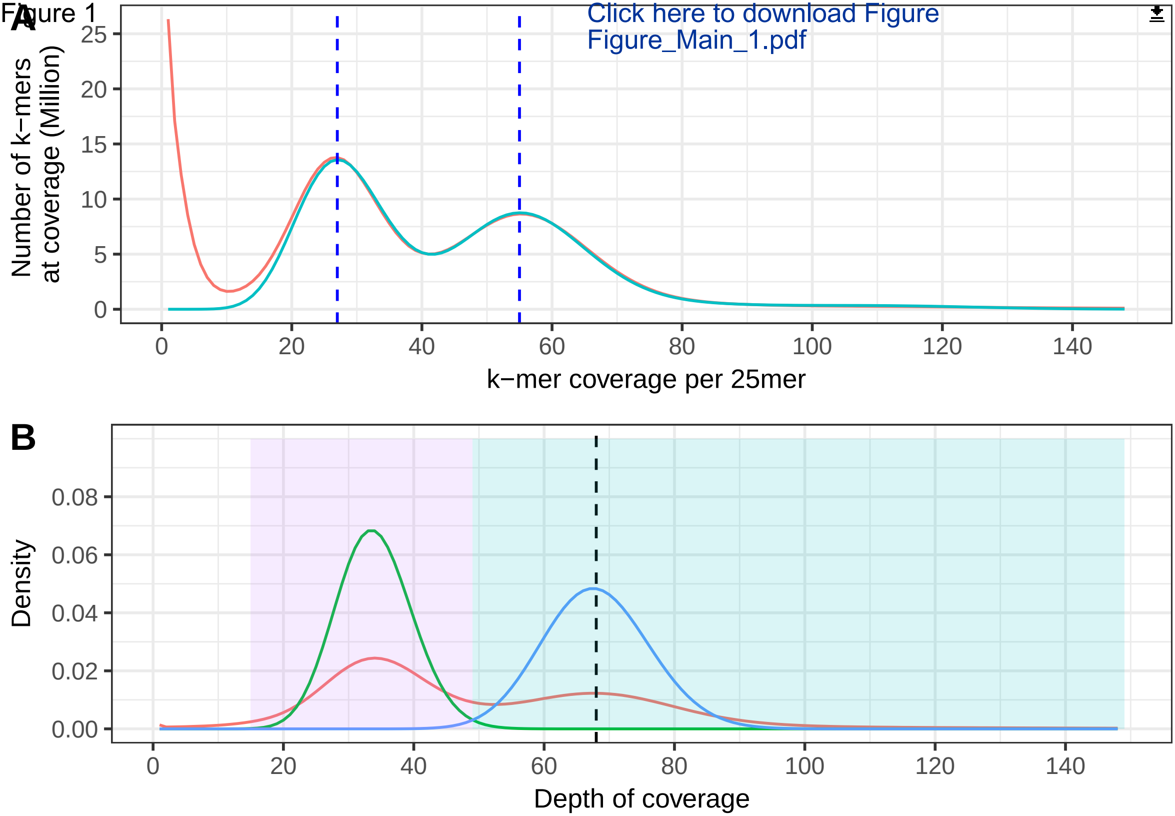

Figure 2

Click here to download Figure Figure_Main_2.pdf

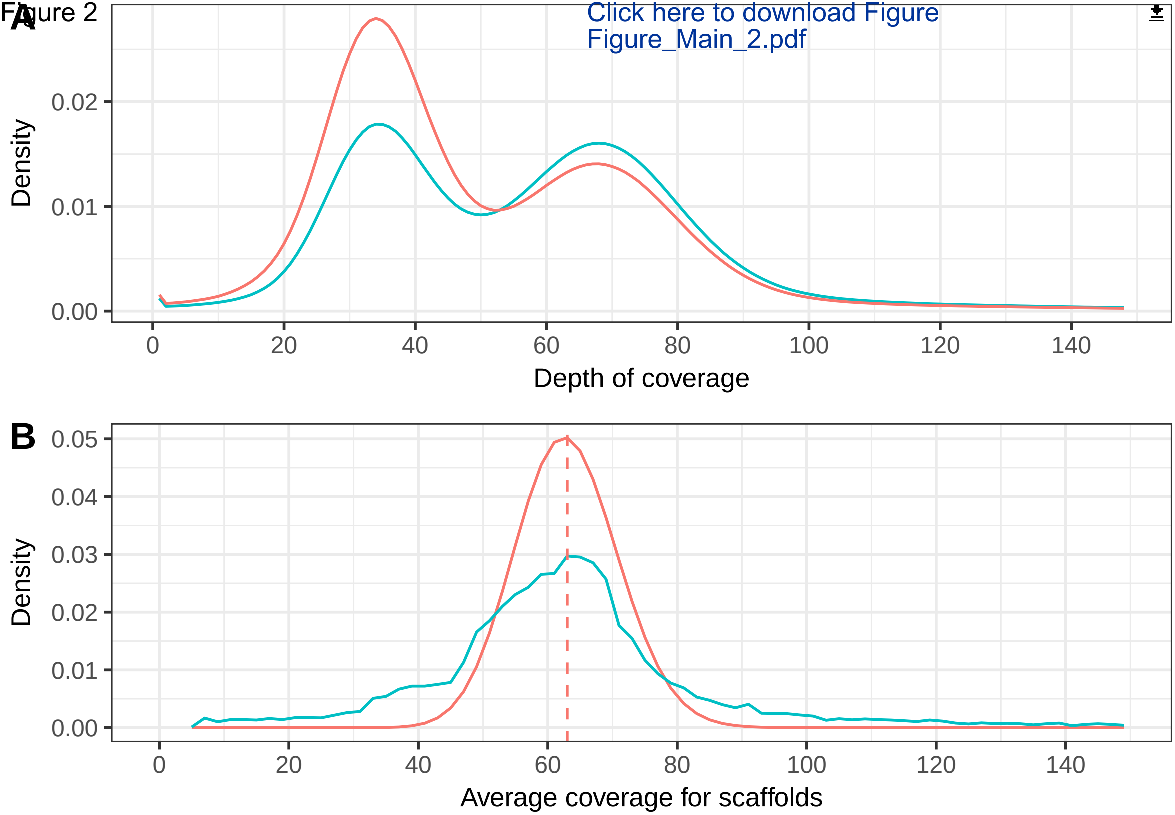

Figure 3

Click here to download Figure Figure_Main_3.pdf

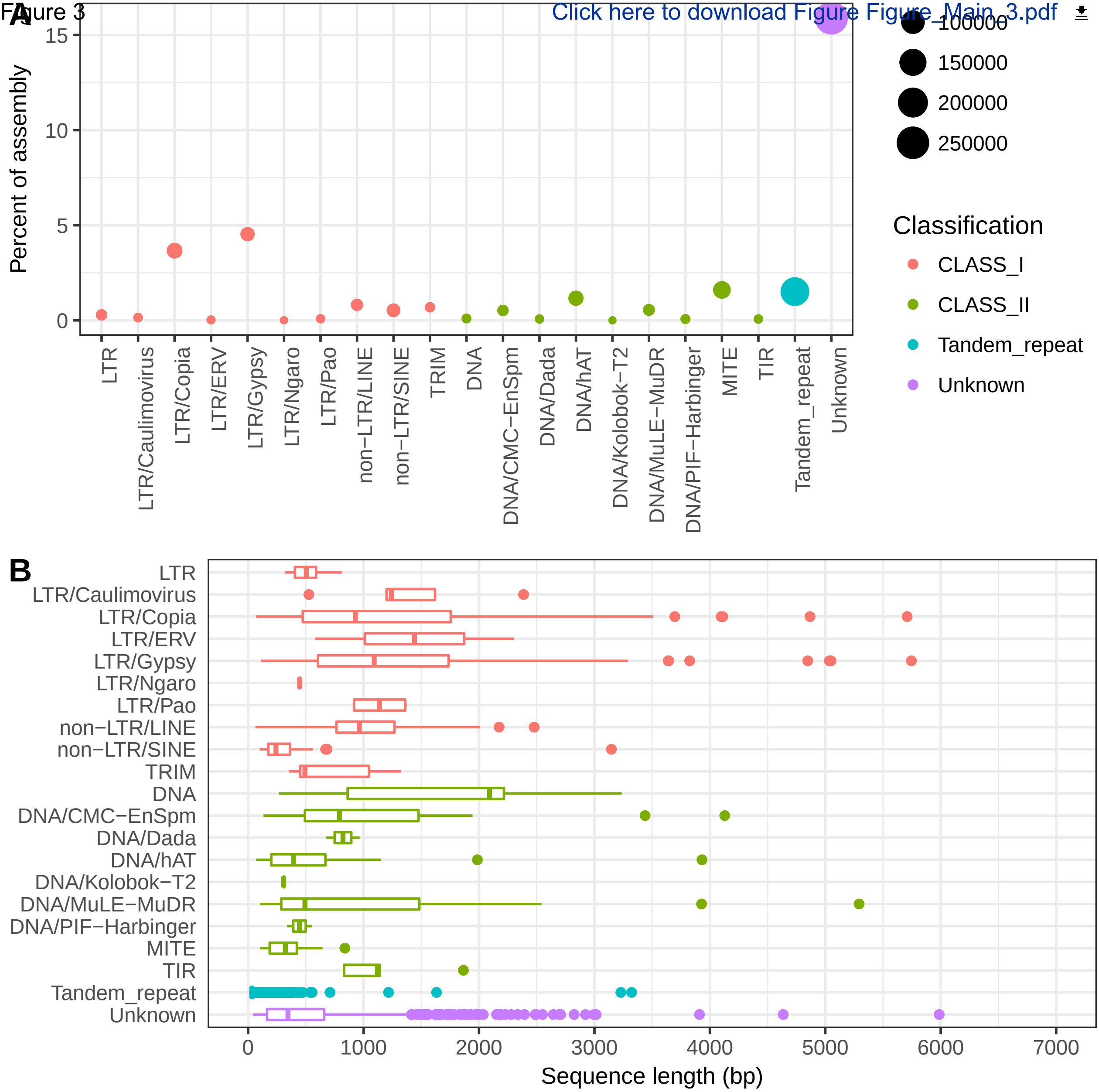

Figure 4

Click here to download Figure Figure_Main_4.pdf

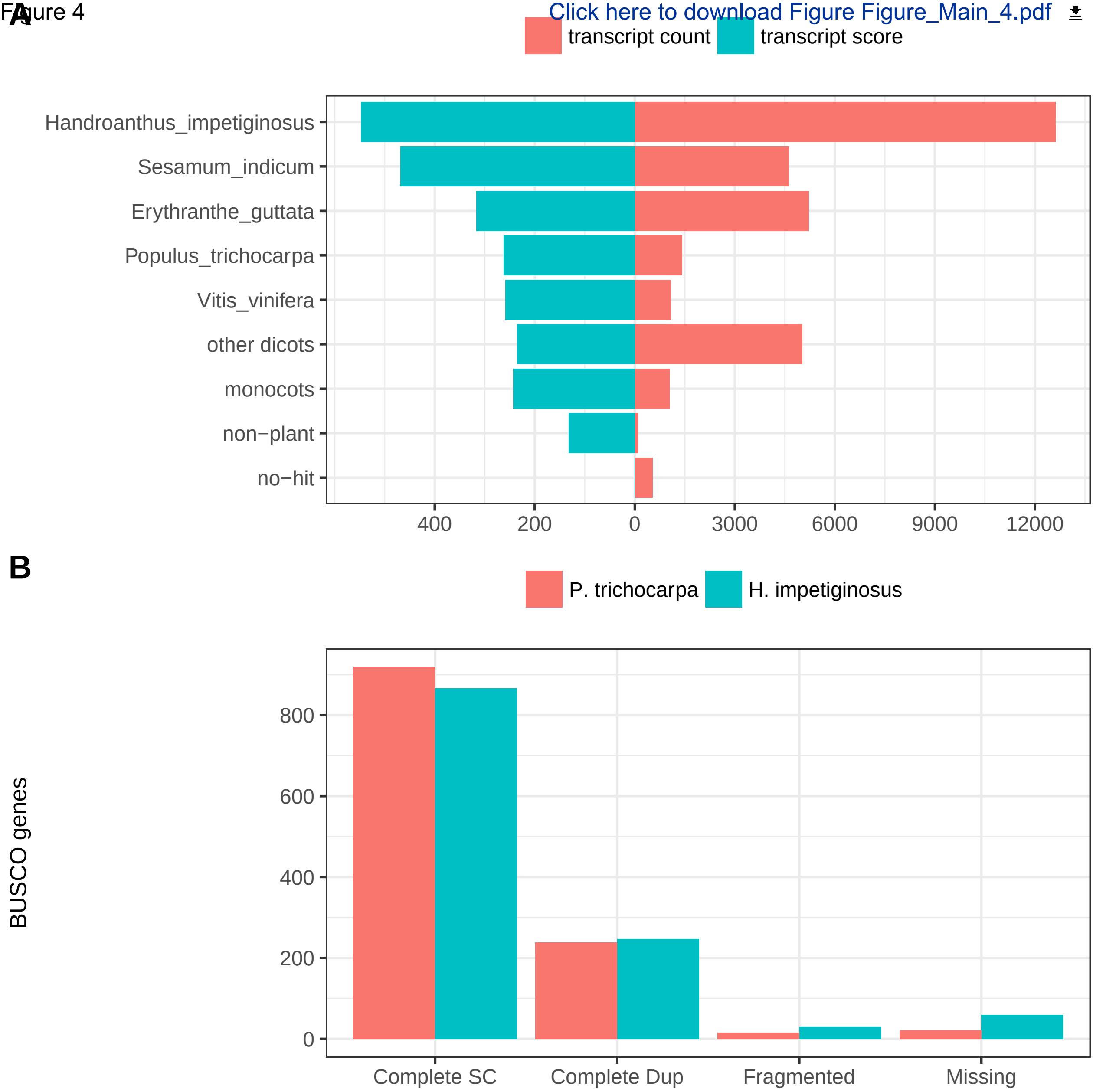

Figure 5

Click here to download Figure Figure_Main_5.pdf

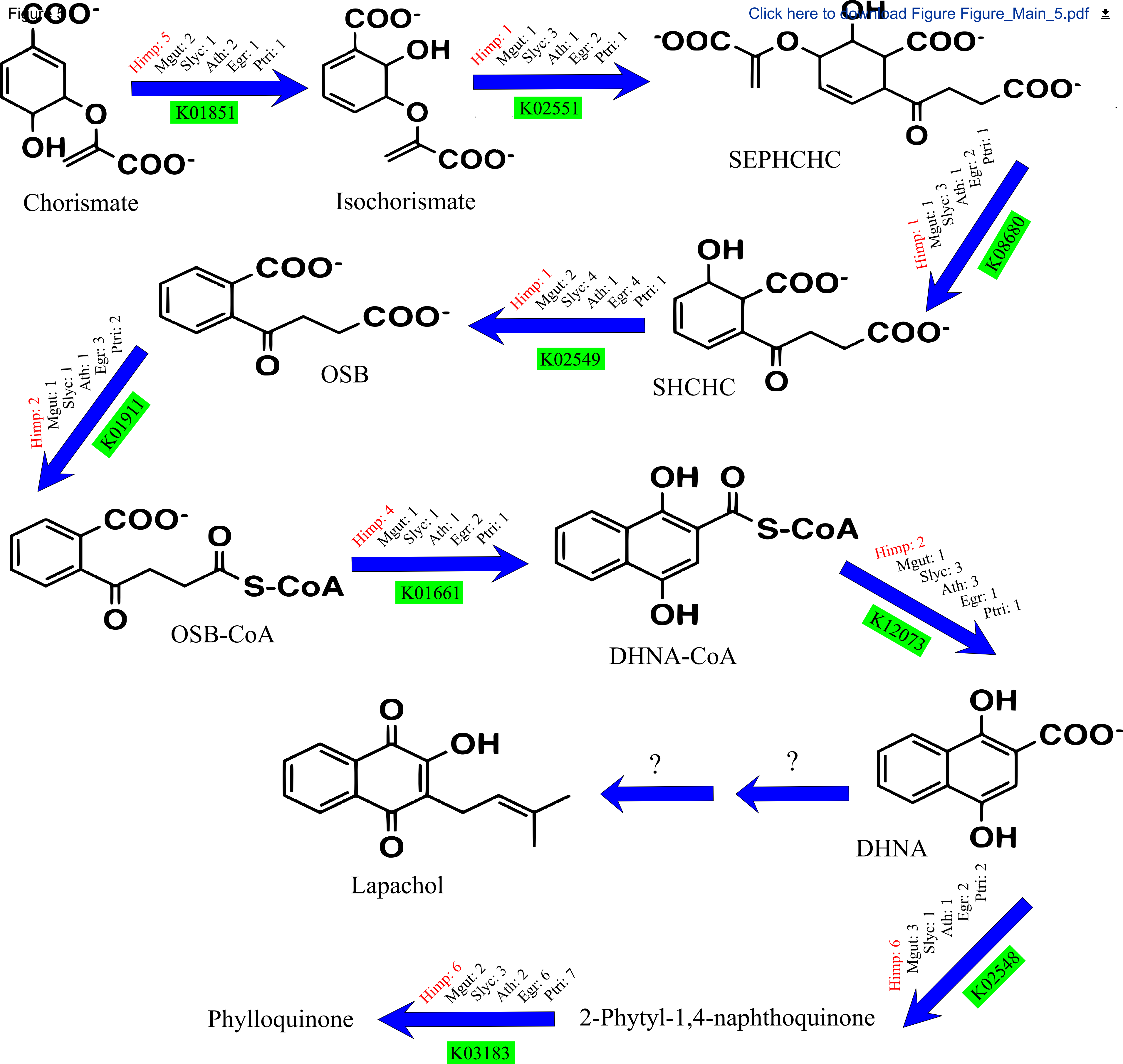

Click here to access/download
Supplementary Material
Supp_Material_H.impetiginosus_genome.docx
