# Supplementary material for: Genome assembly of the Pink Ipê (Handroanthus impetiginosus, Bignoniaceae), a highly valued, ecologically keystone Neotropical timber forest tree
Source: Gigascience. 2017 Dec 13;7(1):gix125. doi: 10.1093/gigascience/gix125 (PMC5905499; doi:10.1093/gigascience/gix125)
Supplement: GIGA-D-17-00159_Revision_2.pdf [file gix125_giga-d-17-00159_revision_2.pdf]

GigaScience

Genome assembly of the pink Ipê (Handroanthus impetiginosus, Bignoniaceae), a highly-valued ecologically keystone Neotropical timber forest tree

--Manuscript Draft--

**Manuscript Number:**	GIGA-D-17-00159R2
**Full Title:**	Genome assembly of the pink Ipê (Handroanthus impetiginosus, Bignoniaceae), a highly-valued ecologically keystone Neotropical timber forest tree
**Article Type:**	Data Note
**Funding Information:**	CNPq (471366/2007-2) — Professor Rosane Garcia Collevatti<br>CNPq (457406/2012-7) — Professor Rosane Garcia Collevatti<br>CNPq (476709/2012-1) — Dr Evandro Novaes<br>FAP-DF (193.000.570/2009) — Dr Dario Grattapaglia
**Abstract:**	Background: Handroanthus impetiginosus (Mart. ex DC.) Mattos is a keystone Neotropical hardwood tree widely distributed in seasonally dry tropical forests of South and Mesoamerica. Regarded as the "new mahogany", it is the second most expensive timber, the most logged species in Brazil, and currently under significant illegal trading pressure. The plant produces large amounts of quinoids, specialized metabolites with documented antitumorous and antibiotic effects. The development of genomic resources is needed to better understand and conserve the diversity of the species, to empower forensic identification of the origin of timber and to identify genes for important metabolic compounds.<br><br>Findings: The genome assembly covers 503.7Mb (N50=81,316 bp), 90.4% of the 557 Mbp genome, with 13,206 scaffolds. A repeat database with 1,508 sequences was developed allowing masking ~31% of the assembly. Depth of coverage indicated that consensus determination adequately removed haplotypes assembled separately due to the extensive heterozygosity of the species. Automatic gene prediction provided 31,688 structures and 35,479 mRNA transcripts, while external evidence supported a well-curated set of 28,603 high-confidence models (90% of total). Finally, we used the genomic sequence and the comprehensive gene content annotation to identify genes related to the production of specialized metabolites.<br><br>Conclusions: This genome assembly is the first well-curated resource for a Neotropical forest tree and the first one for a member of the Bignoniaceae family, opening exceptional opportunities to empower molecular, phytochemical and breeding studies. This work should inspire the development of similar genomic resources for the largely neglected forest trees of the mega-diverse tropical biomes.
**Corresponding Author:**	Rosane Garcia Collevatti, PhD<br>Universidade Federal de Goias<br>Goiania, GO BRAZIL
**Corresponding Author Secondary Information:**	
**Corresponding Author's Institution:**	Universidade Federal de Goias
**Corresponding Author's Secondary Institution:**	
**First Author:**	orzenil.silva@embrapa.br B Silva-Junior, PhD
**First Author Secondary Information:**	
**Order of Authors:**	orzenil.silva@embrapa.br B Silva-Junior, PhD<br>Dario Grattapaglia, PhD

Powered by Editorial Manager® and ProduXion Manager® from Aries Systems Corporation

	Evandro Novaes, PhD
	Rosane Garcia Collevatti, PhD
**Order of Authors Secondary Information:**	
**Response to Reviewers:**	Dr Hans Zauner<br>Assistant Editor<br>GigaScience<br><br>We double checked the manuscript and perform minor corrections.<br>We also moved Figure S1 (ipê photo) to the main-text (it is now Figure 1) and change figure and supplemental material numbers.<br><br>Hope our changes are satisfactory.<br><br>All the best<br>Rosane Collevatti
**Additional Information:**	
**Question**	**Response**
Are you submitting this manuscript to a special series or article collection?	No
**Experimental design and statistics**<br><br>Full details of the experimental design and statistical methods used should be given in the Methods section, as detailed in our Minimum Standards Reporting Checklist. Information essential to interpreting the data presented should be made available in the figure legends.<br><br>Have you included all the information requested in your manuscript?	Yes
**Resources**<br><br>A description of all resources used, including antibodies, cell lines, animals and software tools, with enough information to allow them to be uniquely identified, should be included in the Methods section. Authors are strongly encouraged to cite Research Resource Identifiers (RRIDs) for antibodies, model organisms and tools, where possible.<br><br>Have you included the information requested as detailed in our Minimum Standards Reporting Checklist?	Yes
**Availability of data and materials**<br><br>All datasets and code on which the conclusions of the paper rely must be	Yes

Powered by Editorial Manager® and ProduXion Manager® from Aries Systems Corporation

either included in your submission or deposited in publicly available repositories (where available and ethically appropriate), referencing such data using a unique identifier in the references and in the “Availability of Data and Materials” section of your manuscript.<br><br>Have you have met the above requirement as detailed in our Minimum Standards Reporting Checklist?	

Powered by Editorial Manager® and ProduXion Manager® from Aries Systems Corporation

Manuscript

Click here to download Manuscript Himpetiginosus_271117_ED_R2.docx

Genome assembly of the Pink Ipê (*Handroanthus impetiginosus*, *Bignoniaceae*), a highly-valued ecologically keystone Neotropical timber forest tree

Orzenil Bonfim da Silva-Junior[1,2], Dario Grattapaglia[1,2], Evandro Novaes[3], Rosane G. Collevatti[4*]

[1]*EMBRAPA Recursos Genéticos e Biotecnologia, EPqB, Brasília, DF. 70770-910. Brazil.*
[2]*Programa de Ciências Genômicas e Biotecnologia – Universidade Católica de Brasília, SGAN 916 Modulo B, Brasilia, DF 70790-160. Brazil*
[3]*Escola de Agronomia, Universidade Federal de Goiás, CP 131. Goiânia, GO. 74001-970. Brazil.*
[4]*Laboratório de Genética & Biodiversidade, Instituto de Ciências Biológicas, Universidade Federal de Goiás. Goiânia, GO. 74001-970. Brazil.*

***Corresponding author:** Rosane Garcia Collevatti, Instituto de Ciências Biológicas, Universidade Federal de Goiás, 74001-970, Goiânia, GO, Brasil.

E-mail: rosanegc68@hotmail.com. Phone: +55 62 3521-1729.

33

Abstract

Background: *Handroanthus impetiginosus* (Mart. ex DC.) Mattos is a keystone Neotropical hardwood tree widely distributed in seasonally dry tropical forests of South and Mesoamerica. Regarded as the "new mahogany", it is the second most expensive timber, the most logged species in Brazil, and currently under significant illegal trading pressure. The plant produces large amounts of quinoids, specialized metabolites with documented antitumorous and antibiotic effects. The development of genomic resources is needed to better understand and conserve the diversity of the species, to empower forensic identification of the origin of timber and to identify genes for important metabolic compounds.

Findings: The genome assembly covers 503.7Mb (N50=81,316 bp), 90.4% of the 557 Mbp genome, with 13,206 scaffolds. A repeat database with 1,508 sequences was developed allowing masking ~31% of the assembly. Depth of coverage indicated that consensus determination adequately removed haplotypes assembled separately due to the extensive heterozygosity of the species. Automatic gene prediction provided 31,688 structures and 35,479 mRNA transcripts, while external evidence supported a well-curated set of 28,603 high-confidence models (90% of total). Finally, we used the genomic sequence and the comprehensive gene content annotation to identify genes related to the production of specialized metabolites.

Conclusions: This genome assembly is the first well-curated resource for a Neotropical forest tree and the first one for a member of the *Bignoniaceae* family, opening exceptional opportunities to empower molecular, phytochemical and breeding studies. This work should inspire the development of similar genomic resources for the largely neglected forest trees of the mega-diverse tropical biomes.

Keywords: heterozygous genome, RNA-seq, transposable elements, quinoids, *Bignoniaceae*

DATA DESCRIPTION

Context. The generation of plant genome assemblies is a key driver to develop powerful genomic resources that allow gaining detailed insights into the evolutionary history of species while enabling breeding and conservation efforts [1, 2]. Such advances took place first in model plant species [3] followed by the mainstream [4] and minor crops [5], and some major forest trees [6-9]. Genome sequences have also driven important advances in the description and understanding of essential plant metabolic processes that underlie survival across distinct lineages. Research on the functional roles of specialized metabolites, many of them phylogenetically restricted [10], has recently addressed the gap in the species-specific knowledge of specialized plant metabolism by sequencing the genome of key medicinal plants [11, 12]. Innovation in this field has relied on a combination of high-throughput genomics, including massive parallel sequencing and arrays with animal and clinical studies to elucidate the mechanisms of target compounds as adjuvant therapies, to demonstrate the necessary formulations for its biological effects and to determine which substances are beneficial or toxic. Apart from recent reports of shallow transcriptome characterization using 454 pyrosequencing [13] and a low-coverage (11X) fragmented genome assembly [14], essentially no well-curated genome assembly and gene content annotation exist for Neotropical forest trees, despite their recognized value by indigenous communities for the healing properties of their special metabolites, increasingly exploited by large pharmaceutical corporations [15, 16]. An example of such tree is the species *Handroanthus impetiginosus* (Mart. ex DC.) Mattos (syn. *Tabebuia impetiginosa*, Bignoniaceae), popularly known as Pink Ipê, Lapacho or Pau d'arco, a source of both high value timber and traditional medicine.

Species of *Handroanthus* and *Tabebuia* have virtually no genomic tools and resources, beyond a handful of 21 microsatellites [17] with their known caveats for more sophisticated genetic analyses in the areas of population genomics and evolution [18]. Whole-genome sequencing has now become accessible to a point that efforts to develop improved genomic resources for such species are possible and warranted. We built a preliminary assembly of the nuclear genome of a single individual of *Handroanthus impetiginosus* based on short-reads and longer mate-pair DNA sequence data to provide the necessary framework for the development of genomic resources to support multiple genomic and genetic analyses of this keystone Neotropical hardwood tree

regarded as the "new mahogany". It is the second most expensive timber and the most logged species in Brazil [19], exported largely to North America for residential decking and currently under significant illegal trading pressure. Additionally, the tree produces large amounts of natural products such as those of quinoid systems (1,4-anthraquinones, 1,4-naphthoquinones, and 1,2-furanonaphthoquinones), specialized metabolites with promising antitumorous, anti-inflammatory and antibiotic effects [20, 21]. The high pressure of logging and illegal trading on this species with a notable ecological keystone status urges conservation efforts of existing populations.

METHODS

Sample collection and sequencing. DNA of a single adult tree of *H. impetiginosus* (UFG-1) (Figure 1) was extracted using Qiagen DNeasy Plant Mini kit (Qiagen, DK). Flow cytometry was used to check the genome size of tree UFG-1 indicating a genome size of (557 ±39) Mb /1C (Figure S1) consistent with published estimates [22]. Total RNA from shoots of five seedlings and from the differentiating xylem of the adult tree (UFG-1) was extracted using Qiagen RNeasy Plant Mini kit (Qiagen, DK) and pooled for RNA sequencing. DNA and RNA sequencing was performed at the High-Throughput Sequencing and Genotyping Center of the University of Illinois Urbana-Champaign, USA. The following libraries were generated for sequencing: (1) two shotgun genomic libraries of short fragments (300bp and 600bp) from tree UFG-1 (2) one shotgun library from combined pools of five RNA samples tagged with a single index sequence. Paired-end sequencing, 2x150 nt, was performed in two lanes of an Illumina HiSeq 2500 instrument (Illumina, CA, USA). Three additional mate-pair libraries (fragment lengths of 4kb to 5.5kb, 8kb to 10kb and 15kb to 20kb) for UFG-1 were also sequenced in two lanes of an Illumina HiSeq 2000 instrument (2x101 bp). This long-range sequence resource was used to generate the final genome assembly for annotation. A complete overview of the genome assembly and annotation pipeline is provided (Figure S2).

[Insert Figure 1 here]

Genome assembly using short paired-end and mate pair sequencing data. Short reads and mate-pair reads were stripped of sequencing adapters using *Fastq-mcf* [23]. Reads that mapped

to a database containing mitochondrial and chloroplast genomes of plants with *Bowtie1* [24] (option –v 3 –a –m 1) were discarded. Mate-pair reads were inspected using a *Perl* script (TrimAdaptor.pl), and sequences that did not contain the circularization adaptor were discarded. By using the filtered short reads, Jellyfish2 (Jellyfish, RRID:SCR_005491) [25] and GenomeScope [26] were applied to obtain estimates of the *H. impetiginosus* genome size, repeat fraction and heterozygosity prior to the assembly. *ALLPATHS-LG (ALLPATHS-LG, RRID:SCR_010742)* [27] was used for *de novo* assembly of the sequence data from both paired-end and mate-pair data, with default options, in a stepwise strategy for error correction of reads, handling of repetitive sequences and use of mate-pair libraries.

Transposable elements and repetitive DNA. Repetitive elements were detected and annotated on the genome assembly with the RepeatModeler *de novo* repeat family identification and modeling package (RepeatModeler, RRID:SCR_015027) [28]. Using RECON, RepeatScout and Tandem Repeat Finder, repetitive sequences were detected in the scaffolds longer than 10 kb using a combination of similarity-based and *de novo* approaches. The TE sequences were evaluated using modeling capabilities of the RepeatModeler program, with default settings,to compare the TE library against the entire assembled sequences and to refine and classify consensus models of putative interspersed repeats. A complementary analysis intended to augment the number of TE sequences classified according to current criteria [29] was performed using the PASTEC program [30]. RepeatMasker Open-4.0 (RepeatMasker, RRID:SCR_012954) [31] was used with the sequences from the *de novo* repetitive element library to annotate the interspersed repeats and to detect simple sequence repeats (SSRs) on the genome assembly.

Protein-coding genes annotation. Protein-coding genes annotation was performed with a pipeline that combines RNA-seq assembled transcript and protein alignments to the reference with *de novo* predictions methods (Figure S2). RNA-Seq reads were screened for the presence of adapters, which were removed using *Fastq-mcf* [23]. *Trimmomatic (Trimmomatic, RRID:SCR_011848)* [32] was used to (1) remove low quality, no base called segments (N's) from sequencing reads; (2) scan the read with a 4-base sliding window, cutting when the average quality per base dropped below 15; and (3) remove reads shorter than 32 bp after trimming. Trimmed reads mapped to mitochondrial, chloroplast and ribosomal sequences from plants with *Bowtie1* [24] (options –v 3 –a –m 1) were also removed. Transcript *de novo* assemblies were

performed using *SOAP-Transdenovo* [33] and *Trinity de-novo* [34] from the processed reads. The assemblies were concatenated and used as input to *EvidentialGene* [35], a comprehensive transcriptome pipeline to identify likely complete coding regions and their proteins in the final, combined, transcriptome assembly. Gene modeling was carried out using standard procedures and tools described, for instance, in [36]. In summary, a genome-guided transcriptome assembly of *H. impetiginosus* was performed with the JGI PERTRAN RNA-seq Read Assembler pipeline [37] using both the RNA-Seq trimmed reads and sequences from the *de novo* transcript assembly. Loci were identified by the assembled transcript alignments using BLASTX [38] and EXONERATE [39] alignments of peptide sequences to the repeat-soft-masked genome using RepeatMasker [40], based on a transposon database developed as part of this genome assembly annotation. Known peptide sequences included manually curated data sets for plant species available from UniProtKB/Swiss-Prot [41] and sequences available from Phytozome [1] version 11 for *Arabidopsis thaliana*, *Oryza sativa*, *Erythranthe guttata*, *Solanum lycopersicum*, *Solanum tuberosum*, *Populus trichocarpa* and *Vitis vinifera*. Gene structure were predicted by homology-based predictors, FGENESH++, FGENESH_EST [42, 43] and GenomeScan [44]. Gene predictions were improved by PASA (PASA, RRID:SCR_014656) [45], including adding UTRs, correcting splicing and adding alternative transcripts. PASA-improved gene model peptides were subjected to peptide homology analysis with the above-mentioned proteomes to obtain Cscore values and peptide coverage. Cscore is the ratio of the peptide BLASTP score to the mutual best hit BLASTP score, and peptide coverage is the highest percentage of peptide aligned to the best homolog. A transcript was selected if its Cscore value was greater than or equal to 0.5 and its peptide coverage was greater than or equal to 0.5 or if it had transcript coverage but the proportion of its coding sequence overlapping repeats was less than 20%. For gene models where greater than 20% of the coding sequence overlapped with repeats, the Cscore value was required to be at least 0.9 and homology coverage was required to be at least 70% to be selected. Selected gene models were then subjected to classification analysis using *InterProScan 5 (InterProScan, RRID:SCR_005829)* [46] for PFAM domains, PANTHER, Enzyme Comissioned Number (EC) and KEGG categories. Gene ontology annotation was obtained, where possible, from Interpro2GO and EC2GO mappings.

DATA VALIDATION AND QUALITY CONTROL

Global properties of the H. impetiginosus tree genome from the unassembled reads. Sequencing of the *H. impetiginosus* tree genome generated c. 599 million reads, comprising 73 Gbp of sequence data. This represents nearly 132× the expected sequence coverage. After removal of adaptors, followed by standard error correction and trimming with ALLPATHS-LG, with default options, c. 46 Gbp of data was found useful for the assembly process, yielding sequencing coverage of 82x (63x from the fragments libraries and 19x from the mate pair libraries). The estimated physical coverage was 400x based on the observed fragment size distributions (Table S1). ALLPATHS-LG k-mer spectrum frequency analysis (at K=25) on useful reads, error corrected reads, estimated a haploid genome size of 540,968,531 bp, a repeat fraction of 38.0%, and a SNP rate of 1/88 bp (1.14%). An alternative analysis of the k-mer frequencies using GenomeScope [26] produced a haploid genome size estimate of 503,748,072 bp, repetitive content of 36.6% and SNP rate of 1/60 bp (1.65%). Both estimates (Figure 2A) are consistent with the flow cytometry estimates and in line with the expectations regarding the heterozygous content of the *H. impetiginosus* genome, a predominantly outcrossed tree [47]. Sequencing errors caused an extreme peak at k = 1 in the k-mer frequency distribution. Both k-mer histograms display two distinct peaks comprising the largest area of each histogram at depths 27 and 55. The bimodal distributions characterize the expected behavior for k-mer frequencies of a heterozygous diploid genome as seen, for example, in the recently reported Oak genome [48]. In the right homozygous peak (at K=55), k-mers are shared between the two homologous chromosomes. The left or heterozygous peak, with half the k-mer depth of the homozygous peak, contains k-mers that are unique to each haplotype due to heterozygosity. The difference in height between these peaks (heterozygous/homozygous ratio) is a measure of the heterozygosity within the genome, which is 1.65% according to the GenomeScope modeling equation.

[Insert Figure 2 here]

Genome assembly. State-of-the-art haploid genome assembler pipelines from short-reads ALLPATHS-LG [27] and SOAPdenovo2 (SOAPdenovo2, RRID:SCR_014986) [49] were considered for an initial evaluation on the dataset of reads. Two relatively new algorithms specifically developed for de novo assembly of heterozygous genomes, MaSuRCA (MaSuRCA, RRID:SCR_010691) [50] and PLATANUS (PLATANUS, RRID:SCR_015531) [51], were also

attempted as alternatives to the other two assemblers designed for genomes of low heterozygosity. Reads were first preprocessed and error corrected using the algorithms provided by each assembler. PLATANUS was set to run but after 10 weeks it did not produce any result in an Intel(R) Xeon(R) server with 64 X7560 2.27GHz CPUs, 256 GB RAM, except for the k-mer count table on the input trimmed reads. After 9 week-long runtimes in an Intel(R) Xeon(R) server with 64 X7560 2.27GHz CPUs, 512 GB RAM, MaSuRCA successfully completed the generation of the super-reads from the trimmed reads but the process was aborted on the overlap-correction process in the Celera Assembler due to excessive CPU usage. SOAPdenovo2 ran very fast (3 days) but produced an assembly with total scaffold size of 860 Mbp. Analysis with SOAPdenovo2 was run with different k-mer sizes, from 31 to 71, step of 10, but none of them produced a reasonable assembly size in view of the expected size estimated by flow cytometry and the k-mer frequency. ALLPATHS-LG was therefore used to assemble the genome with default options. The short reads from fragmented libraries were error-corrected using default settings (K-mer size of 24, ploidy of 2), fragment-filled and assembled into initial unipaths (k-mer size of 96, ploidy of 2). Jumping reads from the mate-pair libraries were then aligned to the unipaths and all alignments were processed in a seed-extension strategy with junction point recognition within the read aimed to remove invalid and duplicate fragments to perform error correction and initial scaffolding. This initial process produced an assembly graph that was turned into scaffolds by analyzing branch points in the graph topology. This late process converted single-base mismatches into ambiguous base codes at branch. It also flattened some other structural features of the assembly including short indels. The contig assembly comprised 109,064 sequences of length 500 bp or longer with total length of 466,314,780 bp. Genome assembly after scaffolding comprised 57,815 scaffolds of length 1 kbp or longer with total length of 610,091,865 bp and N50 of 57 Kbp. The fraction of bases captured in gaps was 23.9% and the rate of ambiguous bases for all bases captured in the assembly was 0.24%. This assembly was only slightly larger in size (<10%) than the empirically determined genome size using flow cytometry [22].

Alternative scaffold and gap-filling. Although the ALLPATHS-LG performance was good in recovering the expected genome size in the assembled contigs there was a high fraction of the bases captured in gaps in the scaffolds (~ ¼ of the total genome assembly). *De novo* assembly algorithms applied to moderate-to-high levels of heterozygosity cannot match the performance

achieved in assemblies of homozygous genomes, especially at the contig assembly level [52]. We thus used the assembled contigs to perform an alternative scaffolding step with SSPACE (SSPACE, RRID:SCR_005056) [53] using the error-corrected short fragment reads and the jumping reads. In this approach, genome assembly comprised 16,090 scaffolds of length 1 kbp or longer with total length of 577,446,088 bp and N50 of 95 Kbp, respectively. The fraction of bases captured in gaps dropped from 23.9% to 18.9% in contrast to ALLPATHS-LG scaffolding, totaling 109,533,288 bp. The rate of ambiguous bases for all bases captured in the assembly dropped from 0.24% to 0.13%. All preprocessed reads were reused in an attempt to close the intra-scaffold gaps using the GapCloser (GapCloser, RRID:SCR_015026) [54] algorithm. Genome assembly after gap-filling was 586,206,884 bp in 15,671 scaffolds of length 1 kbp or longer and only 20,583,469 bp (3.51% of the genome assembly) remained in 24,907 gaps. N50 of scaffolds of length 1 kbp or longer, with gaps, was 97,344 Kb (L50 = 1,792). Sequences longer than 20 kb were assembled in only 6,791 scaffolds totaling 538,102,146 bp, ~97% of the genome size estimated from flow cytometry (557 Mb).

Evaluation of accuracy of the genome assembly. A subset of fragments and jumping read pairs (~15x sequencing coverage each) were used to uncover inaccuracies in the genome assembly. Scaffolds with identified errors were broken or flagged for inspection. REAPR [55] was used to test each base of the genome assembly looking for small local errors (such as a single base substitutions, and short insertions or deletions) and structural errors (such as scaffolding errors) located by means of changes to the expected distribution of inferred sequencing fragments from the mapped reads using SMALT v0.7.6 [56]. REAPR reported that only 343,588,027 (~60%) bases in the assembly should be free of errors, with 5,476 reported (1,658 within contigs, 3,818 over gaps) in the remaining 242,618,857 bp. The most frequent (~92%) type of inaccuracy reported was *Perfect_cov* and *Link*. *Perfect_cov* means low coverage of perfect uniquely mapping reads while *Link* describes situations in which reads map elsewhere in the assembly. The recognition of this inaccuracy at the base pair level should thus reflect the repetitive nature of the genome as inferred from the k-mer frequency spectra analysis (~36-38% of repeats). Besides the base pair inaccurate calls due to repeats, other structural problems in the assembly were identified based on sequence-coverage differences from the expected fragment size distribution and the program used this information to break these. Given the high heterozygosity and divergence between haplotypes on this diploid genome sequence,

homologous sequences can assemble separately or merge. Moreover, unresolved repeat structures in the assembly might also contribute heavily to this issue. Structural errors in REAPR were likely called at the boundaries of these regions. The final genome assembly after REAPR breaks had 19,319 sequences of length 1 kbp or longer, with 576,829,188 bp. N50 size of scaffolds dropped from 97,344 Kb (L50 = 1,792) to 71,491 bp (L50 = 2,379). The number of remaining gaps in the assembly was 21,417 totaling 30,066,113 bp (5.05%).

Paired-end reads from the short fragment libraries were aligned back independently to this genome assembly using SMALT (map -r 0 -x -y 0.5; default alignment penalty scores). Per-scaffold depth of coverage was computed, regardless of mapping quality, using GATK DepthofCoverage. The mean read depth across the scaffolds resulted in 66.45x. The mean read length of the mapped reads was 139.8 bp and the corresponding k-mer coverage for size of 25 was 55.04x which matches with the homozygous peak computed from the k-mer frequency distribution from the unassembled reads. The read depth frequencies are shown in Figure 2B. The heterozygous/homozygous peak height (> 1) in the distribution suggests that the assembly contains redundant copies of unmerged haplotypes due to the structural heterozygosity of the diploid genome of the species. To specifically deal with the heterozygosity we introduced a step to, leniently, recognize and remove alternative heterozygous sequences. Sequences of scaffolds were aligned one versus all using BLAT (BLAT, RRID:SCR_011919) [57] and results were concatenated in a single file of alignments and sorted. Similar sequences were identified on the base of pairwise similarity using filterPSL utility from AUGUSTUS [58] with default parameters, and retaining all best matches to each single sequence queried against all others that satisfy minimal percentage of identity (minId=92%) and minimal percentage of coverage of the query read (minCover=80%). We considered as heterozygous redundant those scaffolds that showed pairwise similarity to exactly another sequence and their depth of coverage fell in a Poisson distribution with parameters given by the heterozygous peak of the read depth distribution over all scaffolds (lambda = 34; Figure 2B). The final step was to keep only one copy – the largest one – of the heterozygous scaffolds among pairs with high similarity.

A preliminary assembly of the *H. impetiginosus* genome. At the end of the accuracy evaluation processes, the genome assembly had a total size of 503,308,897 bp, with gaps, in 13,206 scaffolds. The N50 of scaffolds of 1 kbp or longer was 80,946 bp (L50 = 1,906), the average size

of the sequences was 38,118 bp. Using 20 kbp as an approximate value of longest plant gene length [59, 60], the percentage of scaffolds that equaled or surpassed this value in relation to the empirically determined genome size is 83%, which corresponds to over 92% of the assembly total size. Contigs generated by cutting scaffolds at each gap (of at least 25 base pair, i.e. 25 or more Ns) produced N50 of 40,064 bp (L50 = 3,551) with average sequence size of 19,765 bp. The remaining gaps comprised 26,447,057 bp (5.25% of the genome assembly) in 11,094 segments, with size of 2,384 ± 3,167 bp. The total assembly size represents over 90% of the flow cytometry genome estimate (557 Mb) and should provide a good start to build a further improved reference genome assembly of the species using long-range scaffolding techniques such as whole genome maps using either imaging methods [61] or contact maps of chromosomes based on chromatin interactions [62]. Table 1 summarizes the main statistics of the *Handroanthus impetiginosus* genome assembly with respect to the decisions made in the assembly process.

A reassessment of the assembly accuracy was carried out using REAPR on the final genome assembly. A total of 121 errors within a contig were still recognized, a much smaller number than previously annotated (1,658 errors). Figure 3A shows the frequency distribution for the read depth computed from the paired-end read alignment to the scaffolds sequences. It indicates the expected effect on the distribution in comparison to the previous more redundant assembly. The height of the heterozygous peak was successfully lowered by removing unmerged copies of the same heterozygous loci. Figure 3B shows the relation between the observed number of scaffolds in the final assembly and their read coverage in comparison to a Poisson approximation with lambda = 63 which was the observed average sequencing coverage for reads set from short fragment libraries. Loss of information due to repeat sequences is clearly a limitation of this *H. impetiginosus* assembly. Given the high rate of non-classified consensus sequences we can infer that most families/subfamilies of repeats might be underrepresented.

[Insert Figure 3 here]

To complement the depth of read coverage analyses, we performed additional analyses to identify the most probable causes of breaks in the assembly. We inspected contig termini defining the positions of the terminal nucleotides of each contig from the genome assembly created by cutting at each gap (of at least one base pair, i.e. one or more Ns). This analysis was

developed using a protocol described elsewhere [63] and results are summarized in Figure 4. Contig termini overlap most prominently (~50%) with regions that do not encompass any annotated feature or regions that have no depth of coverage (~15%) based on mapped reads to the assembly. It suggests that contigs end in large repeats not yet resolved given the inherent limitations of short-read sequence data. Another possibility is that these regions can contain low-copy young euchromatic segmental duplication with higher sequence similarity to the consensus sequence. Annotated interspersed repeats (~18%) and short tandem repeats (~9%) were the most prominently annotated features with overlap to contigs ends. Less than 8% (2,473 of 31,668) of annotated gene models were found to overlap contigs ends, indicating that very few are likely to be interrupted in this unfinished assembly. It is a trend that was confirmed using BUSCO analysis which reported only 3% of fragmented genes. Based on variant identification analysis with FreeBayes (FreeBayes, RRID:SCR_010761) using read data mapped to the genome assembly, we found virtually no allelic variants located at contigs end, suggesting that interruption of continuity and contiguity in the assembly is not related to differences between haplotypes.

[Insert Figure 4 here]

Repetitive DNA. A total of 1,608 consensus sequences (average length = 773 bp and totaling 1,281,536 bp) representing interspersed repeats in the genome assembly were found. Search for domains in these sequences with similarity to known large families of genes that could confound the identification of true repeats indicated 85 false positives in the consensus library of repeats. Further 50 sequences were annotated with predicted protein domains frequently associated with protein coding genes. These 135 sequences were wiped out from the consensus library. Most of the remaining 1,473 sequences (71.1%) could not find classification in the hierarchical well-known classes of Transposable Elements [64] but 16.6% could be classified as Class I (retrotransposons) including three orders: LTR (12.8%), LINE (1.6%) and SINE (2.2%); 8.4% are Class II (DNA transposons). Other categories comprised non-autonomous TEs: TRIM (0.4%) and MITE (3.5%). Unknown non-classified sequences in the consensus library cover a wide range of sequence sizes from 42 bp up to 5,987 bp (average = 345 bp, median = 503 bp). The 1,473 sequences representing interspersed repeats in the consensus repeat library were used to mask the genome with RepeatMasker. The masked fraction of the genome assembly comprised

155,348,349 bp, i.e. 30.9% of the total assembled genome of 503 Mbp. Remarkably, if we add to these ~155 Mbp the 54 Mbp of non-captured base pairs in the assembly when considering the empirically determined genome size (= 557–503), the repetitive fraction of the genome approximates 37.5% (209 Mbp out 557 Mbp). This is within the expected range (36.6% - 38.0%) for the repetitive fraction of the genome estimated from the reads set using k-mer profiling approaches.

More than 50% of the masked bases in the assembly, or 80 Mbp, came from non-classified sequences in the consensus library. In the well-known repeats, retrotransposons are the most abundant class in the assembly comprising 50 Mbp (~1/3 of the masked bases) with prominence of LTR/Gypsy (~23 Mb) and LTR/Copy (18 Mb) families of repeats. DNA transposons and non-autonomous orders of transposons masked 12 Mbp and 11 Mbp (~1/6 of the masked bases), respectively, highlighting the prominence of DNA/hAT families of class II and MITE (Figure 5). Simple sequence repeats (SSRs) detection using RepeatMasker identified a total of 182,115 microsatellites with a density of 2.76 kb per SSR in the genome assembly. This density corroborates the general finding that the overall frequency of microsatellites is inversely related to genome size in plant genomes [65]. This SSRs density in *H. impetiginosus* (genome size of 557 Mbp/SSR density of 362 per Mbp) is higher than in larger plant genomes such as those of maize (1,115 Mbp/163 SSRs per Mbp), *S. bicolor* (738 Mbp/175 per Mbp), *G. raimondii* (761 Mbp/74.8 per Mbp) [66] but lower than densities in smaller genomes such as those of *A. thaliana* (120 Mbp/ 418 per Mbp), *Medicago truncatula* (307 Mbp/ 495 per Mbp) and *C. sativus* (367 Mbp/ 552 per Mbp) [67]. Different SSR motifs ranging from 1 to 6 bp showed that the di-nucleotide repeats were the most abundant repeats followed by the mono- (Figure S3A). The frequency of SSR decreased with increase in motif length (Supplementary Figure S3B), which is a trend usually observed both in monocots and dicots [67].

[Insert Figure 5 here]

Transcriptome assembly and gene content annotation and analysis. A single run of Illumina HiSeq 2500 sequencing, from a pool of RNA samples, generated nearly 148 million of paired end reads. After adapter removal, trimming and coverage normalization, 55.2 million high-quality reads (38%) were used to assemble the transcriptome using *de novo* (Trinity and SOAP-Trans-

denovo transcripts combined with the EvidentialGene pipeline) and genome guided methods (PERTRAN). The PASA pipeline was used to integrate transcripts alignments to the genome assembly from these set of sequences, generating 54,320 EST assemblies representing putative protein-coding loci in the genome assembly. Loci were identified by the assembled transcript alignments using BLASTX [36] and EXONERATE [37] alignments of plant peptides to the repeat-soft-masked genome using RepeatMasker. After gene model prediction and refinements, a total of 36,262 gene models were found in the genome assembly and 31,668 of them were retained after quality assessment based on Cscore, protein coverage, and overlap to repeats as described in Methods. The number of predicted mRNA transcripts was 35,479.

Structural features of the gene content are shown in Table 2 and Table 3. The average number of exons per gene was ~5 and its average length was 285 bp. The average number of introns per gene was ~4 and its average length was 445 bp. The GC content is significantly different between exons and introns (t-test p-value < 0.0001). Coding sequences have ~43% of GC, while introns have less with ~33% (Table 2). GC content tends to be higher in coding (exonic) than in non-coding regions [68], which may be related to gene architecture and alternative splicing [69-71]. A comparison of the gene features parameters, such as number and length (Figure S4A), was carried out between *H. impetiginosus* and *Erythranthe guttata*, another plant in the order Lamiales (Asterids), the model plant *A. thaliana* and the model tree *P. trichocarpa* (Rosids). As depicted in the frequency histograms, the exons parameters are stable among these species (Figure S4B). For the introns (Figure S4C), frequency histograms have a sharp peak around 90 bp and a larger peak that is much lower in density. There is a small intron-size variability from species to species in the distributions, especially for larger introns, which rarely go beyond than 10,000 bp. The intron length distributions in these four species is similar to those observed in lineages that are late in the evolutionary time scale, such as plants and vertebrates [72]. The sharp peak in the distributions at their "minimal intron" size is supposed to affect function by enhancing the rate at which mRNA is exported from the cell nucleus [73, 74]. In the model plant *A. thaliana,* a minimal intron group was previously defined [73] as anything that lies within three standard deviations of the optimum peak at 89±12 bp (53 bp – 125 bp). According to this definition, Table 3 summarizes the distribution of the minimal intron among genes of *H. impetiginosus* and other selected plant species in the Asterids and Rosids lineages. We have calculated the percentages of minimal introns out of the total introns and the fraction of

minimal-intron-containing genes with at least one minimal intron. Computed values were similar between *H. impetiginosus* and those of selected species with higher number of large introns (smaller minimal intron peak) but were more distinctive with those species such as *A. thaliana* and *E. guttata* in which the number of large introns was lower (larger minimal intron peak). This is thought as a general trend and was also observed in previous work [73]. These comparative analyses about the structural properties of the predicted genes indicate that the genome assembly of *H. impetiginosus* contains highly accurate gene structures.

[Insert Figure 6 here]

To further validate the gene content annotation, we used the transcript assemblies and selected plant proteomes to inspect if these sequences could align in its entirety to the genomic sequence. Out of the 31,668 primary mRNA transcripts (considering only the longest one when isoforms were predicted) in the genome, 11,488 have 100% of their CDS covered by EST assemblies. The remaining 20,054 transcripts have either a minimum of 80% of their CDS covered by EST assemblies or a cscore ≥ 0.5. From these latter, the encoded putative peptides have excellent sequence similarity support from BLASTP comparisons with dicot species *Erythranthe guttata* (5,224 genes), *Sesamum indicum* (4,625 genes), potato or tomato (2,777 genes), soybean (1,484 genes) and the poplar tree (1,424 genes) reflecting the taxonomic relationship between *H. impetiginosus* and these other related dicots. Gene models support was also found from more distantly related dicots (1,826 genes) and monocots (1,042 genes). Altogether, 31,048 gene models (98%) show well-supported similarity hits to other known plant protein sequences. Additional 517 predicted protein sequences did not produce hits and 103 sequences produced ambiguous hits from non-target species or represent possible contaminants in the assembly such as endophytic fungi (ascomycetes, 42 sequences; basidiomycetes, 17 sequences). Figure 6A summarizes the main finding regarding the similarity analyses with known proteins.

BUSCO (BUSCO, RRID:SCR_015008) [75] single-copy genes plant profiles were used to estimate completeness of the expected gene space as well as the duplicate fraction of the genome assembly. Out of the 956 profiles searched on the assembly, 59 (6.1%) were reported missing and 30 (3.1%) returned fragmented. From the profiles with complete match to the assembly,

867 (90.7%) were reported as single-copy and 247 (25.8%) were found completely duplicated. We benchmarked our results by searching the BUSCO profiles on the genomes of other lamids, *Erythranthe guttata* and *Olea europaea.* In *E. guttata* the analysis reported completeness level of 88% (848 single-copy profiles with complete match) while fragmented genes were 52 (5.4%). In *O. europeae, the* completeness level was 94% (905 complete single-copy profiles) and fragmented genes were only 14 (1.4%). Summary of BUSCO analysis is presented in Figure 6B.

Databases for gene ontology (GO) annotation are rich resources to describe functional properties of experimentally derived gene sets. To explore relationships between the GO terms in the *H. impetiginosus* and related, well-curated, genomes we used WEGO [76] to perform a genome-wide comparative analyses among broad functional GO terms with other lamids. The P-value of Pearson Chi-Square test was considered to indicate significant relationships between the proportions of genes of each GO term in these two datasets and to suggest patterns of enrichment (Figures S5 and S6). These analyses revealed several GO terms in which the proportion of genes in the two compared species were related. For the terms in which the comparison did not indicate a significant relationship of gene proportions between the two datasets, the compared GO terms suggested enrichments in *H. impetiginosus* for GO terms involved in metabolic processes and catalytic activity in comparison to *E. guttata* and *O. europaea*.

[Insert Figure 7 here]

The central role of enzymes as biological catalysts is a well-studied issue related to the chemistry of cells [77]. An important feature of most enzymes is that their activities can be regulated to function properly to comply with physiological needs of the organism. We observed that GO term for enzyme regulatory activity encompass a higher proportion of genes in *H. impetiginosus* than in the two other lamids, albeit the difference did not reach significance in *E. guttata*. Research in *Arabidopsis*, an herbaceous plant, has found little connectivity between metabolites and enzyme activity [78]. In comparison to Arabidopsis broader GO terms, *H. impetiginosus* showed, as discussed above, enrichment for the proportion genes assigned to metabolic process (49.1% > 47.4%; p-value 0.002) and catalytic activity (46.2% > 42.9%; p-value = 0). The proportion of genes for enzyme regulatory activity was also higher in *H. impetiginosus* than *A.*

thaliana, though not statistically significant (p-value = 0.083). Investigations into whether and how metabolic process and enzyme activities relate and how it could influence the known richness of metabolites for forest trees of the mega diverse tropical biomes, particularly in the genus *Tabebuia* and *Handroanthus*, shall be an interesting issue for future molecular and chemistry studies.

Benchmarking the genome assembly of *H. impetiginosus*. Based on current standards for plant genome sequence assembly [60, 79, 80] we have provided a quality assembly of high future utility. To support functional analyses we classified the gene models into high-confidence and low-confidence groups. Out of the 31,688 protein-coding loci annotated in the genome assembly, 28,603 (90%) produced high-confidence gene models (Supplementary File S1). This subset contains approximately the same number of genes reported in less fragmented genome assemblies for other lamids. *E. guttata* (2n=28) reports 28,140 protein-coding genes [81]; *O. europeae* (2n = 46) has 56,349 protein-coding genes [82] but its genome has likely undergone a whole genome duplication event. Most of *Tabebuia* and *Handroanthus* species studied so far have 2n = 40 [22]. The fraction of gene duplicates in the BUSCO analysis (see Figure 5B) was intended to estimate the level of redundancy in the genome assembly. We benchmarked our results by searching the completed duplicated BUSCO profiles in the genomes of *E. guttata* and *O. europaea*. In the first, we found them to be 15% (150 out 956), while in the latter the duplicated profiles were 38% (364 out of 956). In these three lamids, it appears that the frequency of small- and large-scale duplications, such as (paleo)polyploidy, can explain the differences in the number of annotated genes and levels of gene duplication (*E. guttata* <= *H. impetiginosus* << *O. europaea*). It suggests that the *H. impetiginosus* genome has not undergone a recent whole-genome duplication event, although a deeper analysis of this question, beyond the scope of this study, remains open.

Our genome assembly metrics were benchmarked against comparable genome assemblies of other highly heterozygous forest tree genomes (File S2 and Figure S7). The *H. impetiginosus* assembly has 503 Mbp in 13,206 scaffolds ≥2 kbp, representing over 90% of the flow cytometry estimated size (557 Mb). For *Quercus robur*, the assembly had 17,910 scaffolds ≥2 kbp with scaffolds N50 of 260 kbp, but corresponding to 1.34 Gbp, i.e. 81% larger than the expected 740 Mbp genome, which is clearly an undesirable result [83]. For *Quercus lobata* with a genome size

of 730 Mbp two assemblies were provided: a haplotype-reduced assembly, with 40,158 contigs totaling 760 Mb, N50 of 95 kbp and a more complete version for gene models, containing 94,394 scaffolds ≥2 kbp, totaling 1.15 Gbp, with an N50 of 278 kbp [48]. Despite our lower NG50/N50 scaffold length <100 kbp, the *H. impetiginosus* assembly has a large (60%) percentage of scaffolds ≥20 kbp. This value is higher than the reported values for *Quercus lobata* v0.5 (53%), *Quercus lobata v1.0* (51%) and *Quercus rubra* (48%), even if those assemblies had higher NG50/N50 scaffold lengths. Finally, contigs termini analysis has found virtually no allelic variants located at contigs ends, suggesting that interruption of continuity and contiguity in the assembly is not related to differences between haplotypes. This genome assembly for *Handroanthus impetiginosus* will thus be useful for variant calling, one of the main future objectives for generating this resource.

Genome-guided exploration of specialized metabolism genes of quinoid systems. Aside from its high valued wood, *H. impetiginosus* and other Ipê species are also known for their medicinal effects. Extracts from its bark and wood have many ethnobotanical uses: against cancer, malaria, fevers, trypanosomiasis, fungal and bacterial infections and stomach disorders [84, 85]. The wood extracts have also been demonstrated to have anti-inflammatory effects [86] [87]. The main bioactive components isolated from the Pink Ipê are Lapachol and its products [88], which are naphthoquinones derived from the o-succinylbenzoate (OSB) pathway [89]. Lapachol is also responsible for the well-known high resistance of the Ipê wood against rotting fungi and insects [90]. In addition, naphthoquinones are aromatic substances with ecological importance for the interaction of plants with other plants, insects and microbes [89]. Given their medicinal and biological relevance, we have searched the *H. impetiginosus* annotated genes for the enzymes involved in the biosynthesis of naphthoquinones. By searching for the KEGG identifiers of these enzymes (e.g. K01851) in the InterPro annotation results, we found all the important known enzymes that lead to the biosynthesis of lapachol (Figure 7). Unfortunately, however, the last two steps of the lapachol biosynthesis pathway still constitute unidentified enzymes [89]. For comparative purposes, we downloaded the annotation file of five other species from the Phytozome database. The number of *H. impetiginosus* genes encoding for the enzymes of each step in the pathway is comparable to the numbers found in other species. However, three exceptions were found. *H. impetiginosus* has five genes encoding the enzyme that converts chorismate to isochorismate, the first step in the o-succinylbenzoate (OSB) pathway. Two other

steps found to have relatively more genes in *H. impetiginosus* are the ones that lead to the synthesis of 1,4-Dihydroxy-2-naphtoyl-CoA and of 2-Phytyl-1,4-naphthoquinone. The availability of sequences for these genes may open new avenues for biotechnological products and for a better understanding of their ecological roles.

RE-USE POTENTIAL

We have reported a well-curated but still unfinished genome assembly for *Handroanthus impetiginosus*, a highly valued, ecologically keystone tropical timber and a species rich in natural products. The fragmentation of this preliminary assembly might be still be limiting for deeper insights of whole-genome comparative analyses or studies of genome evolution [91], although we think that such studies may be carried out using this assembly at least at the gene-level or gene-family level. Nevertheless, the broad validation performed provides a useful genomic resource for genetic and functional analysis including, but not limited to, downstream applications such as variant calling, molecular markers development and functional studies. Extensive documentation of quality throughout the assembly process was provided showing that acceptable continuity was reached and that the fragmentation of the final sequence mostly derives from loss of information on high-copy families of long interspersed repeats or the presence of low-copy segmental duplications likely recently evolved with higher sequence similarity to the consensus sequence. Certainly, there are still inaccuracies at the base and assembly level but all efforts were made to deliver results to end user with the appropriate documentation, making this initial read set, sequence and annotations as a primary and reliable starting grounds for further improvement.

We have documented in detail the main features of the reported assembly. The total assembly size of scaffolds with ≥2 kbp in length is 90% of the flow cytometry determined genome size, we believe a remarkable accomplishment given the anticipated difficulties in assembling such a repetitive and highly heterozygous diploid genome based exclusively on short-read sequencing. The percentage of base pairs in scaffolds with ≥20 kbp is 83% (461 Mbp of 557 Mbp) of the empirically determined genome size, which corresponds to 92% of the assembled total size (461 Mbp of 503 Mbp). Using 20 kbp as an approximate value of the longest plant gene length, this result shows that 60% of the assembly is accessible for reliable gene annotation. Furthermore

the N50/NG50 (41 kbp/34 kbp) contig length is longer than 30 kbp, which has been suggested to be an adequate minimum threshold for high utility of a genome assembly [79]. The percentage of documented gaps in scaffolds is only 5.3% and the few misassembled signatures present in the assembly were fully documented based on acceptable metrics such as fragment coverage distribution error (FCD error). Less than 8% (2,473 of 31,668) of annotated gene models were found to overlap contigs ends, indicating that very few are likely to be interrupted in this unfinished assembly. No allelic variants were found at contigs ends, suggesting that interruption of continuity and contiguity in the assembly is not related to differences between haplotypes, therefore providing a valuable resource for variant calling and functional analysis. Over 86% (27,380 of 31,668) of the gene models represented in the assembly have external evidential support measured by Pasa-validated EST alignments from RNA-Seq or high-coverage alignments with known plant proteins (>90% coverage). Furthermore, 80% (25,369 of 31,668) of transcripts have conceptual translation that contain protein domain annotation, excluded those associated to TEs. Finally, a summary of BUSCO analysis indicates that the detected number of plant single copy orthologs represents 90% of the searched profiles (867 of 956) while only 6% is missing and 3% is fragmented.

This is the first well-curated genome for a Neotropical forest tree and the first one reported for a member of the Bignoniaceae family. Besides expanding the opportunities for comparative genomic studies by including an overlooked taxonomic family, the availability of this genome assembly will foster functional studies with new targets and allow the development and application of robust sets of genome-wide SNP genotyping tools to support multiple population genomics analyses in *H. impetiginosus* and related species of the Tabebuia Alliance. This group includes several of the most ecologically and economically important timber species of the American tropics. Going beyond the species-specific significance of these results, this study paves the way for developing similar genomic resources for other Neotropical forest trees of equivalent relevance. This in turn will open exceptional prospects to empower a higher-level understanding of the evolutionary history, species distribution and population demography of the still largely neglected forest trees of the mega diverse tropical biomes. Furthermore, this genome assembly provides a new resource for advances in the current integration between genomics, transcriptomics and metabolomics approaches for exploration of the enormous structural diversity and biological activities of plant-derived compounds.

AVAILABILITY OF SUPPORTING DATA

Sequences for the genome and assembly along with gene content annotation as well as the raw sequencing reads have been deposited into GenBank, BioProject PRJNA324125. This Whole Genome Shotgun (WGS) project has been deposited at DDBJ/ENA/GenBank under the accession NKXS00000000. The version described in this paper is version NKXS01000000. BioSample for WGS is SAMN05195323 and corresponding SRA run accessions are SRR3624821 - SRR3624825. BioSample for RNA-Seq is SAMN07346903 with SRA run accession SRR5820886. Supporting data and summary outputs for main analyses in this Data Note are available via the *GigaScience* repository GigaDB [92]. The Perl script that automated the read set from mate-pair sequencing preprocessing (TrimAdaptor.pl) was uploaded to GigaDB under permission of the original authors at the High-Throughput Sequencing and Genotyping Center Unit of the University of Illinois Urbana-Champaign.

List of abbreviations

BLASTP, Basic Local Alignment Search Tool for Proteins; BLAT, BLAST-like alignment tool; CDS, coding DNA sequence; EC, Enzyme Comissioned Number; EST, Expressed Sequence Tag; GATK, Genome Analysis Toolkit; GO, Gene Ontology; LINE, Long Interspersed Nuclear Elements; LTR, Long Terminal Repeats; MBH, Mutual Best Hit; MITE, Miniature Inverted–Repeat Transposable Elements; mRNA, messenger RNA; PASA, Program to Assemble Spliced Alignment; REAPR, Recognition of Errors in Assemblies using Paired Reads; SINE, Short Interspersed Nuclear Elements; SNP, Single Nucleotide Polymorphism; SSPACE, SSAKE-based Scaffolding of Pre-Assembled Contigs after Extension; TE, transposable element.

Ethics approval

Not applicable

Consent for publication

Not applicable

Competing interests

The authors declare that they have no competing interests.

Funding and acknowledgements

This work was supported by competitive grants from CNPq to RGC (project no. 471366/2007-2 and Rede Cerrado CNPq/PPBio project no. 457406/2012-7), to EN (CNPq Proc. 476709/2012-1) and to DG (PRONEX FAP-DF Project Grant "NEXTREE" 193.000.570/2009). RGC and DG have been supported by productivity grants from CNPq, which we gratefully acknowledge. OBSJr has been supported by an EMBRAPA doctoral fellowship and was an Affiliate Researcher at Lawrence Berkeley National Laboratory (LBNL), Berkeley CA, at the time of this research. OBSJr thanks to DM Goodstein and the members of the Phytozome team at the LBNL/Joint Genome Institute (JGI) for their valuable help and support in working with the JGI pipelines for genomic research. WE also thank Dr. Gabriela Ferreira Nogueira and André Luis X. de Souza for their help with flow cytometry analysis.

Authors' contributions

OBSJr performed sequence data analysis and genome assembly and together with EN carried out transcriptome and protein-coding gene annotation. RC and DG conceived the project, collected samples, extracted genomic DNA and RNA, carried out flow cytometry analysis and supervised the project. All authors were involved in discussions, writing and editing. All authors read and approved the final manuscript.

REFERENCES

1. Goodstein DM, Shu SQ, Howson R, Neupane R, Hayes RD, Fazo J, Mitros T, Dirks W, Hellsten U, Putnam N *et al*: **Phytozome: a comparative platform for green plant genomics**. *Nucleic Acids Research* 2012, **40**(D1):D1178-D1186.
2. Kang YJ, Lee T, Lee J, Shim S, Jeong H, Satyawan D, Kim MY, Lee SH: **Translational genomics for plant breeding with the genome sequence explosion**. *Plant Biotechnology Journal* 2016, **14**(4):1057-1069.
3. Bevan M, Walsh S: **The Arabidopsis genome: A foundation for plant research**. *Genome Research* 2005, **15**(12):1632-1642.
4. Morrell PL, Buckler ES, Ross-Ibarra J: **Crop genomics: advances and applications**. *Nature Reviews Genetics* 2012, **13**(2):85-96.
5. Varshney RK, Glaszmann JC, Leung H, Ribaut JM: **More genomic resources for less-studied crops**. *Trends in Biotechnology* 2010, **28**(9):452-460.

6. Myburg AA, Grattapaglia D, Tuskan GA, Hellsten U, Hayes RD, Grimwood J, Jenkins J, Lindquist E, Tice H, Bauer D *et al*: **The genome of *Eucalyptus grandis***. *Nature* 2014, **510**(7505):356-362.

7. Tuskan GA, DiFazio S, Jansson S, Bohlmann J, Grigoriev I, Hellsten U, Putnam N, Ralph S, Rombauts S, Salamov A *et al*: **The genome of black cottonwood, *Populus trichocarpa* (Torr. & Gray)**. *Science* 2006, **313**(5793):1596-1604.

8. Neale DB, Wegrzyn JL, Stevens KA, Zimin AV, Puiu D, Crepeau MW, Cardeno C, Koriabine M, Holtz-Morris AE, Liechty JD *et al*: **Decoding the massive genome of loblolly pine using haploid DNA and novel assembly strategies**. *Genome Biology* 2014, **15**(3).

9. Nystedt B, Street NR, Wetterbom A, Zuccolo A, Lin YC, Scofield DG, Vezzi F, Delhomme N, Giacomello S, Alexeyenko A *et al*: **The Norway spruce genome sequence and conifer genome evolution**. *Nature* 2013, **497**(7451):579-584.

10. Moghe G, Last R: **Something old, something new: Conserved enzymes and the evolution of novelty in plant specialized metabolism**. *Plant Physiology* 2015:pp.00994.02015.

11. Stone R: **Lifting the Veil on Traditional Chinese Medicine**. *Science* 2008, **319**(5864):709-710.

12. Chappell J, DellaPenna D, O'Connor S: **Specific Aims for Medicinal Plant Genomics Resource**. *Medicinal Plants Genomics Resource* 2017.

13. Brousseau L, Tinaut A, Duret C, Lang T, Garnier-Gere P, Scotti I: **High-throughput transcriptome sequencing and preliminary functional analysis in four Neotropical tree species**. *BMC Genomics* 2014, **15**(1):238.

14. Olsson S, Seoane-Zonjic P, Bautista Ro, Claros G, González-Martínez S, Scotti I, Scotti-Saintagne C, Hardy O, Heuertz M: **Development of genomic tools in a widespread tropical tree, Symphonia globulifera L.f.: a new low-coverage draft genome, SNP and SSR markers**. *Molecular Ecology Resources* 2017, **17**(4):614-630.

15. Cadena-González A, Sorensen M, Theilade I: **Use and valuation of native and introduced medicinal plant species in Campo Hermoso and Zetaquira, Boyacá, Colombia**. *Journal of Ethnobiology and Ethnomedicine* 2013, **9**(1):23.

16. Bodker G, Bhat KKS, Burley J, Vantomme P: **Medicinal plants for forest conservation and health care**. *Food and Agriculture Organization of the United Nations* 1997.

17. Braga AC, Reis AMM, Leoi LT, Pereira RW, Collevatti RG: **Development and characterization of microsatellite markers for the tropical tree species Tabebuia aurea (Bignoniaceae)**. *Molecular Ecology Notes* 2007, **7**(1):53-56.

18. Liu B, Shi Y, Yuan J, Hu X, Zhang H, Li N, Li Z, Chen Y, Mu D, Fan W: **Estimation of genomic characteristics by analyzing k-mer frequency in de novo genome projects**. *arXiv:13082012* 2013.

19. Schulze M, Grogan J, Uhl C, Lentini M, Vidal E: **Evaluating ipe (Tabebuia, Bignoniaceae) logging in Amazonia: Sustainable management or catalyst for forest degradation?** *Biological Conservation* 2008, **141**(8):2071-2085.

20. Inagaki R, Ninomiya M, Tanaka K, Watanabe K, Koketsu M: **Synthesis and Cytotoxicity on Human Leukemia Cells of Furonaphthoquinones Isolated from Tabebuia Plants**. *Chemical & Pharmaceutical Bulletin* 2013, **61**(6):670-673.

21. Park BS, Kim JR, Lee SE, Kim KS, Takeoka GR, Ahn YJ, Kim JH: **Selective growth-inhibiting effects of compounds identified in Tabebuia impetiginosa inner bark on human intestinal bacteria**. *Journal of Agricultural and Food Chemistry* 2005, **53**(4):1152-1157.

22. Collevatti RG, Dornelas MC: **Clues to the evolution of genome size and chromosome number in Tabebuia alliance (Bignoniaceae)**. *Plant Systematics and Evolution* 2016, **302**(5):601-607.
23. Aronesty E: **Comparison of sequencing utility programs**. *The Open Bioinformatics Journal* 2013, **7**:1-8.
24. Langmead B, Trapnell C, Pop M, Salzberg SL: **Ultrafast and memory-efficient alignment of short DNA sequences to the human genome**. *Genome Biology* 2009, **10**(3).
25. Marcais G, Kingsford C: **A fast, lock-free approach for efficient parallel counting of occurrences of k-mers**. *Bioinformatics* 2011, **27**(6):764-770.
26. Vurture GW, Sedlazeck FJ, Nattestad M, Underwood CJ, Fang H, Gurtowski J, Schatz MC: **GenomeScope: fast reference-free genome profiling from short reads**. *Bioinformatics btx153* 2017.
27. Gnerre S, MacCallum I, Przybylski D, Ribeiro F, Burton J, Walker B, Sharpe T, Hall G, Shea T, Sykes S *et al*: **High-quality draft assemblies of mammalian genomes from massively parallel sequence data**. *Proceedings of the National Academy of Sciences* 2011, **108**(4):1513-1518.
28. Flutre T, Duprat E, Feuillet C, Quesneville H: **Considering Transposable Element Diversification in De Novo Annotation Approaches**. *Plos One* 2011, **6**(1).
29. Wicker T, Sabot F, Hua-Van A, Bennetzen JL, Capy P, Chalhoub B, Flavell A, Leroy P, Morgante M, Panaud O *et al*: **A unified classification system for eukaryotic transposable elements**. *Nature Reviews Genetics* 2007, **8**(12):973-982.
30. Hoede C, Arnoux S, Moisset M, Chaumier T, Inizan O, Jamilloux V, Quesneville H: **PASTEC: An Automatic Transposable Element Classification Tool**. *Plos One* 2014, **9**(5).
31. Smit AFA, Hubley R, Green P: **RepeatMasker Open-4.0 (2013-2015)**. 2015.
32. Bolger AM, Lohse M, Usadel B: **Trimmomatic: a flexible trimmer for Illumina sequence data**. *Bioinformatics* 2014, **30**(15):2114-2120.
33. Xie Y, Wu G, Tang J, Luo R, Patterson J, Liu S, Huang W, He G, Gu S, Li S *et al*: **SOAPdenovo-Trans: de novo transcriptome assembly with short RNA-Seq reads**. *Bioinformatics* 2014, **30**(12):1660-1666.
34. Grabherr MG, Haas BJ, Yassour M, Levin JZ, Thompson DA, Amit I, Adiconis X, Fan L, Raychowdhury R, Zeng QD *et al*: **Full-length transcriptome assembly from RNA-Seq data without a reference genome**. *Nature Biotechnology* 2011, **29**(7):644-U130.
35. Gilbert D: **EvidentialGene: mRNA Transcript Assembly Software**. *EvidentialGene : Evidence Directed Gene Construction for Eukaryotes* 2013.
36. Schmutz J, McClean P, Mamidi S, Wu A, Cannon S, Grimwood J, Jenkins J, Shu S, Song Q, Chavarro C *et al*: **A reference genome for common bean and genome-wide analysis of dual domestications**. *Nature Genetics* 2014, **46**(7):707-713.
37. Shu S, Goodstein DM, Hayes D, Mitros T, Rokhsar D: **JGI Plant Genomics Gene Annotation Pipeline**. *SciTech Connect* 2017.
38. Gish W, States D: **Identification of protein coding regions by database similarity search**. *Nature Genetics* 1993, **3**(3):266-272.
39. Slater GS, Birney E: **Automated generation of heuristics for biological sequence comparison**. *Bmc Bioinformatics* 2005, **6**.
40. **RepeatMasker Open-4.0. http://www.repeatmasker.org** [http://www.repeatmasker.org]
41. UniProt Consortium: **UniProt: a hub for protein information**. *Nucleic Acids Research* 2014, **43**(D1):D204-D212.

42. Salamov AA, Solovyev VV: **Ab initio gene finding in Drosophila genomic DNA**. *Genome Research* 2000, **10**(4):516-522.
43. Solovyev V, Kosarev P, Seledsov I, Vorobyev D: **Automatic annotation of eukaryotic genes, pseudogenes and promoters**. *Genome Biology* 2006, **7**.
44. Yeh RF, Lim LP, Burge CB: **Computational inference of homologous gene structures in the human genome**. *Genome Research* 2001, **11**(5):803-816.
45. Haas BJ, Delcher AL, Mount SM, Wortman JR, Smith RK, Hannick LI, Maiti R, Ronning CM, Rusch DB, Town CD *et al*: **Improving the Arabidopsis genome annotation using maximal transcript alignment assemblies**. *Nucleic Acids Research* 2003, **31**(19):5654-5666.
46. Jones P, Binns D, Chang HY, Fraser M, Li W, McAnulla C, McWilliam H, Maslen J, Mitchell A, Nuka G *et al*: **InterProScan 5: genome-scale protein function classification**. *Bioinformatics* 2014, **30**(9):1236-1240.
47. Braga AC, Collevatti RG: **Temporal variation in pollen dispersal and breeding structure in a bee-pollinated Neotropical tree**. *Heredity* 2011, **106**(6):911-919.
48. Sork VL, Fitz-Gibbon ST, Puiu D, Crepeau M, Gugger PF, Sherman R, Stevens K, Langley CH, Pellegrini M, Salzberg SL: **First Draft Assembly and Annotation of the Genome of a California Endemic Oak Quercus lobata Nee (Fagaceae)**. *G3-Genes Genomes Genetics* 2016, **6**(11):3485-3495.
49. Luo RB, Liu BH, Xie YL, Li ZY, Huang WH, Yuan JY, He GZ, Chen YX, Pan Q, Liu YJ *et al*: **SOAPdenovo2: an empirically improved memory-efficient short-read de novo assembler**. *Gigascience* 2012, **1**.
50. Zimin AV, Marcais G, Puiu D, Roberts M, Salzberg SL, Yorke JA: **The MaSuRCA genome assembler**. *Bioinformatics* 2013, **29**(21):2669-2677.
51. Kajitani R, Toshimoto K, Noguchi H, Toyoda A, Ogura Y, Okuno M, Yabana M, Harada M, Nagayasu E, Maruyama H *et al*: **Efficient de novo assembly of highly heterozygous genomes from whole-genome shotgun short reads**. *Genome Research* 2014, **24**(8):1384-1395.
52. Malinsky M, Simpson JT, Durbin R: **Trio-sga: facilitating de novo assembly of highly heterozygous genomes with parent-child trios**. *bioRxiv* 2016.
53. Boetzer M, Henkel CV, Jansen HJ, Butler D, Pirovano W: **Scaffolding pre-assembled contigs using SSPACE**. *Bioinformatics* 2011, **27**(4):578-579.
54. Nadalin F, Vezzi F, Policriti A: **GapFiller: a de novo assembly approach to fill the gap within paired reads**. *Bmc Bioinformatics* 2012, **13**.
55. Hunt M, Kikuchi T, Sanders M, Newbold C, Berriman M, Otto TD: **REAPR: a universal tool for genome assembly evaluation**. *Genome Biology* 2013, **14**(5):R47.
56. Ponstingl H, Ning ZM: **SMALT**. *2010 - 2015 Genome Research Ltd* 2016.
57. Kent WJ: **BLAT--the BLAST-like alignment tool**. *Genome research* 2002, **12**(4):656-664.
58. Stanke M, Keller O, Gunduz I, Hayes A, Waack S, Morgenstern B: **AUGUSTUS: ab initio prediction of alternative transcripts**. *Nucleic Acids Research* 2006, **34**:W435-W439.
59. Ramírez-Sánchez O, Pérez-Rodríguez P, Delaye L, Tiessen A: **Plant Proteins Are Smaller Because They Are Encoded by Fewer Exons than Animal Proteins**. *Genomics, Proteomics & Bioinformatics* 2016, **14**(6):357-370.
60. Bradnam KR, Fass JN, Alexandrov A, Baranay P, Bechner M, Birol I, Boisvert S, Chapman JA, Chapuis G, Chikhi R *et al*: **Assemblathon 2: evaluating de novo methods of genome assembly in three vertebrate species**. *GigaScience* 2013, **2**:10-10.

61. Lam ET, Hastie A, Lin C, Ehrlich D, Das SK, Austin MD, Deshpande P, Cao H, Nagarajan N, Xiao M *et al*: **Genome mapping on nanochannel arrays for structural variation analysis and sequence assembly**. *Nature Biotechnology* 2012, **30**(8):771-776.

62. Ay F, Noble WS: **Analysis methods for studying the 3D architecture of the genome**. *Genome Biology* 2015, **16**.

63. Tørresen OK, Star B, Jentoft S, Reinar WB, Grove H, Miller JR, Walenz BP, Knight J, Ekholm JM, Peluso P *et al*: **An improved genome assembly uncovers prolific tandem repeats in Atlantic cod**. *BMC Genomics* 2017, **18**(1):95.

64. Wicker T, Sabot F, Hua-Van A, Bennetzen J, Capy P, Chalhoub B, Flavell A, Leroy P, Morgante M, Panaud O *et al*: **A unified classification system for eukaryotic transposable elements**. *Nature Reviews Genetics* 2007, **8**(12):973-982.

65. Morgante M, Hanafey M, Powell W: **Microsatellites are preferentially associated with nonrepetitive DNA in plant genomes**. *Nature Genetics* 2002, **30**(2):194-200.

66. Wang Q, Fang L, Chen J, Hu Y, Si Z, Wang S, Chang L, Guo W, Zhang T: **Genome-Wide Mining, Characterization, and Development of Microsatellite Markers in Gossypium Species**. *Scientific Reports* 2015, **5**(1).

67. Sonah H, Deshmukh R, Sharma A, Singh V, Gupta D, Gacche R, Rana J, Singh N, Sharma T: **Genome-Wide Distribution and Organization of Microsatellites in Plants: An Insight into Marker Development in Brachypodium**. *PLOS ONE* 2011, **6**(6):e21298.

68. Bernardi G: **Isochores and the evolutionary genomics of vertebrates**. *Gene* 2000, **241**(1):3-17.

69. Mizuno M, Kanehisa M: **Distribution profiles of GC content around the translation initiation site in different species**. *FEBS letters* 1994, **352**(1):7-10.

70. Amit M, Donyo M, Hollander D, Goren A, Kim E, Gelfman S, Lev-Maor G, Burstein D, Schwartz S, Postolsky B *et al*: **Differential GC content between exons and introns establishes distinct strategies of splice-site recognition**. *Cell reports* 2012, **1**(5):543-556.

71. Wendel JF, Greilhuber J, Dolezel J, Leitch IJ: **Plant Genome Diversity Volume 1 - Plant Genomes, their Residents, and their Evolutionary Dynamics**, vol. 1. Wien: Springer-Verlag; 2012.

72. JiaYan W, JingFa X, LingPing W, Jun Z, HongYan Y, ShuangXiu W, Zhang Z, Jun Y: **Systematic analysis of intron size and abundance parameters in diverse lineages**. *Science China Life Sciences* 2013, **56**(10):968-974.

73. Yu J, Yang Z, Kibukawa M, Paddock M, Passey D, Wong G: **Minimal Introns Are Not "Junk"**. *Genome Research* 2002, **12**(8):1185-1189.

74. Zhu J, He F, Wang D, Liu K, Huang D, Xiao J, Wu J, Hu S, Yu J: **A Novel Role for Minimal Introns: Routing mRNAs to the Cytosol**. *PLOS ONE* 2010, **5**(4):e10144.

75. Simao FA, Waterhouse RM, Ioannidis P, Kriventseva EV, Zdobnov EM: **BUSCO: assessing genome assembly and annotation completeness with single-copy orthologs**. *Bioinformatics* 2015, **31**(19):3210-3212.

76. Ye J, Fang L, Zheng H, Zhang Y, Chen J, Zhang Z, Wang J, Li S, Li R, Bolund L *et al*: **WEGO: a web tool for plotting GO annotations**. *Nucleic Acids Research* 2006, **34**(Web Server issue):W293-W297.

77. Cooper GM: **The Cell, 2nd edition. A Molecular Approach**. Sunderland (MA): Sinauer Associates; 2000.

78. Sulpice R, Trenkamp S, Steinfath M, Usadel B, Gibon Y, Witucka-Wall H, Pyl E-T, Tschoep H, Steinhauser MC, Guenther M *et al*: **Network Analysis of Enzyme Activities and Metabolite Levels and Their Relationship to Biomass in a Large Panel of -*Arabidopsis* Accessions**. *The Plant Cell* 2010, **22**(8):2872-2893.

79. Hamilton JP, Robin Buell C: **Advances in plant genome sequencing**. *The Plant Journal* 2012, **70**(1):177-190.
80. Barthelson R, McFarlin AJ, Rounsley SD, Young S: **Plantagora: Modeling Whole Genome Sequencing and Assembly of Plant Genomes**. *PLOS ONE* 2011, **6**(12):e28436.
81. Hellsten U, Wright KM, Jenkins J, Shu S, Yuan Y, Wessler SR, Schmutz J, Willis JH, Rokhsar DS: **Fine-scale variation in meiotic recombination in Mimulus inferred from population shotgun sequencing**. *Proceedings of the National Academy of Sciences of the United States of America* 2013, **110**(48):19478-19482.
82. Cruz F, Julca I, Gómez-Garrido J, Loska D, Marcet-Houben M, Cano E, Galán B, Frias L, Ribeca P, Derdak S *et al*: **Genome sequence of the olive tree, Olea europaea**. *GigaScience* 2016, **5**(1):29.
83. Plomion C, Aury JM, Amselem J, Alaeitabar T, Barbe V, Belser C, Berges H, Bodenes C, Boudet N, Boury C *et al*: **Decoding the oak genome: public release of sequence data, assembly, annotation and publication strategies**. *Molecular ecology resources* 2016, **16**(1):254-265.
84. Park B-S, Lee H-K, Lee S-E, Piao X-L, Takeoka G, Wong R, Ahn Y-J, Kim J-H: **Antibacterial activity of Tabebuia impetiginosa Martius ex DC (Taheebo) against Helicobacter pylori**. *Journal of Ethnopharmacology* 2006, **105**(1-2):255-262.
85. Gómez Castellanos R, Prieto J, Heinrich M: **Red Lapacho (Tabebuia impetiginosa)—A global ethnopharmacological commodity?** *Journal of Ethnopharmacology* 2009, **121**(1):1-13.
86. Byeon S, Chung J, Lee Y, Kim B, Kim K, Cho J: **In vitro and in vivo anti-inflammatory effects of taheebo, a water extract from the inner bark of Tabebuia avellanedae**. *Journal of Ethnopharmacology* 2008, **119**(1):145-152.
87. Koyama J, Morita I, Tagahara K, Hirai K-I: **Cyclopentene dialdehydes from Tabebuia impetiginosa**. *Phytochemistry* 2000, **53**(8):869-872.
88. Hussain H, Krohn K, Ahmad VU, Miana GA, Green IR: **Lapachol: An overview**. *Arkivoc* 2007, **2007**(2):145.
89. Widhalm J, Rhodes D: **Biosynthesis and molecular actions of specialized 1,4-naphthoquinone natural products produced by horticultural plants**. *Horticulture Research* 2016, **3**:16046.
90. Romagnoli M, Segoloni E, Luna M, Margaritelli A, Gatti M, Santamaria U, Vinciguerra V: **Wood colour in Lapacho (Tabebuia serratifolia): chemical composition and industrial implications**. *Wood Science and Technology* 2013, **47**(4):701-716.
91. Alkan C, Sajjadian S, Eichler EE: **Limitations of next-generation genome sequence assembly**. *Nat Meth* 2011, **8**(1):61-65.
92 Silva-Junior OB, Grattapaglia D, Novaes E, Collevatti RG. **Supporting data for "Genome assembly of the pink Ipê (Handroanthus impetiginosus, Bignoniaceae), a highly-valued ecologically keystone Neotropical timber forest tree"**. *GigaScience* database 2017. http://dx.doi.org/10.5524/100379

Table 1. *Handroanthus impetiginosus* genome assembly statistics. The final assembly for each step contains scaffolds of length 1 kbp or longer.

Scaffold sequences	Allpaths-LG	Allpaths-LG/ Sspace/GapClose	Allpaths-LG/Sspace/ GapClose/Reapr
**Number**	57,815	16,090	13,206
**Total size, without gaps (bp)**	469,049,393	565,959,143	476,867,120
**Total size, with gaps (bp)**	614,626,609	586,542,612	503,314,177
**Number > 10 Kbp**	10,029	8,602	8,348
**Number > 20 Kbp**	6,920	6,791	6,647
**Number > 100 Kbp**	1,100	1,709	1,304
**Number > 1 Mbp**	2	0	0
**Longest sequence (bp)**	1,844,569	979,053	558,523
**Average size (bp)**	10,631	36,454	38,112
**N50 length (bp)**	57,726	97,266	80,946
**L50 count**	2,595	1,792	1,906
**GC %**	33.63	33.57	33.62

Table 2. *Handroanthus impetiginosus* gene prediction statistics with respect to the number, length and base composition of genes, transcripts, exons and introns.

	Genes	Transcripts	Exons	Introns
**Number**	31,688	35,479	154,209	122,521
**Average number/gene**	-	1.12	4.87	3.87
**Average length**	3,129	3,342	285	445
**N50 length**	4,421	4,643	477	839
**%GC**	38.38	38.22	42.60	32.83
**%N**	0.43	0.43	0.00	0.29

Table 3. The distribution of the minimal introns (53–125 bp) and the minimal-intron-containing genes – as the number of genes with at least one minimal intron – from selected plant species in comparison to the *H. impetiginosus* genome assembly.

**Species**	Genome size (Mbp)	Number of intron (bp)	Mean intron length (bp)	Minimal intron (%)	Gene (%)
***A. thaliana* (Rosids)**	120	118,037	164	72.29	57.08
***E. guttata* (Asterids)**	312	117,507	290	47.75	57.63
***P. trichocarpa* (Rosids)**	423	166,809	380	36.96	53.41
***E. grandis* (Rosids)**	691	137,329	425	33.49	48.38
***S. indicum* (Asterids)**	354	101,313	439	38.14	49.76
***H. impetiginosus* (Asterids)**	557	122,521	445	34.36	49.78
***S. lycopersicum* (Asterids)**	900	125,750	543	36.09	47.78

Figure Legends

Figure 1. The *Handroanthus impetiginosus* (Mart. ex DC.) Mattos (syn. *Tabebuia impetiginosa*, Bignoniaceae), tree UFG-1 whose genome was sequenced.

Figure 2. Depth of coverage analysis. (A) Histograms of k-mer frequencies in the filtered read data for k = 25 (red) and GenomeScope modeling equation on *H. impetiginosus* (blue). The x-axis shows the number of times a k-mer occurred (coverage). The vertical dashed dark blue lines correspond to the mean coverage values for unique heterozygous k-mers (left peak) and unique homozygous k-mers (right peak). (B) Density plot of read depth based on mapping all short fragment reads back to the assembled scaffolds (red). Left peak (at depth = 34x) corresponds to regions where the assembler created two distinct scaffolds from divergent putative haplotypes. The right peak (at depth = 67x) contains scaffolds from regions where the genome is less variable, allowing the assembler to construct a single contig combining homologue sequences. Histograms of Poisson modeling for read depth in the assembly (green, lambda = 34; blue, lambda = 67) are shown.

Figure 3. Depth of coverage analysis for the haplotype-reduced assembly. (A) Density plot of read depth based on mapping all short fragment reads back to the haplotype-reduced assembled sequences after identification and removal of redundant sequences due the structural heterozygosity in the genome. (B) Density plot for average sequencing coverage per-scaffold on the final assembly. The observed number of scaffolds in the final haplotype-reduced assembly and the respective read coverage (blue line) is shown in comparison to a Poisson process approximation (red line) with lambda = 63x, the observed average sequencing coverage in the useful read data.

Figure 4. Repeat content of the *H. impetiginosus* genome assembly. (A) The density of interspersed and tandem repeat as percent of the assembly. The size of the circles represents the number of copies in the assembly for each family of repeats; (B) Distribution of sizes of the consensus sequences for repeat families identified using *de novo* and homology methods for repeat characterization.

Figure 5. Transcriptome quality assessment (A) similarity search of *H. impetiginosus* putative peptides against source database of plant protein sequences using BLASTP algorithm (e-value 1e-6). Transcript count means the number of peptides of *H. impetiginosus* with best hit against the source database using bit-score and grouping results by taxon name. Transcript score corresponds to the average bit-score overall hits for each group using the best hit. We ordered taxon groups by their average bit-score overall hits and used Welch's t-test to compare the distributions of bit-score hits between two adjacent groups with p-values <0.01 (ns = non-significant; *** significant); (B) Completeness of the expected gene space of the genome assembly, estimated with BUSCO. The estimates were compared with genome annotations for other lamids, *Erythranthe guttata* and *Olea europaea*.

Figure 6. Contig termini analysis to investigate the possible genomic features associated with gaps in the genome assembly. Contigs were created from the genome assembly with the "cutN -n 1" command from seqtk program, which cut at each gap (of at least one basepair, i.e. one or more Ns). The figure shows the percentage of contig termini (the position of the terminal nucleotides of each contig) intersecting with different annotations of the genome.

Figure 7. Genes of the biosynthetic pathway of specialized quinoids. O-succinylbenzoate (OSB) pathway depicting the number of *H. impetiginosus* (Himp) annotated genes for the known enzymes that lead to the biosynthesis of the naphthoquinones, including lapachol. For comparison, it also shows the numbers of genes for the closely related *Mimulus guttatus* (Mgut), *Solanum lycopersicum* (Slyc), for the model *Arabidopsis thaliana* (Ath), and for the tree species *Eucalyptus grandis* (Egr) and *Populus trichocarpa* (Potri). The pathway was modified from [89].

Supplementary material

Table S1. Summary of the sequence data generated for the genome assembly of *Handroanthus impetiginosus* based on the ALLPATHS-LG algorithm.

Figure S1. Flow cytometry results of the sequenced tree UFG-1 of *H. impetiginosus*. Flow cytometry estimate of the nuclear DNA content was carried out using young leaf tissue on a BD

Accuri™ C6 Plus personal flow cytometer. *Pisum sativum* (genome size 9.09 pg/2C or ~4380 Mb/1C) was used as standard for comparison (M2). The estimate of nuclear DNA content for *H. impetiginosus* (M1) averaged over 10 readings was 1.155 pg/2C or 557.3 ± 39 Mb/1C.

Figure S2. Overview of the analytical pipeline with the bioinformatics steps and tools employed for genome (black arrows) and transcriptome assembly (red arrows), and for gene prediction and annotation (blue arrows). Bioinformatics programs are indicated in italic, blue, and the main file formats in red. The input sequences are highlighted in yellow boxes and the main products in green.

Figure S3. Distribution and characterization of simple sequence repeats in Handroanthus impetiginosus genome (A) Histogram of different motifs ranging from 1 to 6 bp (B) Distribution of the simple sequence repeats length detected in the genome assembly.

Figure S4. Comparison of the gene features parameters, such as number and length, between *H. impetiginosus* and the other selected dicot plant across distinct lineages of Rosids (*A. thaliana* and *P. trichocarpa*) and Asterids (*E. guttata* and *S. lycopersicum*). Frequency histograms are shown according to the whole-genome gene content annotation for (A) the complete predicted gene structure (B) exons and (C) introns. Dashed vertical lines are the average lengths for the gene features.

Figure S5. Histograms for Gene Ontology broader term annotations in the *H. impetiginosus* genome assembly. Terms for the Biological Process ontology were summarized with WEGO by the second tree level setting. The Pearson Chi-Square test was applied to indicate significant relationships between *H. impetiginosus* and the lamid *Erythranthe guttata* regarding the number of genes (at $\alpha \geq 5\%$). (A) Terms displaying remarkable relationship between the two datasets; (B) terms with a significant difference between the two datasets.

Figure S6. Same as Figure S6 but showing comparison between numbers of genes assigned to GO broader terms for *H. impetiginosus* and the lamid *Olea europaea*.

Figure S7. Sequence length distribution from the assemblies of *H. impetiginosus* and other two highly heterozygous trees of the genus *Quercus*. Figure shows density plots for the size of scaffolds with 2 kbp or longer in the three assemblies. Contigs metrics were computed by cutting at each gap (of at least 25 base pair, i.e. 25 or more Ns). Scaffolds and contigs length were plotted using the common logarithm to respond to skewness towards large values.

File S1. Evidences adopted to support protein-coding loci identification and assignment in the *H.impetiginosus* genome assembly. Two qualifiers – high-confidence and low-confidence – were added to the locus based on the reported evidences.

File S2. Genome assembly metrics from the assemblies of *H. impetiginosus* and other two highly heterozygous trees of the genus Quercus. Comparison between metrics based on the assemblathon_stats script part of the assemblathon2-analysis package (https://github.com/ucdavis-bioinformatics/assemblathon2-analysis). Metrics were computed for scaffolds with 2 kbp or longer in length. Genomic sequences in scaffolds for *Quercus lobata* was obtained from https://valleyoak.ucla.edu/genomicresources/ (accessed on 9/20/2017). For *Quercus rubra*, genomic sequences in scaffolds were downloaded from the ENA (European Nucleotide Archive) repository, accessions LN776247-LN794156.

Figure 1 Click here to download Figure Figure_Main_1.jpg

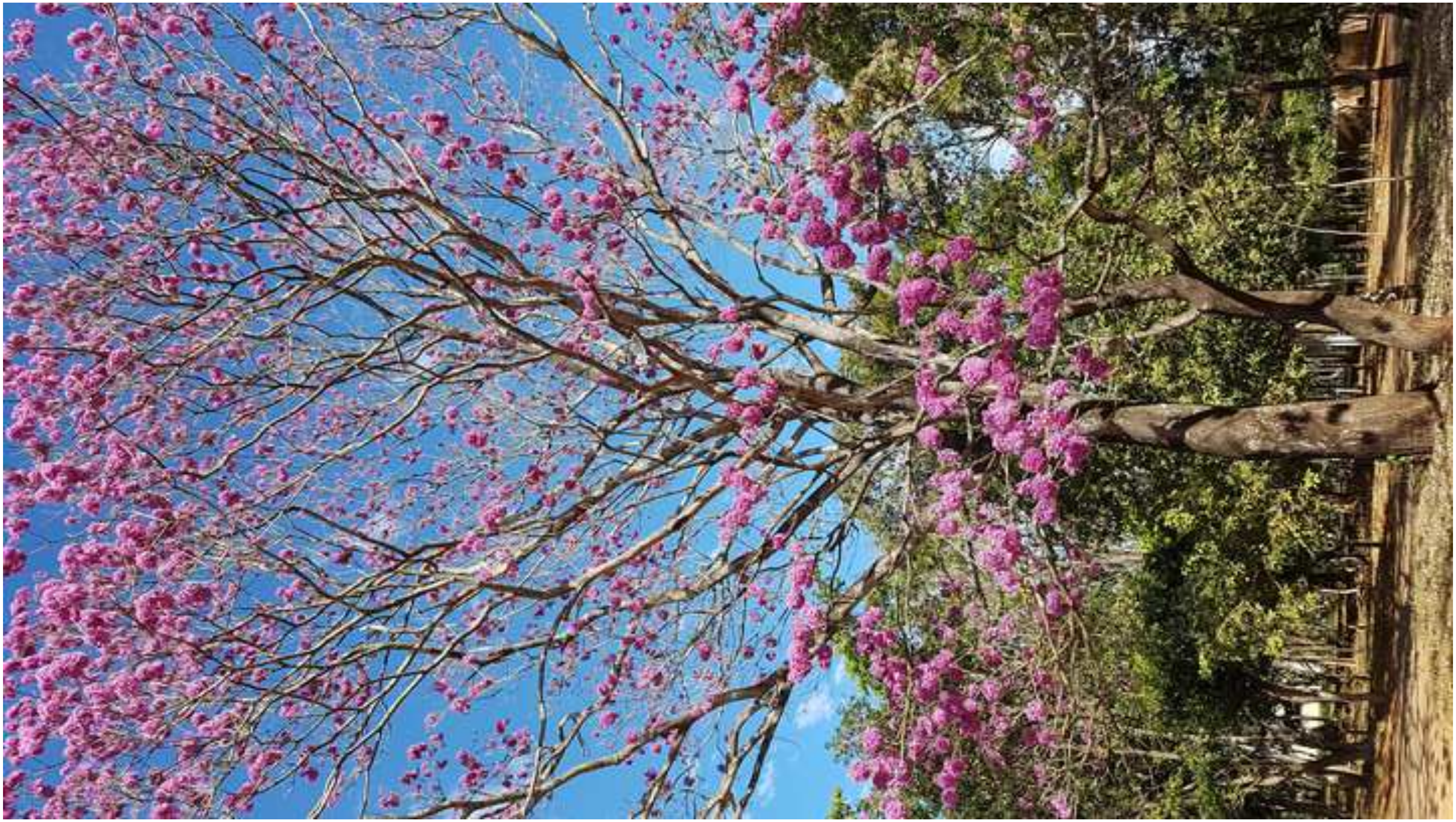

Figure 2

Click here to download Figure
Figure_Main_1.pdf

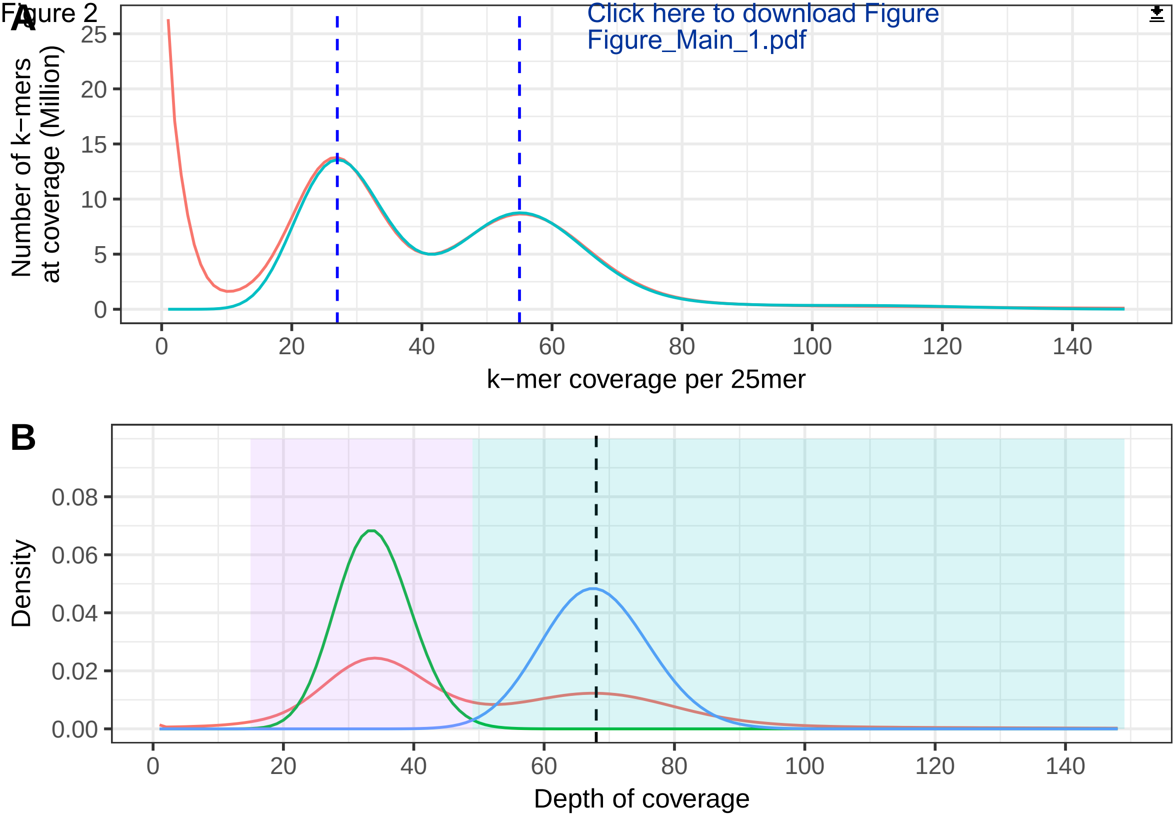

Figure 3

Click here to download Figure Figure_Main_2.pdf

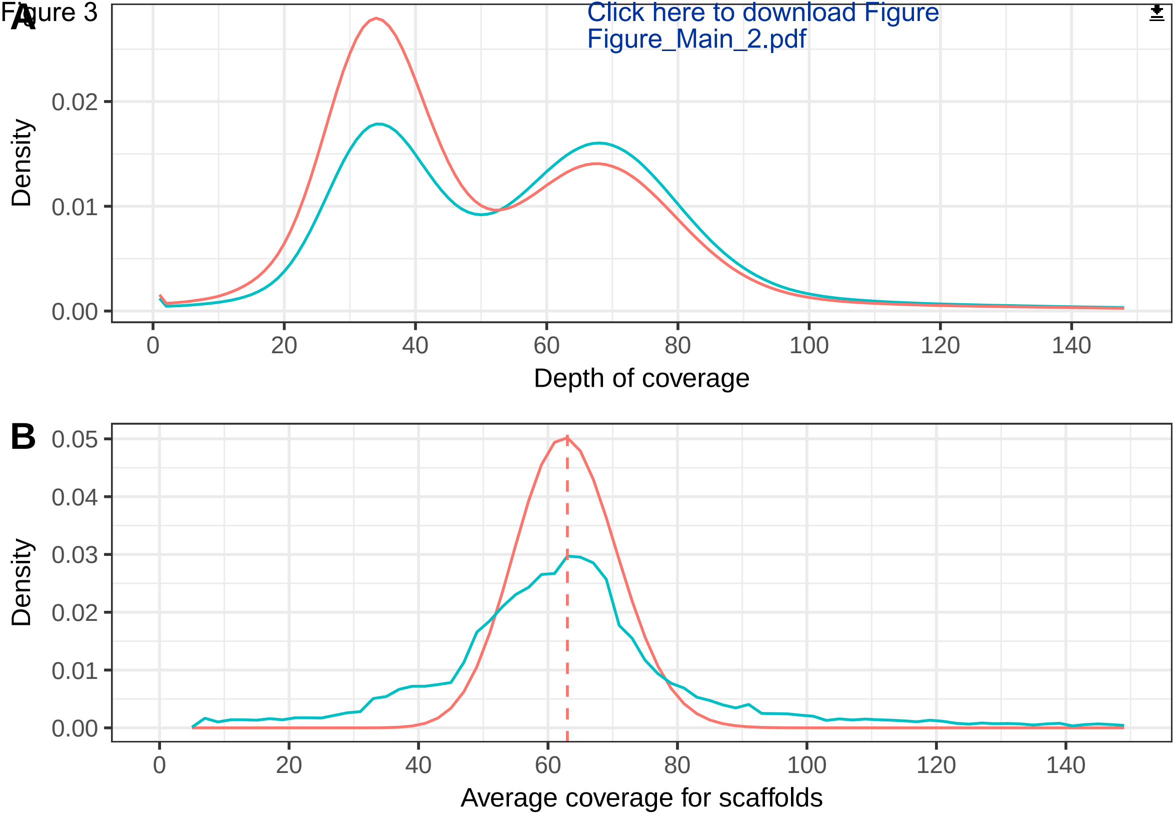

Figure 4

Click here to download Figure Figure_Main_3.pdf

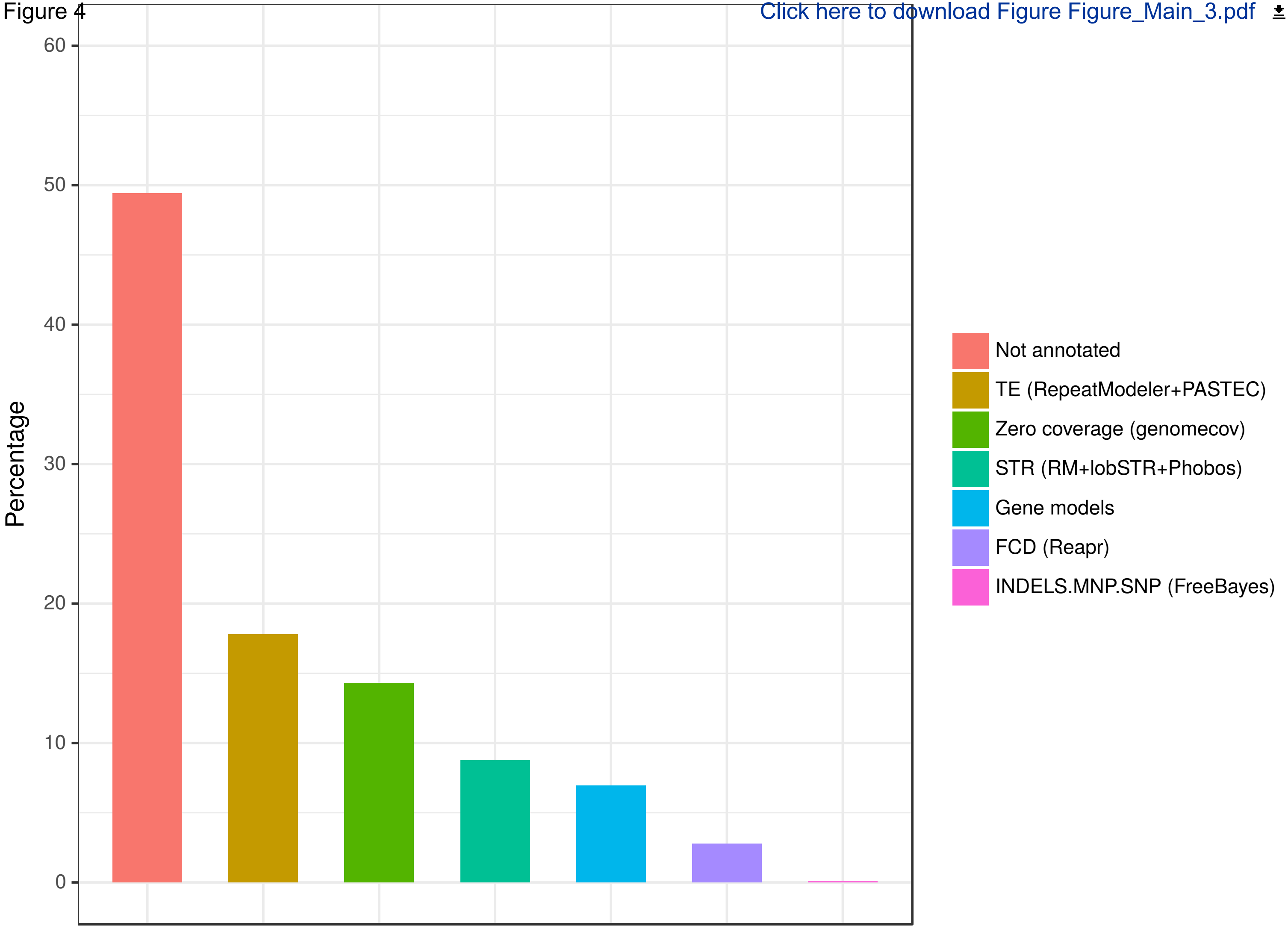

Figure 5

Click here to download Figure Figure_Main_4.pdf

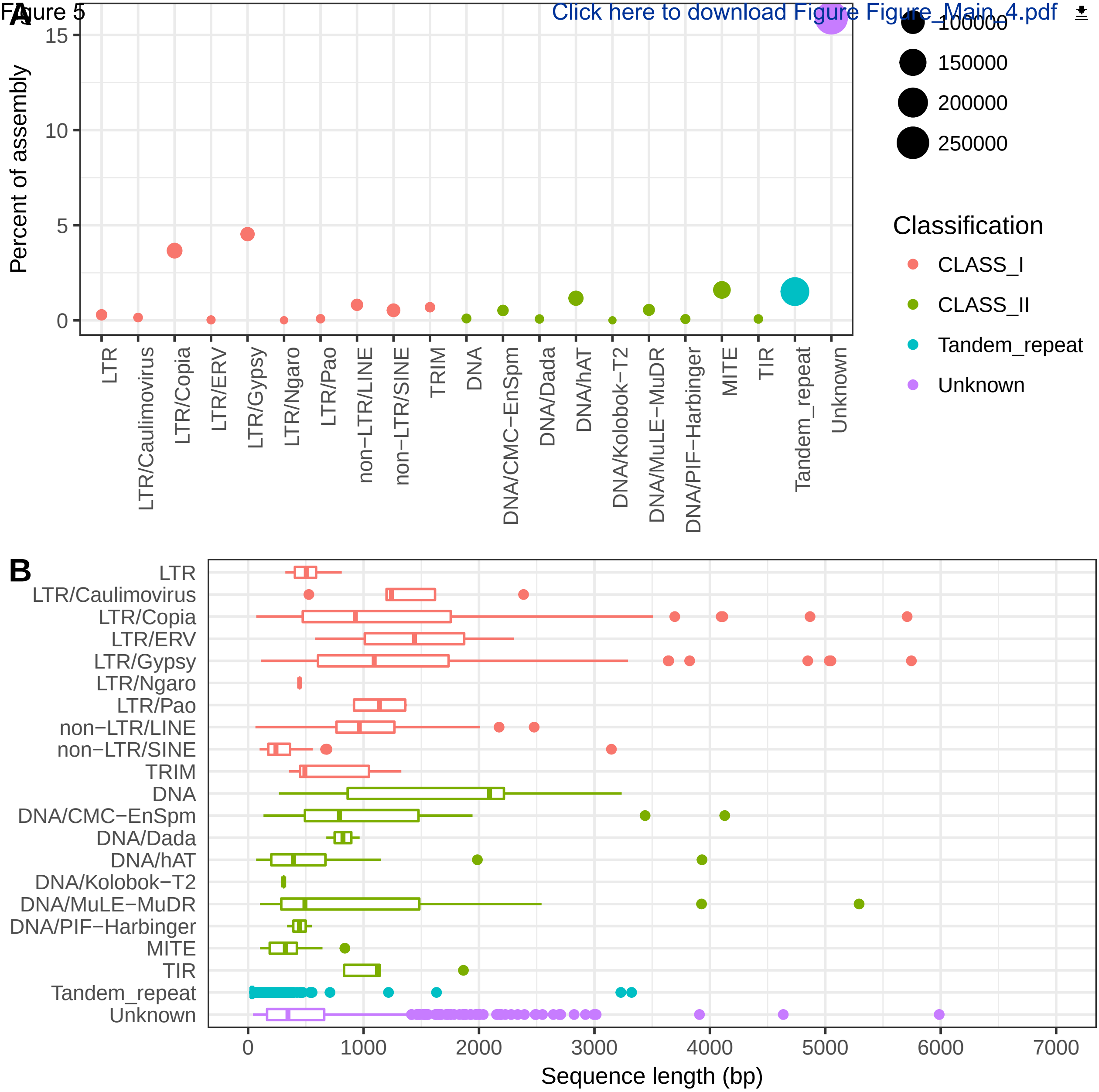

Figure 6 Click here to download Figure Figure_Main_5.pdf

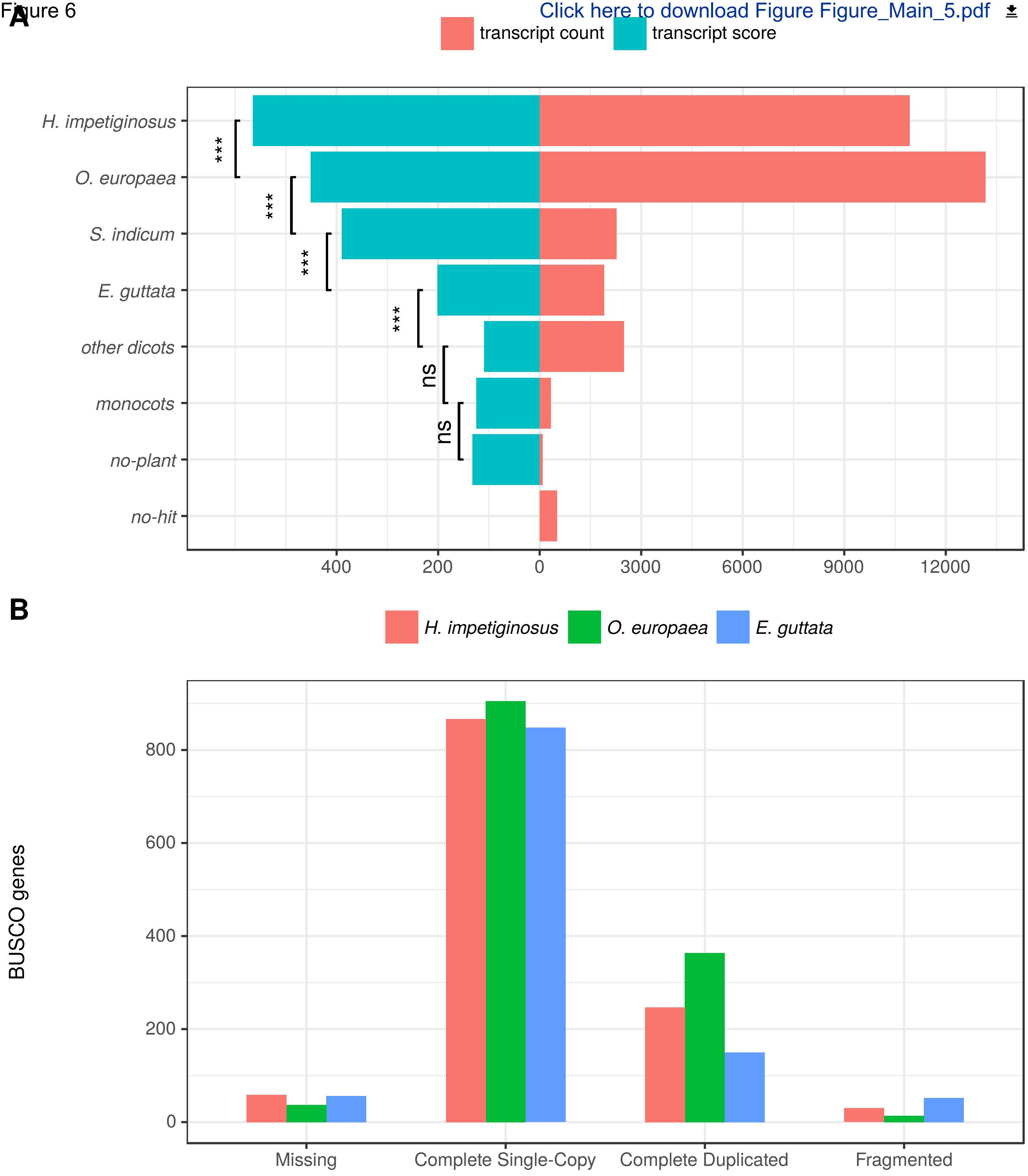

Figure 7

Click here to download Figure Figure_Main_6.pdf

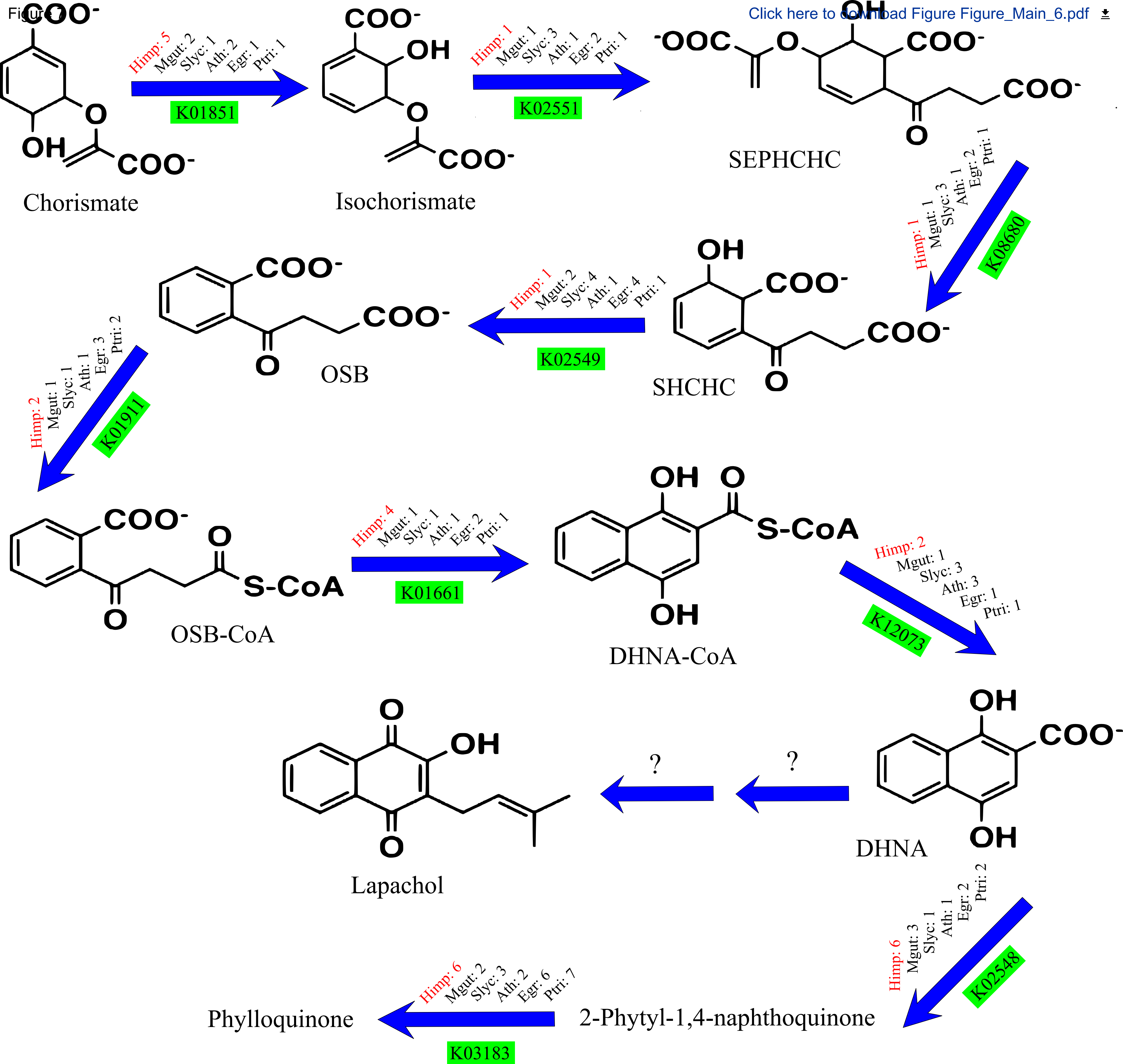

Click here to access/download
Supplementary Material
Supp_Material_H.impetiginosus_genome_GIGA-D-17-00159.R2.docx

Click here to access/download
Supplementary Material
Supplementary_FileS1.xlsx

Supplementary Material S2

Click here to access/download

Supplementary Material

Supplementary_FileS2.xlsx
