# Supplementary material for: Genome assembly of the Pink Ipê (Handroanthus impetiginosus, Bignoniaceae), a highly valued, ecologically keystone Neotropical timber forest tree
Source: Gigascience. 2017 Dec 13;7(1):gix125. doi: 10.1093/gigascience/gix125 (PMC5905499; doi:10.1093/gigascience/gix125)
Supplement: Supplemental material [file gix125_supp.zip › Supp_Material_H.impetiginosus_genome_GIGA-D-17-00159.R2.docx]

**Supplementary Material to:**

**Genome assembly of the Pink Ipê *(Handroanthus impetiginosus*, Bignoniaceae),**

**a highly-valued ecologically keystone Neotropical timber forest tree**

Orzenil Bonfim da Silva-Junior^1,2^, Dario Grattapaglia^1,2^, Evandro Novaes^3^, Rosane G. Collevatti^4*^

**Table S1.** Summary of the sequence data generated for the genome assembly of *Handroanthus impetiginosus* based on the ALLPATHS-LG algorithm.

| **Platform** | **Library type** | **DNA Fragment**  **Expected size** | **Number of raw reads** |  | **ALLPATHS-LG** | | |
| --- | --- | --- | --- | --- | --- | --- | --- |
|  |  |  |  | **Number of useful reads** | **Estimated sequence coverage (×)** | **DNA Fragment observed size (bp)** | **Estimated physical coverage (×)** |
| HiSeq2500 PE 150 | fragments | (170 – 380) bp | 185,259,378 | 125,890,406 | 38.0 | -99 ± 50 | 39.1 |
| HiSeq2500 PE 150 | fragments | (500 – 700) bp | 116,411,024 | 72,390,668 | 25.1 | 38 ± 120 | 50.7 |
| HiSeq2000 PE 100 | Mate pair | (4 – 5) kbp | 97,876,408 | 18,990,964 | 10.1 | 4126 ± 476 | 143.2 |
| HiSeq2000 PE 100 | Mate pair | (8kb – 10) kbp | 107,929,932 | 9,767,840 | 7.5 | 8156 ± 684 | 152.5 |
| HiSeq2000 PE 100 | Mate pair | (15 – 20) kbp | 91,561,590 | 1,791,712 | 1.5 | 3164 ± 2094 | 16.2 |
| **Total reads / bp** |  |  | **599,038,332** | **228,831,590** | **82** |  | **400.8** |


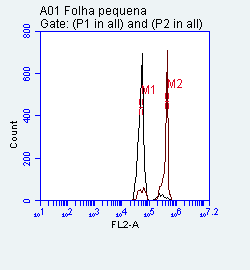


**Figure S1.** Flow cytometry results of the sequenced tree UFG-1 of *Handroanthus impetiginosus*. Flow cytometry estimate of the nuclear DNA content was carried out using young leaf tissue on a BD Accuri™ C6 Plus personal flow cytometer. *Pisum sativum* (genome size 9.09 pg/2C or ~4380 Mb/1C) was used as standard for comparison (M2). The estimate of nuclear DNA content for *Handroanthus impetiginosus* (M1) averaged over 10 readings was 1.155 pg/2C or 557.3 ± 39 Mb/1C.

**Figure S2.** Overview of the analytical pipeline with the bioinformatics steps and tools employed for genome (black arrows) and transcriptome assembly (red arrows), and for gene prediction and annotation (blue arrows). Bioinformatics programs are indicated in italic, blue, and the main file formats in red. The input sequences are highlighted in yellow boxes and the main products in green.

**Figure S3.** Distribution and characterization of simple sequence repeats in the *Handroanthus impetiginosus* genome. (A) Histogram of different motifs ranging from 1 to 6 bp (B) Distribution of the simple sequence repeats length detected in the genome assembly.

**Figure S4.** Comparison of the gene features parameters, such as number and length, between *H. impetiginosus* and the other selected dicot plant across distinct lineages of Rosids (*A. thaliana* and *P. trichocarpa*) and Asterids (*E. guttata* and *S. lycopersicum*). Frequency histograms are shown according to the whole-genome gene content annotation for (A) the complete predicted gene structure (B) exons and (C) introns. Dashed vertical lines are the average lengths for the gene features.

**Figure S5.** Histograms for Gene Ontology broader term annotations in the *H. impetiginosus* genome assembly. Terms for the Biological Process ontology were summarized with WEGO using the second tree level setting. A Pearson Chi-Square test was applied to indicate significant relationships between *H. impetiginosus* and the lamid *Erythranthe guttata* regarding the proportion of the numbers of genes (at alpha ≥ 5%). (A) GO terms that display remarkable relationship between the two datasets; (B) GO terms with a significant difference between the two datasets.

**Figure S6.** Same as Figure S6 but showing comparison between numbers of genes assigned to GO broader terms for *H. impetiginosus* and the lamid *Olea europaea*.

**Figure S7.** Sequence length distribution of the assemblies of *H. impetiginosus* and other two highly heterozygous trees of the genus *Quercus*. Density plots for the size of scaffolds with 2 kbp or longer in the three assemblies are shown. Contigs metrics were computed by cutting at each gap (of at least 25 base pair, i.e. 25 or more Ns). Scaffolds and contigs lengths were plotted using the common logarithm to respond to skewness towards large values.
