# Supplementary material for: Genome assembly of the Pink Ipê (Handroanthus impetiginosus, Bignoniaceae), a highly valued, ecologically keystone Neotropical timber forest tree
Source: Gigascience. 2017 Dec 13;7(1):gix125. doi: 10.1093/gigascience/gix125 (PMC5905499; doi:10.1093/gigascience/gix125)
Supplement: Response_to_Reviewer_Comments_Original_Submission.pdf [file gix125_response_to_reviewer_comments_original_submission.pdf]

Author's Response To Reviewer Comments

Goiânia, 27 September 2017

Professor Hans Zauner
Assistant Editor
GigaScience

Dear Prof. Zauner,

Enclosed you will find the manuscript GIGA-D-17-00159-R1, the revised version of the originally submitted one entitled “Genome assembly of the pink Ipê (Handroanthus impetiginosus, Bignoniaceae), a highly-valued ecologically keystone Neotropical timber forest tree” that we are re-submitting for publication in GigaScience as Data Note.
Thank you for the opportunity to revise our manuscript. We have responded in great detail to all comments and suggestions made by the reviewers in the text following this letter. We sincerely appreciated the reviewers’ suggestions that really helped us improving the manuscript and we hope that all the issues raised were addressed at satisfaction.
We reiterate our confidence that this work opens exceptional opportunities to empower molecular studies of the several species of the Tabebuia Alliance based on useful genomic resources for genetic and functional analysis in the species. In the revised version we added more data and analyses to show that this read set, genome assembly and corresponding annotations are consistent contributions to the genomics community. Their availability should inspire the development of similar genomic resources for the still largely neglected forest trees of the mega-diverse tropical biomes.
To facilitate the visualization of how we have dealt with each single point, we have reiterated each concern or suggestion made by the reviewer in bold and placed our reply (RESPONSE:) immediately after, in the section following this cover letter, the entire document entitled “Cover Letter+Responses_to_GigaSience MS GIGA-D-17-00159-R1”. Hope you and reviewers will consider this as a more acceptable version for publication in GigaScience

Thank you for your consideration. We look forward to hearing from you.

Sincerely yours,
Rosane Collevatti (on behalf of all co-authors)
Laboratório de Genética & Biodiversidade, Instituto de Ciências Biológicas,
Universidade Federal de Goiás, CP 131, 74001-970, Goiânia, GO, Brasil.
E-mail: rosanegc68@hotmail.com. Phone: +55 62 3521-1729

Responses to Reviewer #1
The main problem I have is not being able to access the data on NCBI. I can see two biosamples and a bioproject, but I cannot find the SRA records, the genome or the annotations. The biosample record page will usually have a link at the bottom to the SRA, but this is missing (both

only link back to the bioproject). Further if you search by the taxonomic name of the tree through Entrez, the number of records for genome is 0 and for SRA is also 0. Possibly the authors are waiting for final release until this is published? But this definitely needs to be taken care of prior to publication.
RESPONSE: The sequence data has been deposited in the SRA of NCBI and are available through the following accession identifiers:
1. Study: whole-genome sequencing
1.1 Sample name: Himp-UFG1
1.2 BioSample: SAMN05195323
1.3 SRA: SRS1483442; Run accessions (5): SRR3624821 - SRR3624825
2. Study: mRNA-Seq
2.1 Sample name: HIMP-UFG1-RNA-POOL
2.2 BioSample: SAMN07346903
2.3 SRA: SRS2349699; Run accession (1): SRR5820886
3. Genome assembly: this Whole Genome Shotgun project has been deposited at DDBJ/ENA/GenBank under the accession NKXS00000000. The version described in this paper is version NKXS01000000.
Lines 91-92 "beyond a relatively small numbers of microsatellites with their caveats for more sophisticated genetic analyses." Numbers should be number and also clarification of what the word caveats is referring to.
RESPONSE: We clarified the sentence and added the number of microsatellites available before our study was developed and the corresponding references. We also added a reference regarding the well known caveats of microsatellites for evolutionary analyses. The sentence now reads: “The species has virtually no genomic tools and resources, beyond a handful of 21 microsatellites [17] with their caveats for more sophisticated genetic analyses in the areas of population structure, admixture, and effective population size and evolution [18].”
Lines 112-113 - mention of other species being sequenced, but this isn't addressed in the results. Should remove from methods if these results are not addressed in this manuscript
RESPONSE: Thanks. We removed the sentence. In fact, this pooled sample was used in a second study that addressed SNP discovery, which is the subject of a separate manuscript.
Line 115 - Min should be Mini
RESPONSE: Corrected
Line 123 - I'm not sure if "jump" is the official word or just jargon, maybe consider "fragment lengths of"
RESPONSE: Actually, the jargon “jump” is used for such mate-pair library. However we agree that “fragment lengths of” reads better. We changed accordingly.

Line 133 - mention of a "perl script" - this needs to be made available through github or somewhere else
RESPONSE: The perl script TrimAdaptor.pl was provided to our group by its authors at the High-Throughput Sequencing and Genotyping Center Unit of the University of Illinois Urbana-Champaign. We added this information in the section “AVAILABILITY OF SUPPORTING DATA”. The script was uploaded to the GigaScience’s data repository following the permission of the original authors.
Line 139 - same comment about "jumping" - I would consider changing to "mate pair"
RESPONSE: Changed to mate-pair

Line 402 - "showed" to something else, perhaps "illustrated"
RESPONSE: Changed to “depicted”.
Line 432 - adequately doesn't really make sense in this sentence, perhaps remove
RESPONSE: Agreed. Removed.
Line 439 - The citation to Fig S6A doesn't make much sense, the text refers to blast results but the figure shows Gene ontology terms (and the figure is cited later in the GO section), maybe a supplemental figure is missing?
RESPONSE:
We corrected the figure Fig S6A to better convey the result presented. The figure regarding gene ontology terms analysis is now depicted as Fig S7.
Line 477 – It’s not clear how the search for the genes was done (BLAST?)
RESPONSE: We clarified this in the manuscript by adding the marked phrases below:
"Given their medicinal and biological relevance, we have searched the H. impetiginosus annotated genes for the enzymes involved in the biosynthesis of naphthoquinones. By searching for the KEGG identifiers of these enzymes (e.g. K01851) in the InterPro annotation results, we have found all the important known enzymes that lead to the biosynthesis of lapachol (Fig. 6). Unfortunately, however, the last two steps of the lapachol biosynthesis pathway still constitute unidentified enzymes [79]. For comparative purposes, we downloaded the annotation file of five other species from the Phytozome version 11 (Fig. 6). The number of H. impetiginosus genes encoding for the enzymes of each step in the pathway is comparable to the numbers found in other species."

Responses to Reviewer #2
The article describes the genome assembly of Handroanthus impetiginosus, a neotropical timber tree. Because of heterozygosity in the diploid genome that was sequenced, the final assembly is fragmented (N50=81,316bp, L50=1906). The assembly fragmentation might be an issue for future analyses, and the authors should be more specific about that. Some parts of the text should be rewritten in order to acknowledge the fact that the assembly obtained is highly fragmented. For instance, the sentence (line 337) "Our genome assembly metrics are similar to recent reports of genome assemblies of other highly heterozygous forest tree genomes", should be discarded for two reasons: first, if other heterozygous genomes were assembled in a highly fragmented way, the authors should not be satisfied with doing "as bad", but should aim at doing better. Second, the metrics obtained for Quercus robur were actually better that those obtained for pink Ipê (N50=260kb, L50=1468)
RESPONSE: We have looked at these issues in depth and revised our manuscript accordingly. We agree with the reviewer that we need to be more specific about the limitation of the assembly for further analysis. We disagree, however, with the consideration that the assembly fragmentation might be an issue for future analysis. At most, it might be an issue for comparative genomics analysis and studies of genome evolution, but this is a well-known limitation of unfinished de novo assembly generated primarily with short reads like this one (see Alkan et al. 2011. Limitations of next-generation genome sequence assembly. Nature Methods. doi: 10.1038/nmeth.1527). Evolutionary issues and large-scale comparative genomic are beyond the scope of our manuscript. We added a word of caution to end users regarding this particular issue. It was not our intention or claims to provide a fully finished resource for the research community but only a quality level, well-curated, extensively supervised assembly. We have described in the manuscript that our main goal was to provide useful genomic resources for genetic and

functional analysis in the species (Lines 341-344; 420-422). Nevertheless, we admit that in parts of the manuscript we have suggested that these resources can empower evolutionary studies (Lines 56; 71; 502). Although we are confident that such studies can be carried out using our assembly, at least at the gene-level of gene-family level, we did not validate it in this present research. We thus rewrote those sentences to tone down any suggestion about using this genome assembly for deeper evolutionary analysis.

Difficulties in obtaining a highly contiguous assembly for this highly repetitive and heterozygous genome was anticipated and discussed in the manuscript. Comparison provided with other similar assemblies was clarified. The N50 metric mentioned by the reviewer is however a misleading one as discussed elsewhere (See Bradnam et al. 2013 Assemblathon 2: evaluating de novo methods of genome assembly in three vertebrate species. DOI: 10.1186/2047-217X-2-10), but we do agree that we should have added more information in the manuscript regarding this comparison. Our brevity in this respect certainly contributed to the misinformed judgment about the quality of our assembly when the reviewer alluded that we "were satisfied with doing this genome assembly as bad". A careful and deeper comparison of our assembly with assemblies for other heterozygous trees clearly shows that our assembly is, in fact, better and particularly so when compared to the one for Quercus that the reviewer mentions. Please, find as additional supplementary material to the manuscript a new figure and a file that brings a detailed comparison of our assembly metrics with other three recently published assemblies for highly heterozygous trees (Figure S8 and File S2).

Certainly, there are still inaccuracies at the base and assembly level in this unfinished assembly but we tried hard not to deliver it to end users without an appropriate documentation, which make this initial read set and sequence a primary and very reliable object for further improvement. To clarify the main features of our assembly that highlight its quality and to better respond to the reviewer's suggestion, we also improved the Re-use Potential section.

Abstract, line 47: the terms "redundancy in the consensus determination" are not clear. Figure 2B shows that most scaffolds correspond to a consensus between the two haplotypes. What does "redundancy" means? Does it mean that for some parts of the genome the two haplotypes were assembled separately? The sentence should be clarified.

RESPONSE: Thanks for the remark. To complement the depth of read coverage analyses, we added a new analysis – contig termini analysis – carried out to identify the most probable causes of breaks in the assembly. Contig termini are the positions of the terminal nucleotides of each contig from the genome assembly created by cutting at each gap (of at least one base pair, i.e. one or more Ns). This analysis was developed using a protocol described elsewhere (Tørresen et al. 2017. BMC Genomics 2017, 18(1):95). As noted by the reviewer, figure 2B shows that most scaffolds correspond to a consensus between the two haplotypes. The contig termini analysis reinforces this view showing that virtually none of the breaks are caused by allelic variants that could arise from splitting contigs of divergent haplotypes within a locus. Thus, there is no redundancy in the assembly in the sense that the two haplotypes might have been assembled separately. We removed this sentence in the manuscript.

Line 156: Figure S1 is called but it does not correspond to the pipeline (it should be figure S3). Along the manuscript numerous calls to figures do not correspond to the actual list of figures: the numbering and calling of figures should be checked carefully.

RESPONSE: All the figure and table numbers both in the main text and supplementary material were checked carefully and corrected where necessary.

Line 262 (316, table 1): The metrics (N50, L50…) are most of the time given for the whole

assembly and for sequences longer than 20kb. What is the size of the smaller scaffolds in the assembly (was a threshold set)? It would be interesting to provide metrics (in table 1 also) for scaffolds >1kb or >2kb: how much of the genome is included in such scaffolds? Filtering out short scaffolds from the assembly should be envisaged, since no genes can be annotated in short scaffolds. It would have the advantage of providing better assembly metrics.
RESPONSE: We agree that filtering out short scaffolds is adequate. In fact, in our study, we have considered only scaffolds of length 1 kbp or longer in each step of the assembly process. We explicitly added this information to the manuscript where necessary. We added this phrase in the title of Table 1: "All assembly step only contain scaffolds of length 1 kbp or longer". We added the phase "of length 1 kbp or longer" in parts of the main manuscript in which we were describing the assembly metrics. We also added a supplementary table providing metrics of how much of the assembly is included in scaffolds of length 2 kbp or longer. To illustrate the quality of our assembly we also included File S1 with a detailed comparison of our assembly with other recent assemblies for diploid, highly heterozygous tree genomes.
Line 285: the N50 is given for scaffolds >1kb, and not for all scaffolds: is it possible to provide the same metrics for all assemblies in order to be able to compare them?
RESPONSE: In our study, we discarded short scaffolds of length smaller than 1 kbp, and metrics were provided considering all scaffolds in the assembly. We rewrote the phrase as follows: "The final genome assembly after REAPR breaks had 19,319 sequences of length 1 kbp or longer, with 576,929,188 bp. N50 size of scaffolds dropped from 97 kbp (L50 = 1,792) to 71 kbp (L50 = 2,379).".

Lines 281-288: What proportion of the sequence-coverage differences called by REAPR correspond to boundaries between regions where alleles were assembled separately vs collapsed? If most of those errors are due to heterozygosity, would it make sense not to use the coverage information to break the scaffolds? Is there an option in REAPR in order to avoid the breaks? The procedure to filter out redundant copies of unmerged haplotypes might then require to split scaffolds, but it might result in less breaks in the assembly. Can the authors discuss on that?
RESPONSE: Thanks for pointing out these issues. We looked at these points in depth and our considerations follow below to provide some clarification. We did not say that "most errors are due to heterozygosity". In lines 274-279, we wrote that the most frequent error reported in the Reapr analysis (~92%) was caused by reads that mapped ambiguously to the assembly. It can be due to interspersed repeats with high-copy families, short-tandem repeats and low-copy repeats such as segmental duplications; all of them are known to be highly represented in eukaryotic genomes. These highly similar sequences reduce the depth of coverage reported for each base but do not imply in breaks of scaffolds unless the calculated fragment coverage distribution error (FCD) is higher than a theoretical value threshold. So, it is not the coverage information at each base that leads to a break but the FCD error at each base related to a background noise (the FCD error threshold). As correctly observed by the reviewer, it does not make sense to use the coverage information to break the scaffolds. Due to the variance in depth expected in the shotgun process and the presence of repeats, it could result in the introduction of large number of false breaks.
Our only intention in removing putative pairs of sequences that looked like haplotypes of the same locus possibly assembled separately, was to provide a reliable resource for further variant calling analysis. It was not this procedure that required to split scaffolds. The procedure to identify regions in the assembly that have fragmented distribution around bases causing an FCD

error was carried out to evaluate the overall and local accuracy of the assembly process and to prevent misassemblies. It should be a mandatory process for every de novo assembly due to the difficult decisions taken by different programs to resolve repeats and fuse contigs containing allelic variants in a unique consensus sequence. Of course, we do not advocate the use of FCD but any similar measure of accuracy of the process should be used. For instance, FRCurve measure implemented in Amosvalidate, which is included as part of the AMOS assembly package is another interesting alternative (Vezzi et al. Feature-by-Feature – Evaluating De Novo Sequence Assembly PLosOne (DOI: 10.1371/journal.pone.0031002). We have carried out this Reapr analysis just to eliminate fragrant misassemblies that could hinder further use of this genomic resource.
We added a new analysis in the manuscript (contig termini analysis) to detect the proportion of features with overlap to contigs end in the assembly. Contig termini are the positions of the terminal nucleotides of each contig from the genome assembly created by cutting at each gap (of at least one base pair, i.e. one or more Ns). This analysis was carried out using the protocol described elsewhere (Tørresen et al. 2017. BMC Genomics 2017, 18(1):95 doi: 10.1186/s12864-016-3448-x).
In the final assembly, the proportion of contig ends that overlap to breaks suggested by the FCD analysis using Reapr is very low (<3%), i.e. there are very few detected misassembles. Moreover, virtually none of contig ends overlap to allelic variants annotated with FreeBayes using read data mapped to the final genome assembly. Contig termini overlap most prominently (~50%) with regions that do not encompass any structural annotation in the assembly or regions that have no depth of coverage (~15%) based on mapped reads. It suggests that contig ends match large repeats not resolved given the short-read sequence data. Another possibility is that interruptions in continuity and contiguity might be due to young euchromatic segmental duplication with higher sequence similarity to the consensus sequence. This hypothesis is compelling given that the size of remaining gaps within scaffolds (2,384 ± 3,167) bp is not longer that the longest fragments used in the mate-pair libraries (>10 kbp). Moreover, the completeness of the assembly related to the empirically determinate genome size (90%) does not suggest that breaks of contiguity contain much longer sequences.

Lines 328, 332: Figure 1C and D are called but the described figures correspond to Figure 2A and B.
RESPONSE: Corrected
Line 348: know -> known
RESPONSE: Corrected
Line 352: Mostly -> Most
RESPONSE: Corrected
Line 356: "expand a wide range of sequence sizes" : the sentence should be corrected
RESPONSE: Corrected. It now reads: "… cover a wide range of sequence sizes from 42 bp…."
Line 361: "unknown non-classifed" sequences: are not the two terms redundant?
RESPONSE: Removed the word “unknown”
Line 366: Figure 2 is called, it should be Figure 3
RESPONSE: Corrected
Line 390: 31,668 genes were annotated. This number is relatively high for a plant genome. It would be interesting to explore paralogous gene clusters: are there duplicated genes in the genome? Are these genes more likely to have arisen from WGDs or tandem repeats? If such

analyses are possible despite the fragmented nature of the assembly, they would be of great interest.
RESPONSE: Actually, the number of genes annotated in the H. impetiginosus (~32k) can be considered quite average for a plant genome. For example, S. lycopersicum has ~34k protein coding genes, Populus 42K, Eucalyptus 36K and soybean 56K. Among other plants from the order Lamiales, monkey flower (M. guttatus) has 28k (Hellsten et al. 2013 PNAS 110:19478-19482) protein coding genes, sesame (S. indicum) has 23k (Zhang et al. 2013 Genome Biology 14:401) and olive tree (O. europaea), which is believed to have undergone whole-genome duplication (WGD) events, has 56k genes (Cruz et al. 2016 GigaScience 5:29). Nevertheless, we included in our manuscript an additional analysis to identify low-confidence set of genes based on signatures derived from loss of information due to the fragmentation of this unfinished genome assembly for H. impetiginosus. The results were summarized in a new Supplementary File S1 provided along with the main manuscript. If these low-confidence genes are excluded, the number of genes with reliable annotation in the genome assembly is 28,603, which is actually 90% of the total estimate of 31,668. This high-confidence subset contains approximately the same number of genes reported for other members of the Lamiales.
To account for gene families in the assembly, we explored the catalog of genes in H. impetiginosus by predicting orthologous groups (orthogroup) with OrthoFinder (Emms & Kelly 2015 Genome Biology 16:157). For comparative genomics, the orthology analyses was performed also with proteins from the basal plant A. trichopoda, the Lamiales M. guttatus and O. europaea., as well as the forest tree P. trichocarpa. The number of genes from each species was analyzed in each orthogroup. The percentage of orthogroups with only one gene from each species was higher in A. trichopoda, H. impetiginosus and M. guttatus, compared to the species (P. trichocarpa and O. europaea) that are known to have experienced WGD events. P. trichocarpa and O. europaea, on the other hand, present higher percentages of orthogroups with two or more genes (Figure 1). Given the fragmentation of the genome assembly and the lack of scaffolds mapping onto chromosomes, it is hard to analyze WGD events in the H. impetiginosus genome at relevant scale. Nevertheless, these orthology results provide initial evidence that this genome may have not undergone WGD events.

Figure 1. Distribution of the number of genes in orthogroups of the five species analyzed. Results indicate that H. impetiginosus has fewer genes in the orthogroups compared to the species O. europaea and P. trichocarpa that are known to have experienced recent WGD events.
We also investigated the possibility of tandem duplications in the H. impetiginosus genome by analyzing the genome location of genes from the same orthology groups. On average orthogroups with two or more genes presented 87.1% of these elements in different scaffolds. Two genes from the same scaffold were found in the same orthogroup in 10.1% of the cases, on average. The remaining 2.8% are instances where three or more genes from the same orthogroup were in the same scaffold. These results indicate that tandem duplication of genes may have not been frequent throughout the evolution of the H. impetiginosus genome. Even considering only scaffold co-localization, regardless of distance, our estimates are far from the high frequency of tandemly duplicated genes (~34%) observed in the Eucalyptus genome (Myburg et al. 2014) for example.
Line 439: Figure S6 -> Figure 4A ?
RESPONSE: Corrected
Line 446: Figure 3B -> Figure 4B?

RESPONSE: Corrected
Lines 451-452: BUSCO results were benchmarked using poplar. Poplar is known to have undergone a Whole Genome Duplication; Was the duplication status of Handroanthus investigated? If there was no WGD, the duplicate level is probably not comparable to that of Populus.
RESPONSE: Duplication status of H. impetiginosus was not specifically investigated. This was beyond the scope of this initial work. Following the reviewer's suggestion we have however benchmarked BUSCO results using other lamids, Erythranthe guttata and Olea europaea as described above.
Lines 454-464: GO terms were compared to those of poplar. Why was such a distant species chosen for the comparison? What about a comparison with other asterids, or even lamids (E. guttata, or Olea)?
RESPONSE: Thanks. Following the reviewer's suggestion we have performed GO terms comparison using other lamids, Erythranthe guttata and Olea europaea.
Lines 482-487: The authors report that some steps of the quinoid metabolism are encoded by more genes in pink Ipê than in other species. It would be interesting to elucidate how these genes were amplified in the Hydroanthus genome: is it possible to build a phylogeny of these genes ? Are some of them located on the same scaffold? (are they possibly deriving from tandem duplications?...)
RESPONSE: Analyses with OrthoFinder confirmed that many genes of each step of the quinoid pathway are indeed from the same orthologue group. We investigated whether genes from these quinoid orthogroups have arisen from tandem duplications. By analyzing if genes groups were in the same scaffold, we found little evidence of gene family expansion by tandem duplication. Of the eight quinoid orthogroups identified, containing 23 genes, in only one there were two genes localized in the same scaffold. With respect to a phylogenetic analyses of these genes, we believe that this is beyond the scope of this study. The point we want to make is that we indeed found in the genome assembly these genes from this especially important secondary pathway.
