# Supplementary material for: Genome assembly of the Pink Ipê (Handroanthus impetiginosus, Bignoniaceae), a highly valued, ecologically keystone Neotropical timber forest tree
Source: Gigascience. 2017 Dec 13;7(1):gix125. doi: 10.1093/gigascience/gix125 (PMC5905499; doi:10.1093/gigascience/gix125)
Supplement: Response_to_Reviewer_Comments_Revision_1.pdf [file gix125_response_to_reviewer_comments_revision_1.pdf]

Author's Response To Reviewer Comments

Dr Hans Zauner
Assistant Editor
GigaScience

We double checked the manuscript and perform minor corrections.
We also moved Figure S1 (ipê photo) to the main-text (it is now Figure 1) and change figure and supplemental material numbers.

Hope our changes are satisfactory.

All the best
Rosane Collevatti
