# Supplementary material for: Genome assembly of the Pink Ipê (Handroanthus impetiginosus, Bignoniaceae), a highly valued, ecologically keystone Neotropical timber forest tree
Source: Gigascience. 2017 Dec 13;7(1):gix125. doi: 10.1093/gigascience/gix125 (PMC5905499; doi:10.1093/gigascience/gix125)
Supplement: Reviewer_1_Report_(Original_Submission) -- Meg Staton — 09 Aug 2017 Reviewed [file gix125_reviewer_1_report_(original_submission).pdf]

Reviewer Report

Title: Genome assembly of the pink Ipê (Handroanthus impetiginosus, Bignoniaceae), a highly-valued ecologically keystone Neotropical timber forest tree

Version: Original Submission **Date:** 8/9/2017

Reviewer name: Meg Staton

Reviewer Comments to Author:

Da Silva-Junior et al report on a reference genome assembly for the pink Ipe. The methodology is reasonable for the project and includes both assembly and annotation. The text is very thorough and clear. The main problem I have is not being able to access the data on NCBI. I can see two biosamples and a bioproject, but I cannot find the SRA records, the genome or the annotations. The biosample record page will usually have a link at the bottom to the SRA, but this is missing (both only link back to the bioproject). Further if you search by the taxonomic name of the tree through Entrez, the number of records for genome is 0 and for SRA is also 0. Possibly the authors are waiting for final release until this is published? But this definitely needs to be taken care of prior to publication.A few edits/questionsLines 91-92 "beyond a relatively small numbers of microsatellites with their caveats for more sophisticated genetic analyses."Numbers should be number and also clarification of what the word caveats is referring to.Lines 112-113 - mention of other species being sequenced, but this isn't addressed in the results. Should remove from methods if these results are not addressed in this manuscriptLine 115 - Min should be MiniLine 123 - I'm not sure if "jump" is the official word or just jargon, maybe consider "fragment lengths of"Line 133 - mention of a "perl script" - this needs to be made available through github or somewhere elseLine 139 - same comment about "jumping" - I would consider changing to "mate pair"Line 402 - "showed" to something else, perhaps "illustrated"Line 432 - adequately doesn't really make sense in this sentence, perhaps removeLine 439 - The citation to Fig S6A doesn't make much sense, the text refers to blast results but the figure shows Gene ontology terms (and the figure is cited later in the GO section), maybe a supplemental figure is missing?Line 477 - Its not clear how the search for the genes was done (BLAST?)

Level of Interest

Please indicate how interesting you found the manuscript: An article whose findings are important to those with closely related research interests

Quality of Written English

Please indicate the quality of language in the manuscript: Acceptable

Declaration of Competing Interests

Please complete a declaration of competing interests, considering the following questions:

- Have you in the past five years received reimbursements, fees, funding, or salary from an organisation that may in any way gain or lose financially from the publication of this manuscript, either now or in the future?
- Do you hold any stocks or shares in an organisation that may in any way gain or lose financially from the publication of this manuscript, either now or in the future?
- Do you hold or are you currently applying for any patents relating to the content of the manuscript?
- Have you received reimbursements, fees, funding, or salary from an organization that holds or has applied for patents relating to the content of the manuscript?
- Do you have any other financial competing interests?
- Do you have any non-financial competing interests in relation to this paper?

If you can answer no to all of the above, write 'I declare that I have no competing interests' below. If your reply is yes to any, please give details below.

I declare that I have no competing interests

I agree to the open peer review policy of the journal. I understand that my name will be included on my report to the authors and, if the manuscript is accepted for publication, my named report including any attachments I upload will be posted on the website along with the authors' responses. I agree for my report to be made available under an Open Access Creative Commons CC-BY license (http://creativecommons.org/licenses/by/4.0/). I understand that any comments which I do not wish to be included in my named report can be included as confidential comments to the editors, which will not be published.

I agree to the open peer review policy of the journal
