# Supplementary material for: Genome assembly of the Pink Ipê (Handroanthus impetiginosus, Bignoniaceae), a highly valued, ecologically keystone Neotropical timber forest tree
Source: Gigascience. 2017 Dec 13;7(1):gix125. doi: 10.1093/gigascience/gix125 (PMC5905499; doi:10.1093/gigascience/gix125)
Supplement: Reviewer_2_Report_(Original_Submission) -- France Denoeud — 11 Aug 2017 Reviewed [file gix125_reviewer_2_report_(original_submission).pdf]

Reviewer Report

Title: Genome assembly of the pink Ipê (Handroanthus impetiginosus, Bignoniaceae), a highly-valued ecologically keystone Neotropical timber forest tree

Version: Original Submission **Date:** 8/11/2017

Reviewer name: France Denoeud

Reviewer Comments to Author:

The article describes the genome assembly of Handroanthus impetiginosus, a neotropical timber tree. Because of heterozygosity in the diploid genome that was sequenced, the final assembly is fragmented (N50=81,316bp, L50=1906). The assembly fragmentation might be an issue for future analyses, and the authors should be more specific about that. Some parts of the text should be rewritten in order to acknowledge the fact that the assembly obtained is highly fragmented. For instance, the sentence (line 337) "Our genome assembly metrics are similar to recent reports of genome assemblies of other highly heterozygous forest tree genomes", should be discarded for two reasons: first, if other heterozygous genomes were assembled in a highly fragmented way, the authors should not be satisfied with doing "as bad", but should aim at doing better. Second, the metrics obtained for Quercus robur were actually better that those obtained for pink Ipê (N50=260kb, L50=1468) (Plomion et al).The article is well written and very detailed. It describes analyses that confirm findings already observed in plant genomes, which are useful in the aim of validating the assembly and annotation processes. The section describing the metabolism of quinoid systems is of particular interest and opens avenues for future investigations.Below are more detailed comments on the manuscriptAbstract, line 47 : the terms "redundancy in the consensus determination" are not clear. Figure 2B shows that most scaffolds correspond to a consensus between the two haplotypes. What does "redundancy" means? Does it mean that for some parts of the genome the two haplotypes were assembled separately? The sentence should be clarified.Line 156: Figure S1 is called but it does not correspond to the pipeline (it should be figure S3).Along the manuscript numerous calls to figures do not correspond to the actual list of figures: the numbering and calling of figures should be checked carefully.Line 262 (316,table 1): The metrics (N50, L50…) are most of the time given for the whole assembly and for sequences longer than 20kb. What is the size of the smaller scaffolds in the assembly (was a threshold set)? It would be interesting to provide metrics (in table 1 also) for scaffolds >1kb or >2kb : how much of the genome is included in such scaffolds? Filtering out short scaffolds from the assembly should be envisaged, since no genes can be annotated in short scaffolds. It would have the advantage of providing better assembly metrics.Line 285: the N50 is given for scaffolds >1kb, and not for all scaffolds: is it possible to provide the same metrics for all assemblies in order to be able to compare them?Lines 281-288: What proportion of the sequence-coverage differences called by REAPR correspond to boundaries between regions where alleles were assembled separately vs collapsed ? If most of those errors are due to heterozygosity, would it make sense not to use the coverage information to break the scaffolds? Is there an option in REAPR in order to avoid the breaks? The procedure to filter out redundant copies of unmerged haplotypes might then require to split scaffolds, but it might result in less breaks in the assembly. Can the authors discuss on

that ?Lines 328, 332 : Figure 1C and D are called but the described figures correspond to Figure 2A and B.Line 348: know -> knownLine 352: Mostly -> MostLine 356: "expand a wide range of sequence sizes" : the sentence should be correctedLine 361: "unknown non-classifed" sequences : are not the two terms redundant?Line 366 : Figure 2 is called, it should be Figure 3Line 390: 31,668 genes were annotated. This number is relatively high for a plant genome. It would be interesting to explore paralogous gene clusters : are there duplicated genes in the genome? Are these genes more likely to have arisen from WGDs or tandem repeats ? If such analyses are possible despite the fragmented nature of the assembly, they would be of great interest.Line 439 : Figure S6 -> Figure 4A ?Line 446: Figure 3B -> Figure 4B ?Lines 451-452: BUSCO results were benchmarked using poplar. Poplar is known to have undergone a Whole Genome Duplication; Was the duplication status of Handroanthus investigated ? If there was no WGD, the duplicate level is probably not comparable to that of Populus.Lines 454-464: GO terms were compared to those of poplar. Why was such a distant species chosen for the comparison ? What about a comparison with other asterids, or even lamids (E. guttata, or Olea) ?Lines 482-487: The authors report that some steps of the quinoid metabolism are encoded by more genes in pink Ipê than in other species. It would be interesting to elucidate how these genes were amplified in the Hydroanthus genome : is it possible to build a phylogeny of these genes ? Are some of them located on the same scaffold ? (are they possibly deriving from tandem duplications?...)

Level of Interest

Please indicate how interesting you found the manuscript: An article of importance in its field

Quality of Written English

Please indicate the quality of language in the manuscript: Acceptable

Declaration of Competing Interests

Please complete a declaration of competing interests, considering the following questions:

- Have you in the past five years received reimbursements, fees, funding, or salary from an organisation that may in any way gain or lose financially from the publication of this manuscript, either now or in the future?
- Do you hold any stocks or shares in an organisation that may in any way gain or lose financially from the publication of this manuscript, either now or in the future?
- Do you hold or are you currently applying for any patents relating to the content of the manuscript?
- Have you received reimbursements, fees, funding, or salary from an organization that holds or has applied for patents relating to the content of the manuscript?
- Do you have any other financial competing interests?
- Do you have any non-financial competing interests in relation to this paper?

If you can answer no to all of the above, write 'I declare that I have no competing interests' below. If your reply is yes to any, please give details below.

I declare that I have no competing interests

I agree to the open peer review policy of the journal. I understand that my name will be included on my report to the authors and, if the manuscript is accepted for publication, my named report including any attachments I upload will be posted on the website along with the authors' responses. I agree for my report to be made available under an Open Access Creative Commons CC-BY license (http://creativecommons.org/licenses/by/4.0/). I understand that any comments which I do not wish to be included in my named report can be included as confidential comments to the editors, which will not be published.

I agree to the open peer review policy of the journal
